# Supplementary material for: An investigation of bubble resonance and its implications for sound production by deep-water fishes
Source: PLoS One. 2022 Jul 12;17(7):e0267338. doi: 10.1371/journal.pone.0267338 (PMC9275728; doi:10.1371/journal.pone.0267338)
Supplement: S2 File — PDF file created from S1 File. This file does not require Jupyter or Julia for viewing. (PDF) [file pone.0267338.s002.pdf]

Deep Water Liquid and Gas Properties

1. Introduction

This Jupyter notebook performs the calculations of the liquid and gas properties at pressures and temperatures that vary from the water surface to the deep ocean (3500 m depth and 1.5 °C temperature) for use in modeling bubble oscillations and sound production by fish swim bladders for the paper Mark W. Sprague, Michael L. Fine, and Timothy M. Cameron (2022), "An investigation of bubble resonance and its implications for sound production by deep-water fishes." Julia version 1.7.2 was used for the calculations. Symbolic calculations use the SymPy package (which calls the Python SymPy package). Symbolic expressions are converted to Julia functions to calculate numerical values. For each parameter we calculate values for the warm surface (temperature 20 °C, salinity 35 g/kg), cold surface (temperature 1.5 °C, salinity 35 g/kg), and 3500 m deep (temperature 1.5 °C, salinity 35 g/kg) environments. We also calculate values a 1 m depth intervals for an ocean with constant temperature 1.5 °C and salinity 35 g/kg. The depth profile values required for our acoustic calculations are stored in dataframes in tabular form exported to CSV files. See the main paper for all references cited here.

This notebook uses the following Julia packages.

- SymPy - symbolic calculations.
- Roots - numerical root finding.
- DataFrames - store tabulated values in a dataframe for export.
- CSV - export dataframes with tabulated values into a CSV file.

Many of the symbolic calculations in this notebook produce long symbolic expressions as output. These expressions are many lines long (sometimes over a page long) and often do not fit within the page margins or screen size. Output from these expressions has been suppressed using a trailing semicolon (`;`). To view the output, delete the trailing semicolon before entering the input cell.

2. Initializations

Load the necessary packages and import the symbols defined by `SymPy`.

[1]:
```
using SymPy, Roots, CSV, DataFrames
import_from(sympy)
```

3. Parameters

Define some parameters.

[2]:
```
tcold = 1.5; # °C, cold water temperature
```

[3]:
```
Tcold = tcold + 273.15; # K, cold water absolute temperature
```

[4]:
```
sal = 35.0; # g/kg, salinity
```

[5]:
```
ts = 20.0; # °C, warm surface temperature
```

[6]:
```
Ts = 293.15; # K, warm surface absolute temperature
```

[7]:
```
ps = 1.01325e5; # Pa, surface pressure
```

[8]:
```
ϕ0 = 30.0 * pi/180; # degrees, lattitude for gravitational acceleration
    # term in pressure-depth calculations
```

[9]:
```
dvals = 0:3500; # m, range of depth values for calculation
```

Note that the array `dvals` contains depths at 1 m intervals from 0 m to 3500 m. A depth `d` that is a whole number value in meters is at index `d + 1` in `dvals`. This is useful because we do not have to repeat indexed calculations based on `dvals` when we need values at specific depths.

For calculations a depths 1000 m and 2000 m, locate the `dvals` elements that represent these depths.

[10]:
```
dvals[1001]
```

[10]:
```
1000
```

[11]:
```
dvals[2001]
```

[11]:
```
2000
```

Initialize dataframes for storing tabulated parameter values. The dataframe `valso2` will hold the water and oxygen gas properties required for the acoustic calculations, and the dataframe `valsn2` will hold the water and nitrogen gas properties required for the acoustic calculations.

[12]:
```
valso2 = DataFrame(depth=dvals);
valsn2 = DataFrame(depth=dvals);
```

4. Liquid Properties

These are the relations for the properties of the liquid (seawater).

Define some symbols.

[13]:
```
@vars ϕ p P z S Sfrac t pPa
```

[13]:
```
(ϕ, p, P, z, S, Sfrac, t, pPa)
```

4.1. Pressure vs. Depth

Pressure vs. depth relationship from Saunders and Fofonoff [52].

Define parameters used in calculation.

[14]:
```
cd = [0.712953, 1.113e-7, -3.434e-12, 14190.7];
```

The expression below is for the effective gravitational acceleration in terms of the latitude angle `ϕ`.

[15]:
```
g0 = 9.780318 * (1 + 5.3024e-3 * sin(ϕ)^2 - 5.9e-6 * sin(2 * ϕ)^2) # m/s^2,
↪gravitational acceleration
```

[15]:

$$0.0518591581632 \sin^2(\phi) - 5.77038762 \cdot 10^{-5} \sin^2(2\phi) + 9.780318$$

This is the gravitational acceleration at latitude $\phi_0 = 30^\circ$ N.

[16]:
```
g0(ϕ=>ϕ0)
```

[16]:

$$9.79323951163365$$

[17]:
```
pg = p - ps # Pa, pressure gradient with surface
```

[17]:

$$p - 101325.0$$

[18]:
```
pdb = pg/10^4 # dbar, pressure in decibars; used to compare values in the reference
```

[18]:

$$\frac{p}{10000} - 10.1325$$

[19]:
```
γprime = 2.226e-6; # m s^-2 dbar^-1
```

We ignore the ΔD term Eq. (4) in Saunders and Fofonoff [52] to use a standard ocean.

[20]:
```
ΔD = 0; # Ignore this term.
```

This is Eq. (4) in Saunders and Fofonoff [52] with the pressure `p` in Pa.

Output from the following symbolic calculation has been suppressed. Remove the trailing semicolon before entering the cell to display it.

[21]:
```
zpeq = Eq(z, simplify(sum(((cd .* (pdb, pdb^2, pdb^3, log(1 + 1.83e-5 * pdb))) ./
    ((100 * g0 + 1/2 * γprime * pdb) * 10^-3) .+ ΔD /
    0.98))));
```

Calculate depth for various pressure gradients to compare with the value in Table 1 in Saunders and Fofonoff [52].

[22]:
```
zpeq(ϕ => ϕ0, p => 1.01325e5 + 500e4)
```

[22]:

$$z = 496.013686442597$$

[23]:
```
zpeq(ϕ => ϕ0, p => 1.01325e5 + 1000e4)
```

[23]:

$$z = 990.88969207793$$

[24]:
```
zpeq(ϕ => ϕ0, p => 1.01325e5 + 3000e4)
```

[24]:

$$z = 2959.38223978458$$

[25]:
```
zpeq(ϕ => ϕ0, p => 1.01325e5 + 4000e4)
```

[25]:

$$z = 3937.26224667999$$

[26]:
```
(3937.26224667999 - 3935.49)/3935.49 * 100
```

[26]:
```
0.045032427473836185
```

These values are consistent with the table to better than 0.05%, which is OK for our calculations.

Now create a Julia function we can solve numerically.

[27]:
```
zpjl = lambdify(lhs(zpeq) - rhs(zpeq),(ϕ, z, p))
```

[27]:
```
#118 (generic function with 1 method)
```

The function `psub` gives the pressure at a given depth.

[28]:
```
psub(ϕ, z, p0=100.e5) = find_zero(p1 -> zpjl(ϕ, z, p1), p0)
```

[28]:
```
psub (generic function with 2 methods)
```

Create a Julia function that converts the calculated pressure to dbar so we can compare calculations to Table 1 in Saunders and Fofonoff [52].

[29]:
```
pdbjl = lambdify(pdb, (p,))
```

[29]:
```
#118 (generic function with 1 method)
```

Now test this for some values in Table 1 in in Saunders and Fofonoff [52].

[30]:
```
pdbjl(psub(ϕ0, 495.99))
```

[30]:
```
499.97609563134597
```

[31]:
```
pdbjl(psub(ϕ0, 990.77))
```

[31]:
```
999.8789310233103
```

[32]:
```
pdbjl(psub(30*pi/180, 2958.38))
```

[32]:
```
2998.9772647391114
```

[33]:
```
pdbjl(psub(30*pi/180, 3935.49))
```

[33]:
```
3998.183851177998
```

[34]:
```
(4000 - pdbjl(psub(30*pi/180, 3935.49))) / 4000 * 100
```

[34]:
```
0.04540372055005264
```

These values are also consistent with those in Table 1 in Saunders and Fofonoff [52] to better than 0.05%.

Create a table of pressures at the depths in `dvals`.

Warm surface pressure.

[35]:
```
psub(ϕ0, 0)
```

[35]:
```
101325.0
```

Cold surface pressure.

[36]:
```
psub(ϕ0, 0)
```

[36]:
```
101325.0
```

Pressure at depth 1000 m.

[37]:
```
psub(ϕ0, 1000)
```

[37]:
```
1.0193478046816997e7
```

Pressure at depth 2000 m.

[38]:
```
psub(ϕ0, 2000)
```

[38]:
```
2.0331946613939572e7
```

Deep water pressure.

[39]:
```
psub(ϕ0, 3500)
```

[39]:
```
3.562456759610306e7
```

[40]:
```
ptab = map(d1 -> psub(30*pi/180, d1), dvals);
```

[41]:
```
ptab[1], ptab[end]
```

[41]:
```
(101325.0, 3.562456759610306e7)
```

Store these pressures in the two dataframes.

[42]:
```
valso2.pressure = ptab;
valsn2.pressure = ptab;
```

4.2. Density

We calculate seawater density using Eqs. (7) from Sharqaway *et al.* [53].

Define the parameters in the equation.

[43]:
```
arho = (9.992e2, 9.539e-2, -2.581e-5, 3.131e-5, -6.174e-8,
  4.337e-1, 2.549e-5, -2.899e-7, 9.578e-10, 1.763e-3, -1.231e-4,
   1.366e-6, 4.045e-9, -1.467e-5, 8.839e-7, -1.102e-9,
  4.247e-11, -3.959e-14)
```

[43]: (999.2, 0.09539, -2.581e-5, 3.131e-5, -6.174e-8, 0.4337, 2.549e-5, -2.899e-7, 9.578e-10, 0.001763, -0.0001231, 1.366e-6, 4.045e-9, -1.467e-5, 8.839e-7, -1.102e-9, 4.247e-11, -3.959e-14)

[44]:
```
brho = (-7.999e-1, 2.409e-3, -2.581e-5, 6.856e-8,
  6.298e-4, -9.363e-7)
```

[44]: (-0.7999, 0.002409, -2.581e-5, 6.856e-8, 0.0006298, -9.363e-7)

This is Eq. (7) from Sharqaway *et al.* [53]. Note that the pressure `p` is in MPa, and the temperature `t` is in °C. Note that the stated accuracy of this equation is ±2.5%.

Output from the following symbolic calculation has been suppressed. Remove the trailing semicolon before entering the cell to display it.

[45]:
```
ρsw = simplify(sum(arho .* (1, t, t^2, t^3, t^4, p, p*t^2, p*t^3,
    p*t^4, p^2, p^2*t, p^2*t^2, p^2*t^3, p^3, p^3*t, p^3*t^2,
    p^3*t^3, p^3*t^4)) - sum(brho .* (S, S*t, S*t^2, S*t^3, S*p,
    S*p^2)));
```

[46]:
```
ρsw(t=>15, p=>1.01325e5/1e6, S=>0)
```

[46]: 1000.77202240146

[47]:
```
ρsw(t=>20, p=>1.01325e5/1e6, S=>35)
```

[47]: 1028.03294469513

[48]:
```
ρsw(t=>tcold, p=>ptab[end]/1e6, S=>sal)
```

[48]: 1043.32748758591

These values are reasonable given the accuracy of the equation.

Create a Julia function to calculate seawater density.

[49]:
```
ρswjl = lambdify(ρsw(p=>pPa/1e6), (t, pPa, S))
```

[49]: #118 (generic function with 1 method)

Warm surface density.

[50]:
```
ρswjl(ts, ptab[1], sal)
```

[50]: 1028.032944695128

Cold surface density.

[51]:
```
ρswjl(tcold, ptab[1], sal)
```

[51]: 1027.2569176419536

Density at depth 1000 m (`dvals` index 1001)

[52]:
```
ρswjl(tcold, ptab[1001], sal)
```

[52]:
```
1031.5655667337887
```

Density at depth 2000 m (`dvals` index 2001)

[53]:
```
ρswjl(tcold, ptab[2001], sal)
```

[53]:
```
1036.1412358223129
```

Deep water density

[54]:
```
ρswjl(tcold, ptab[end], sal)
```

[54]:
```
1043.3274875859083
```

Tabulate density values for the depths in `dvals`.

[55]:
```
ρswtab = map(p1 -> ρswjl(tcold, p1, sal), ptab);
```

[56]:
```
ρswtab[1], ρswtab[end]
```

[56]:
```
(1027.2569176419536, 1043.3274875859083)
```

Add these values to the two dataframes.

[57]:
```
valso2.water_density = ρswtab
valsn2.water_density = ρswtab;
```

4.3. Dynamic Viscosity

We calculate the dynamic viscosity of seawater using Eqs. (22) and (23) from Sharqaway *et al.* [53].

This is Eq. (23).

[58]:
```
μw = 4.2844e-5 + (0.157*(t + 64.993)^2 - 91.296)^-1
```

[58]:

$$4.2844 \cdot 10^{-5} + \frac{1}{663.182137693(0.0153862723677935t+1)^2 - 91.296}$$

[59]:
```
Amu = 1.541 + 1.998e-2*t - 9.52e-5*t^2
```

[59]:

$$-9.52 \cdot 10^{-5}t^2 + 0.01998t + 1.541$$

[60]:
```
Bmu = 7.974 - 7.561e-2*t + 4.724e-4*t^2
```

[60]:

$$0.0004724t^2 - 0.07561t + 7.974$$

This is Eq. (22). The salinity value `Sfrac` in the following equation is in kg/kg. We need to divide the traditional salinity in g/kg by 1000 to convert it.

Output from the following symbolic calculation has been suppressed. Remove the trailing semicolon before entering the cell to display it.

[61]:
```
μsw = μw*(1 + Amu*Sfrac + Bmu*Sfrac^2);
```

Create a Julia function for dynamic viscosity.

[62]:
```
μswjl = lambdify(μsw(Sfrac=>S/1000), (t, S))
```

[62]: `#118 (generic function with 1 method)`

Warm surface value

[63]:
```
μswjl(ts, sal)
```

[63]: `0.0010766289252529318`

Cold surface value

[64]:
```
μswjl(tcold, sal)
```

[64]: `0.0018115654847495556`

Value at depth 1000 m

[65]:
```
μswjl(tcold, sal)
```

[65]: `0.0018115654847495556`

Value at depth 2000 m

[66]:
```
μswjl(tcold, sal)
```

[66]: `0.0018115654847495556`

Deep water value

[67]:
```
μswjl(tcold, sal)
```

[67]: `0.0018115654847495556`

This parameter only depends on temperature and salinity, which we are holding constant for this depth profile. Calculate the constant value and multiply it by the `ones` vector.

[68]:
```
μswtab = μswjl(tcold, sal) .* ones(length(dvals));
```

Store these values in the dataframes.

[69]:
```
valso2.water_dyn_viscosity = μswtab
valsn2.water_dyn_viscosity = μswtab;
```

4.4. Surface Tension

We use Eqs. (27) and (28) from Sharqaway *et al.* [53] for the water surface tension.

This is Eq. (27).

Output from the following symbolic calculation has been suppressed. Remove the trailing semicolon before entering the cell to display it.

[70]:
```
σw = 0.2358 * (1 - (t + 273.15)/647.096)^1.256 * (1 - 0.625*(1 - (t
    + 273.15)/647.096));
```

This is Eq. (38) solved for the seawater surface tension.

Output from the following symbolic calculation has been suppressed. Remove the trailing semicolon before entering the cell to display it.

[71]:
```
σsw = (1 + (0.000226*t + 0.00946) * log(1 + 0.0331 * S)) * σw;
```

Create a Julia function.

[72]:
```
σswjl = lambdify(σsw, (t, S))
```

[72]: `#118 (generic function with 1 method)`

Warm surface value

[73]:
```
σswjl(ts, sal)
```

[73]: `0.0735185195321562`

Cold surface value

[74]:
```
σswjl(tcold, sal)
```

[74]: `0.07600619501340314`

Value at depth 1000 m

[75]:
```
σswjl(tcold, sal)
```

[75]: `0.07600619501340314`

Value at depth 2000 m

[76]:
```
σswjl(tcold, sal)
```

[76]: `0.07600619501340314`

Deep water value

[77]:
```
σswjl(tcold, sal)
```

[77]: 0.07600619501340314

This parameter only depends on temperature and salinity, which we are holding constant for this depth profile. Calculate the constant value and multiply it by the `ones` vector.

[78]:
```
σswtab = σswjl(tcold, sal) .* ones(length(dvals));
```

Add these values to the dataframes.

[79]:
```
valso2.water_surface_tension = σswtab
valsn2.water_surface_tension = σswtab;
```

4.5. Sound Speed

We calculate sound speed from the UN equation in Wong and Zhu [54].

The parameter `P` is the pressure in bar. (1 bar = 105 Pa.)

Define the other parameters in the equation.

[80]:
```
Asw = (
    (1.389, -1.262e-2, 7.166e-5, 2.008e-6, -3.21e-8),
    (9.4742e-5, -1.2583e-5, -6.4928e-8, 1.0515e-8, -2.0142e-10),
    (-3.9064e-7, 9.1061e-9, -1.6009e-10, 7.994e-12),
    (1.100e-10, 6.651e-12, -3.391e-13)
)
```

[80]: ((1.389, -0.01262, 7.166e-5, 2.008e-6, -3.21e-8), (9.4742e-5, -1.2583e-5, -6.4928e-8, 1.0515e-8, -2.0142e-10), (-3.9064e-7, 9.1061e-9, -1.6009e-10, 7.994e-12), (1.1e-10, 6.651e-12, -3.391e-13))

[81]:
```
Bsw = ((-1.922e-2, -4.42e-5), (7.3637e-5, 1.7950e-7))
```

[81]: ((-0.01922, -4.42e-5), (7.3637e-5, 1.795e-7))

[82]:
```
Dsw = (1.727e-3, -7.9836e-6)
```

[82]: (0.001727, -7.9836e-6)

[83]:
```
Cww = ((1402.388, 5.03830, -5.81090e-2, 3.3432e-4, -1.47797e-6,
    3.1419e-9), (0.153563, 6.8999e-4, -8.1829e-6, 1.3632e-7,
    -6.1260e-10), (3.1260e-5, -1.7111e-6, 2.5986e-8, -2.5353e-10,
    1.0415e-12), (-9.7729e-9, 3.8513e-10, -2.3654e-12))
```

[83]: ((1402.388, 5.0383, -0.058109, 0.00033432, -1.47797e-6, 3.1419e-9), (0.153563, 0.00068999, -8.1829e-6, 1.3632e-7, -6.126e-10), (3.126e-5, -1.7111e-6, 2.5986e-8, -2.5353e-10, 1.0415e-12), (-9.7729e-9, 3.8513e-10, -2.3654e-12))

Output from the following symbolic calculation has been suppressed. Remove the trailing semicolon before entering the cell to display it.

[84]:
```
AtP = (sum(Asw[1] .* (1, t, t^2, t^3, t^4)) +
  sum(Asw[2] .* (1, t, t^2, t^3, t^4)) * P +
  sum(Asw[3] .* (1, t, t^2, t^3)) * P^2 + sum(Asw[4] .* (1, t, t^2)) * P^3);
```

[85]:
```
BtP = sum(Bsw[1] .* (1, t)) + sum(Bsw[2] .* (1, t)) * P
```

[85]:

$$P\left(1.795 \cdot 10^{-7}t + 7.3637 \cdot 10^{-5}\right) - 4.42 \cdot 10^{-5}t - 0.01922$$

[86]:
```
DtP = sum(Dsw .* (1, P))
```

[86]:

$$0.001727 - 7.9836 \cdot 10^{-6}P$$

Output from the following symbolic calculation has been suppressed. Remove the trailing semicolon before entering the cell to display it.

[87]:
```
CwtP = sum(Cww[1] .* (1, t, t^2, t^3, t^4, t^5)) + sum(Cww[2] .* (1,
    t, t^2, t^3, t^4)) * P + sum(Cww[3] .* (1, t, t^2, t^3, t^4)) *
    P^2 + sum(Cww[4] .* (1, t, t^2)) * P^3;
```

The following expression is the sound speed as a function of temperature, salinity, and pressure.

Output from the following symbolic calculation has been suppressed. Remove the trailing semicolon before entering the cell to display it.

[88]:
```
CStP = CwtP + AtP*S + BtP*S^(3/2) + DtP*S^2;
```

Create a Julia function with the pressure value in Pa.

[89]:
```
CtSpjl = lambdify(CStP(P=>p/1e5),(t, S, p))
```

[89]:
```
#118 (generic function with 1 method)
```

Warm surface value

[90]:
```
CtSpjl(ts, sal, ptab[1])
```

[90]:
```
1521.6469588481918
```

Cold surface value

[91]:
```
CtSpjl(tcold, sal, ptab[1])
```

[91]:
```
1456.0611774871181
```

Value at depth 1000 m (index 1001 in `dvals`)

[92]:
```
CtSpjl(tcold, sal, ptab[1001])
```

[92]:
```
1472.6428237138698
```

Value at depth 2000 m (index 2001 in `dvals`)

[93]:
```
CtSpjl(tcold, sal, ptab[2001])
```

[93]: 1489.5878214658405

Deep water value

[94]:
```
CtSpjl(tcold, sal, ptab[end])
```

[94]: 1515.6224501126085

Calculate values for the depth profile.

[95]:
```
ctab = map(p1 -> CtSpjl(tcold, sal, p1), ptab);
```

[96]:
```
ctab[1], ctab[end]
```

[96]: (1456.0611774871181, 1515.6224501126085)

Add these values to the dataframes.

[97]:
```
valso2.water_sound_speed = ctab
valsn2.water_sound_speed = ctab;
```

5. Gas Properties

[98]:
```
R = 8.314510 # J mol^-1 K^-1, universal gas constant
```

[98]: 8.31451

5.1. Oxygen

We calculate oxygen densities, heat capacities, and sound speeds using the relationships from Schmidt and Wagner [56]. We calculate thermal conductivities using the relationships in Lemmon and Jacobsen [58]. We calculate thermal diffusivities using the relationship in Ainslie and Leighton [1].

Define some gas parameters used by Schmidt and Wagner [56].

[99]:
```
MO2 = 31.9988/1000; # kg, O_2 Molar mass
```

[100]:
```
T0 = 298.15; # K
```

[101]:
```
p0 = 1.01325e5; # Pa
```

[102]:
```
ρc = 13.63e3; # mol/m^3
```

[103]:
```
Tc = 154.581; # K
```

[104]:
```
δ0 = p0 /(R * T0 * ρc);
```

[105]:
```
τ0 = Tc / T0;
```

Define variables used in symbolic calculations.

[106]:
```
@vars δ τ T ρ M
```

[106]: (δ, τ, T, ρ, M)

Define the coefficients in the equations.

[107]:
```
no2 = (0.3983768749, -0.1846157454e1, 0.4183473197, 0.2370620711e-1,
    0.9771730573e-1, 0.3017891294e-1, 0.2273353212e-1,
    0.1357254086e-1, -0.4052698943e-1, 0.5454628515e-3,
    0.5113182277e-3, 0.2953466883e-6, -0.8687645072e-4,
    -0.2127082589, 0.8735941958e-1, 0.1275509190, -0.9067701064e-1,
    -0.3540084206e-1, -0.3623278059e-1, 0.1327699290e-1,
    -0.3254111865e-3, -0.8313582932e-2, 0.2124570559e-2,
    -0.8325206232e-3, -0.2626173276e-4, 0.2599581482e-2,
    0.9984649663e-2, 0.2199923153e-2, -0.2591350486e-1,
    -0.1259630848, 0.1478355637, -0.1011251078e-1)
```

[107]: (0.3983768749, -1.846157454, 0.4183473197, 0.02370620711, 0.09771730573, 0.03017891294, 0.02273353212, 0.01357254086, -0.04052698943, 0.0005454628515, 0.0005113182277, 2.953466883e-7, -8.687645072e-5, -0.2127082589, 0.08735941958, 0.127550919, -0.09067701064, -0.03540084206, -0.03623278059, 0.0132769929, -0.0003254111865, -0.008313582932, 0.002124570559, -0.0008325206232, -2.626173276e-5, 0.002599581482, 0.009984649663, 0.002199923153, -0.02591350486, -0.1259630848, 0.1478355637, -0.01011251078)

[108]:
```
kko2 = (-0.740775e-3, -0.664930e-4, 0.250042e1, -0.214487e2,
    0.101258e1, -0.944365, 0.145066e2, 0.749148e2, 0.414817e1)
```

[108]: (-0.000740775, -6.6493e-5, 2.50042, -21.4487, 1.01258, -0.944365, 14.5066, 74.9148, 4.14817)

[109]:
```
ro2 = (1, 1, 1, 2, 2, 2, 3, 3, 3, 6, 7, 7, 8, 1, 1, 2, 2, 3, 3, 5, 6,
    7, 8, 10, 2, 3, 3, 4, 4, 5, 5, 5)
```

[109]: (1, 1, 1, 2, 2, 2, 3, 3, 3, 6, 7, 7, 8, 1, 1, 2, 2, 3, 3, 5, 6, 7, 8, 10, 2, 3, 3, 4, 4, 5, 5, 5)

[110]:
```
so2 = (0, 1.5, 2.5, -0.5, 1.5, 2, 0, 1, 2.5, 0, 2, 5, 2, 5, 6, 3.5,
    5.5, 3, 7, 6, 8.5, 4, 6.5, 5.5, 22, 11, 18, 11, 23, 17, 18, 23)
```

[110]: (0, 1.5, 2.5, -0.5, 1.5, 2, 0, 1, 2.5, 0, 2, 5, 2, 5, 6, 3.5, 5.5, 3, 7, 6, 8.5, 4, 6.5, 5.5, 22, 11, 18, 11, 23, 17, 18, 23)

The following is Eq. (15) in Schmidt and Wagner [56] with Φ^{id} changed to `α0`.

Output from the following symbolic calculation has been suppressed. Remove the trailing semicolon before entering the cell to display it.

[111]:
```
α0 = (kko2[1]*τ^1.5 + kko2[2]*τ^-2 + kko2[3]*log(τ) + kko2[4]*τ +
    kko2[5]*log(exp(kko2[7]*τ) - 1) + kko2[6]*log(1 +
    2/3*exp(-kko2[8]*τ)) + kko2[9] + log(δ/δ0));
```

The following is Eq (11) in Lemmon and Jacobsen [58] with Φ^r changed to `αr`.

Output from the following symbolic calculation has been suppressed. Remove the trailing semicolon before entering the cell to display it.

[112]:
```
αr = (
    sum(map(m -> no2[m] * δ^ro2[m] * τ^so2[m], 1:13)) +
    exp(-δ^2) *
    sum(map(m -> no2[m] * δ^ro2[m] * τ^so2[m], 14:24)) +
    exp(-δ^4) *
    sum(map(m -> no2[m] * δ^ro2[m] * τ^so2[m], 25:32))
);
```

5.1.1. Density

Find the molar density from the root of Eq. (A1) in Schmidt and Wagner [56]. The term `ρ` in this equation is molar density with SI unit mol/m3.

Output from the following symbolic calculation has been suppressed. Remove the trailing semicolon before entering the cell to display it.

[113]:
```
ρeq = Eq(p, ρ * R * T * (1 + δ * diff(αr, δ)))(δ => ρ/ρc, τ => Tc/T);
```

Create a Julia function that we can use to find roots numerically.

[114]:
```
ρeqjl = lambdify(lhs(ρeq) - rhs(ρeq), (p, T, ρ))
```

[114]:
```
#118 (generic function with 1 method)
```

Now create a function that numerically finds a root for the density at a given value of pressure and temperature.

[115]:
```
ρo2(p, T, ρ0=24000.0) = find_zero(ρ1 -> ρeqjl(p, T, ρ1), ρ0)
```

[115]:
```
ρo2 (generic function with 2 methods)
```

Now we comparing to densities tabulated by Weber [57] for O2.

Find the density at $p = 700$ bar (or 700×10^5 Pa) and $T = 270$ K.

[116]:
```
ρo2(700e5, 270)
```

[116]:
```
22886.84924777297
```

The Weber [57] gives a volume of 1.3657 cm3/g at this pressure and temperature (Table VIa). Convert this to density in mol/m3.

[117]:
```
(1/(1.3657 #= cm^3/g =#) * (1 #= kg =# / 1000 #= g =#) *
    (1 / MO2 #= kg/mol =#) * (100 #= cm =# / 1 #= m =#)^3 )
```

[117]:
```
22882.89662367062
```

The calculated value is consistent to 4 significant figures. This is good.

Now find the density at $p = 700$ bar (or 700×10^5 Pa) and $T = 300$ K.

[118]:
```
ρo2(700e5, 300)
```

[118]:
```
20920.324839551213
```

Weber [57] gives a volume of 1.4941 cm3/g (Table VIa). Convert this to density in mol/m3.

[119]:
```
(1/(1.4941 #= cm^3/g =#) * (1 #= kg =# / 1000 #= g =#) *
    (1 / MO2 #= kg/mol =#) * (100 #= cm =# / 1 #= m =#)^3 )
```

[119]:
```
20916.385729835325
```

This also agrees to four significant figures.

Now tabulate mass density values by multiplying each molar density by the molar mass.

[120]:
```
ρo2tab = map(p1 -> ρo2(p1, Tcold) * MO2, ptab);
```

The values below ate the mass densities for oxygen at the cold surface, depth 1000 m, depth 2000 m, and depth 3500 m.

[121]:
```
ρo2tab[1], ρo2tab[1001], ρo2tab[2001], ρo2tab[end]
```

[121]:
```
(1.4211670046377123, 154.7115917256005, 314.00236160237546, 500.93121320697526)
```

5.1.2. Isochoric Heat Capacity c_v

The following is Eq. (A6) from Schmidt and Wagner [56].

Output from the following symbolic calculation has been suppressed. Remove the trailing semicolon before entering the cell to display it.

[122]:
```
cv = -τ^2 * (diff(α0, τ, τ) +  diff(αr, τ, τ)) * R;
```

Substitute the values of δ and τ in terms of molar density and temperature.

Output from the following symbolic calculation has been suppressed. Remove the trailing semicolon before entering the cell to display it.

[123]:
```
cv(δ => ρ/ρc, τ => Tc/T);
```

Define a Julia function for calculations.

[124]:
```
cvjl = lambdify(cv(δ => ρ/ρc, τ => Tc/T),(ρ, T))
```

[124]: `#118 (generic function with 1 method)`

Calculate some values.

[125]:
```
cvjl(ρo2(700e5, 270), 270)
```

[125]: `23.898882530084624`

Define another Julia function that uses an input pressure and temperature to calculate the needed density value.

[126]:
```
cvpTo2(p, T) = cvjl(ρo2(p, T), T)
```

[126]: `cvpTo2 (generic function with 1 method)`

[127]:
```
cvpTo2(1e5, 300)
```

[127]: `21.078866720527625`

[128]:
```
cvpTo2(1e5, 270)
```

[128]: `20.95584051862463`

Weber [57] reports specific heat C_v values in J/(g K). Get these by dividing the `cvpTo2` values by the molar mass in g, which is 1000 `MO2`.

[129]:
```
cvpTo2(10e5, 300) / (1000 * MO2)
```

[129]: `0.660918602988702`

[130]:
```
cvpTo2(25e5, 300) / (1000 * MO2)
```

[130]: `0.6644992914305471`

These values are very close to Weber's results. They begin to diverge at higher pressures.

[131]:
```
cvpTo2(300e5, 300) / (1000 * MO2)
```

[131]: `0.7089086969553833`

5.1.3. Isobaric Heat Capacity c_p

Use Eq. (A7) from Schmidt and Wagner [56] to calculate c_p.

Output from the following symbolic calculation has been suppressed. Remove the trailing semicolon before entering the cell to display it.

[132]:
```
cp = (cv + (1 + δ * diff(αr, δ) - δ * τ *
    diff(αr, δ, τ))^2 / (1 + 2 * δ * diff(αr, δ) +
    δ^2 * diff(αr, δ, δ)) * R);
```

Define a Julia function to calculate values.

[133]:
```
cpjl = lambdify(cp(δ => ρ/ρc, τ => Tc/T),(ρ, T))
```

[133]:
```
#118 (generic function with 1 method)
```

Define another Julia function that uses an input pressure and temperature to calculate the needed density value.

[134]:
```
cppTo2(p, T) = cpjl(ρo2(p, T), T)
```

[134]:
```
cppTo2 (generic function with 1 method)
```

Calculate some values.

[135]:
```
cppTo2(1e5, 300)
```

[135]:
```
29.435205927984697
```

[136]:
```
cppTo2(1e5, 300) / (1000 * MO2)
```

[136]:
```
0.9198846809250565
```

Weber [57] reports specific heat C_p values in J/(g K). Get these by dividing the `cppTo2` values by the molar mass in g, which is 1000 `MO2`.

[137]:
```
cppTo2(10e5, 300) / (1000 * MO2)
```

[137]:
```
0.9340227201860647
```

[138]:
```
cppTo2(25e5, 300) / (1000 * MO2)
```

[138]:
```
0.9582613365905686
```

These values are very close to Weber's results. They begin to diverge at higher pressures.

[139]:
```
cppTo2(300e5, 300) / (1000 * MO2)
```

[139]:
```
1.3072282411638052
```

5.1.4. Ratio of Specific Heats γ

Use the values of the two specific heats to calculate $\gamma = c_p/c_v$.

[140]:
```
γo2(p, T) = cppTo2(p, T) / cvpTo2(p, T)
```

[140]: γo2 (generic function with 1 method)

Warm surface value

```
[141]: γo2(ptab[1], Ts)
```

[141]: 1.3971781560134038

Cold surface value

```
[142]: γo2(ptab[1], Tcold)
```

[142]: 1.398953943344819

Value at depth 1000 m (index 1001 in `dvals`)

```
[143]: γo2(ptab[1001], Tcold)
```

[143]: 1.6680921647387352

Value at depth 2000 m (index 2001 in `dvals`)

```
[144]: γo2(ptab[2001], Tcold)
```

[144]: 1.8968036632857108

Deep water value

```
[145]: γo2(ptab[end], Tcold)
```

[145]: 1.9497207685437572

Calculate values for the depth profile.

```
[146]: γo2tab = map(p1 -> γo2(p1, Tcold), ptab);
```

```
[147]: γo2tab[1], γo2tab[1001], γo2tab[2001], γo2tab[end]
```

[147]: (1.398953943344819, 1.6680921647387352, 1.8968036632857108, 1.9497207685437572)

Add these values to the dataframe.

```
[148]: valso2.gamma = γo2tab;
```

5.1.5. Sound Speed

Use Eq. (A8) from Schmidt and Wagner [56] to calculate the sound speed in oxygen.

Output from the following symbolic calculation has been suppressed. Remove the trailing semicolon before entering the cell to display it.

```
[149]: w = sqrt( R * T / M * (1 + 2 * δ * diff(αr, δ) + δ^2 * diff(αr, δ, δ) -
           (1 + δ * diff(αr, δ) - δ * τ * diff(αr, δ, τ))^2 / (τ^2 *
           (diff(α0, τ, τ) + diff(αr, τ, τ)))));
```

Define a Julia function.

```
[150]: wjl = lambdify(w(δ => ρ/ρc, τ => Tc/T),(ρ, T, M))
```

```
[150]: #118 (generic function with 1 method)
```

Define a function of pressure and temperature as before.

```
[151]: wpT(p, T) = wjl(ρo2(p, T), T, MO2)
```

```
[151]: wpT (generic function with 1 method)
```

```
[152]: wpT(300e5, 300)
```

```
[152]: 415.3954022737472
```

This value is similar to that of Weber [57].

Warm surface value

```
[153]: wpT(ptab[1], Ts)
```

```
[153]: 325.9996893882054
```

Cold surface value

```
[154]: wpT(ptab[1], Tcold)
```

```
[154]: 315.66916929963963
```

Value at depth 1000 m (index 1001 in `dvals`)

```
[155]: wpT(ptab[1001], Tcold)
```

```
[155]: 322.7128276738636
```

Value at depth 2000 m (index 2001 in `dvals`)

```
[156]: wpT(ptab[2001], Tcold)
```

```
[156]: 358.2715926515373
```

Deep water value

```
[157]: wpT(ptab[end], Tcold)
```

```
[157]: 447.86433243536436
```

Tabulate values for depth profile.

[158]:
```
wo2tab = map(p1 -> wpT(p1, Tcold), ptab);
```

[159]:
```
wo2tab[1], wo2tab[1001], wo2tab[2001], wo2tab[end]
```

[159]:
```
(315.66916929963963, 322.7128276738636, 358.2715926515373, 447.86433243536436)
```

5.1.6. Viscosity

Use the relationships from Lemmon and Jacobsen [58].

Define parameters and coefficients.

[160]:
```
pc = 5.043e6; # Pa
```

[161]:
```
bi = (0.431, -0.4623, 0.08406, 0.005341, -0.00331);
```

The following is the collision integral required for Eq. (2) in Lemmon and Jacobsen [58].

[162]:
```
Ω(Tstar) = exp(sum(bi[i+1] * (log(Tstar))^i for i in 0:4))
```

[162]:
```
Ω (generic function with 1 method)
```

[163]:
```
ϵoverk = 118.5 # K
```

[163]:
```
118.5
```

[164]:
```
Ω(300/ϵoverk)
```

[164]:
```
1.0788836619239601
```

[165]:
```
σvis = 0.3428e-9
```

[165]:
```
3.428e-10
```

The following is Eq. (2) from Lemmon and Jacobsen [58] with the constants adjusted so all values are in SI units. The resulting value is in Pa s.

Output from the following symbolic calculation has been suppressed. Remove the trailing semicolon before entering the cell to display it.

[166]:
```
η0 = 0.0266958e-24 * sqrt(1000 * M * T) / (σvis^2*Ω(T/ϵoverk));
```

The following parameters are needed for Eq. (3) in Lemmon and Jacobsen [58].

[167]:
```
Nvis = (17.67, 0.4042, 0.0001077, 0.3510, -13.67)
```

[167]:
```
(17.67, 0.4042, 0.0001077, 0.351, -13.67)
```

[168]:
```
tvis = (0.05, 0, 2.10, 0, 0.5)
```

[168]: (0.05, 0, 2.1, 0, 0.5)

[169]:
```
dvis = (1, 5, 12, 8, 1)
```

[169]: (1, 5, 12, 8, 1)

[170]:
```
lvis = (0, 0, 0, 1, 2)
```

[170]: (0, 0, 0, 1, 2)

This is Eq. (3) in Lemmon and Jacobsen [58].

Output from the following symbolic calculation has been suppressed. Remove the trailing semicolon before entering the cell to display it.

[171]:
```
ηr = 10^-6 * sum(Nvis[i] * τ^tvis[i] * δ^dvis[i] *
    exp(lvis[i] == 0 ? 0 : -δ^lvis[i]) for i in 1:5);
```

This is Eq. (1) in Lemmon and Jacobsen [58]. The viscosity of oxygen is η.

Output from the following symbolic calculation has been suppressed. Remove the trailing semicolon before entering the cell to display it.

[172]:
```
η = η0 + ηr;
```

Define a Julia function.

[173]:
```
ηjl = lambdify(η(δ => ρ/ρc, τ => Tc/T), (ρ, T, M))
```

[173]: #118 (generic function with 1 method)

Calculate some values to compare with Lemmon and Jacobsen [58].

[174]:
```
ηjl(5e3, 300, MO2) * 1e6
```

[174]: 23.757700201413066

[175]:
```
ηjl(35e3, 100, MO2) * 1e6
```

[175]: 172.13579729510082

[176]:
```
ηjl(10e3, 200, MO2) * 1e6
```

[176]: 22.44451567141834

These values are consistent with the values in Lemmon and Jacobsen [58].

Define a function of pressure and temperature.

[177]:
```
ηpT(p, T) = ηjl(ρo2(p, T), T, MO2)
```

[177]: ηpT (generic function with 1 method)

Warm surface value

[178]:
```
ηpT(ptab[1], Ts)
```

[178]: 2.027266881737361e-5

Cold surface value

[179]:
```
ηpT(ptab[1], Tcold)
```

[179]: 1.9229098582802137e-5

Value at depth 1000 m (`dvals` index 1001)

[180]:
```
ηpT(ptab[1001], Tcold)
```

[180]: 2.2091576792708548e-5

Value at depth 2000 m (`dvals` index 2001)

[181]:
```
ηpT(ptab[2001], Tcold)
```

[181]: 2.726127656589478e-5

Deep water value

[182]:
```
ηpT(ptab[end], Tcold)
```

[182]: 3.692230187855806e-5

Calculate values for the depth profile.

[183]:
```
ηo2tab = map(p1 -> ηpT(p1, Tcold), ptab);
```

[184]:
```
ηo2tab[1], ηo2tab[1001], ηo2tab[2001], ηo2tab[end]
```

[184]: (1.9229098582802137e-5, 2.2091576792708548e-5, 2.726127656589478e-5, 3.692230187855806e-5)

5.1.7. Thermal Conductivity

Use the relationships from Lemmon and Jacobsen [58].

Define the coefficients for the calculations (Table IV in Lemmon and Jacobsen [58]).

[185]:
```
Ncon = (1.036, 6.283, -4.262, 15.31, 8.898, -0.7336,
  6.728, -4.374, -0.4747)
```

[185]: (1.036, 6.283, -4.262, 15.31, 8.898, -0.7336, 6.728, -4.374, -0.4747)

The first term in the list below is for $i = 2$.

[186]:
```
tcon = (-0.9, -0.6, 0, 0, 0.3, 4.3, 0.5, 1.8)
```

[186]:
```
(-0.9, -0.6, 0, 0, 0.3, 4.3, 0.5, 1.8)
```

The first term in the list below is for $i = 4$.

[187]:
```
dcon = (1, 3, 4, 5, 7, 10)
```

[187]:
```
(1, 3, 4, 5, 7, 10)
```

The first term in the list below is for $i = 4$.

[188]:
```
lcon = (0, 0, 0, 2, 2, 2)
```

[188]:
```
(0, 0, 0, 2, 2, 2)
```

The equation below is Eq. (5) in Lemmon and Jacobsen [58] adjusted so all parameters are in SI units.

Output from the following symbolic calculation has been suppressed. Remove the trailing semicolon before entering the cell to display it.

[189]:
```
λ0 = 10^-3 * (Ncon[1] * (η0 / 10^-6) + Ncon[2] * τ^tcon[2 - 1] + Ncon[3]*τ^tcon[3 - 1]);
```

Define a Julia function for calculations.

[190]:
```
λ0jl = lambdify(λ0(τ=>Tc/T),(T, M))
```

[190]:
```
#118 (generic function with 1 method)
```

Compare symbolic and Julia function calculations.

[191]:
```
N(λ0(τ=>Tc/T, M=>MO2)(T=>100))
```

[191]:
```
0.008943340238199921795214540684620835839186806243358885993985347078141679335627
102
```

[192]:
```
λ0jl(100, MO2)
```

[192]:
```
0.008943340238199926
```

[193]:
```
N(λ0(τ=>Tc/T, M=>MO2)(T=>300))
```

[193]:
```
0.026440301365016987601270266915169642137333227786556723239898346838396525273494
83
```

[194]:
```
λ0jl(300, MO2)
```

[194]:
```
0.026440301365017002
```

These are consistent.

This is Eq. (6) in Lemmon and Jacobsen [58].

Output from the following symbolic calculation has been suppressed. Remove the trailing semicolon before entering the cell to display it.

[195]:
```
λr = 10^-3 * sum( Ncon[i] * τ^tcon[i - 1] * δ^dcon[i -
    3] * exp(lcon[i - 3] == 0 ? 0 : -δ^lcon[i - 3]) for i in
    4:9);
```

Define a Julia function for this.

[196]:
```
λrjl = lambdify(λr(δ => ρ/ρc, τ => Tc/T), (ρ, T))
```

[196]:
```
#118 (generic function with 1 method)
```

Compare symbolic and Julia function values.

[197]:
```
N(λr(δ => ρ/ρc, τ => Tc/T)(ρ => ρo2tab[end], T=>Tcold))
```

[197]:
```
0.0005631153755697235
```

[198]:
```
λrjl(ρo2tab[end], Tcold)
```

[198]:
```
0.0005631153755697235
```

These are consistent.

In order to calculate λ^c in Eq. (7) in Lemmon and Jacobsen [58], we must calculate the parameters in Eqs. (8-11).

First, we define some of the parameters in these equations.

[199]:
```
ξ0 = 0.24e-9 # m
```

[199]:
```
2.4e-10
```

[200]:
```
pc = 5.043e6 # Pa
```

[200]:
```
5.043e6
```

[201]:
```
ρc = 13.63e3 # mol/m^3
```

[201]:
```
13630.0
```

We need $\left(\frac{\partial \rho}{\partial p}\right)_T$. Use the equation of state from Eq. (A1) in Schmidt and Wagner [56] to get $\left(\frac{\partial p}{\partial \rho}\right)_T$, and invert it.

Output from the following symbolic calculation has been suppressed. Remove the trailing semicolon before entering the cell to display it.

[202]:
```
dpdρ = diff(ρ * R * T * (1 + δ * diff(αr, δ))(δ => ρ/ρc, τ => Tc/T), ρ);
```

$\left(\frac{\partial\rho}{\partial p}\right)_T$ is the reciprocal of the previous result.

Output from the following symbolic calculation has been suppressed. Remove the trailing semicolon before entering the cell to display it.

[203]:
```
dρdp = 1/dpdρ;
```

The following is Eq. (11) from Lemmon and Jacobsen [58].

Output from the following symbolic calculation has been suppressed. Remove the trailing semicolon before entering the cell to display it.

[204]:
```
χn = pc * ρ/ρc^2 * dρdp;
```

Calculate some values to test this.

[205]:
```
χn(ρ => 15654, T => 309.162)
```

[205]: 0.100290882386026

[206]:
```
N(χn(ρ => 15654, T => 309.162))
```

[206]:
```
0.10029088238602606
```

Compare the symbolic calculations above to values calculated by a Julia function.

[207]:
```
lambdify(χn(T=>309.162), (ρ,))(15654)
```

[207]:
```
0.1016422565742757
```

[208]:
```
lambdify(χn, (ρ,T))(BigFloat("15654."), BigFloat("309.162"))
```

[208]:
```
0.100290882386026026561986816030949345798372149081095358519805036184306650964384
```

[209]:
```
convert(Float64, lambdify(χn, (ρ,T))(BigFloat("15654."), BigFloat("309.162")))
```

[209]:
```
0.10029088238602603
```

There is a significant loss in precision when we go to a Julia floating point calculation from the symbolic calculation. We are going to have to use BigFloat (extended precision) numbers in Julia for `χn` and the parameters that depend on it.

Define a Julia function.

[210]:
```
χnjl = lambdify(χn, (ρ,T))
```

[210]:
```
#118 (generic function with 1 method)
```

Define some more needed parameters.

[211]:
```
Γ = 0.055
```

[211]:
```
0.055
```

[212]:
```
ν = 0.63
```

[212]:
```
0.63
```

[213]:
```
γtc = 1.2415
```

[213]:
```
1.2415
```

[214]:
```
Tref = 309.162 # K
```

[214]:
```
309.162
```

Compare some symbolic and Julia calculations to make sure everything is consistent.

[215]:
```
N(χn(ρ => ρo2(ptab[end], Tcold), T => Tcold))
```

[215]:
```
0.12908846305731383
```

[216]:
```
χnjl(BigFloat(ρo2(ptab[end], Tcold)), BigFloat(Tcold))
```

[216]:
```
0.1290884630573138707065711237495471618892319479222842391379867428471618969067207
```

[217]:
```
N(χn(ρ => ρo2(ptab[end], Tref), T => Tref))
```

[217]:
```
0.1042176516484715
```

[218]:
```
χnjl(BigFloat(ρo2(ptab[end], Tref)), BigFloat(Tref))
```

[218]:
```
0.10421765164847145960097243280890276453554969136603693055061331306039759573088811
```

The values above are consistent.

This is Eq. (10) in Lemmon and Jacobsen [58]. We define it here as a symbolic function (which produces a SymPy expression).

[219]:
```
ξ(ρ1, T1) = ξ0 * (((χn(ρ => ρ1, T => T1)) -
    (χn(ρ => ρ1, T => Tref)) * (Tref/T1)) /
    Γ)^(ν/γtc)
```

[219]:
```
ξ (generic function with 1 method)
```

Define a Julia function for this.

[220]:
```
ξjl(ρ1, T1) = ξ0 * (((χnjl(ρ1, T1)) - (χnjl(ρ1, Tref)) * (Tref/T1)) / Γ)^(ν/γtc)
```

[220]:
```
ξjl (generic function with 1 method)
```

Compare symbolic and Julia floating point calculations.

```
[221]: N(ξ(ρo2(ptab[end], Tcold), Tcold))
```

```
[221]: 1.2906193451956456e-10
```

```
[222]: N(ξ(ρo2tab[end]/MO2, Tcold))
```

```
[222]: 1.2906193451956456e-10
```

```
[223]: ξjl(ρo2tab[end]/MO2, Tcold)
```

```
[223]: 1.2906193451956505e-10
```

These are consistent.

Define some more parameters.

```
[224]: R0 = 1.01
```

```
[224]: 1.01
```

```
[225]: k = 1.380658e-23 # J/K
```

```
[225]: 1.380658e-23
```

```
[226]: qD = 0.51e-9 # m
```

```
[226]: 5.1e-10
```

The following is Eq. (8) in Lemmon and Jacobsen [58].

```
[227]: Ωn(ρ1, T1) = (2/π * ((cp - cv) / cp * atan(ξ(ρ1, T1) /
           qD) + cv / cp * ξ(ρ1, T1) / qD)(δ => ρ/ρc,
           τ => Tc/T))(ρ => ρ1, T => T1)
```

```
[227]: Ωn (generic function with 1 method)
```

Define a Julia function for this using the Julia functions for the parameters.

```
[228]: Ωnjl(ρ1, T1) = 2/π * ((cpjl(ρ1, T1) - cvjl(ρ1, T1)) /
           cpjl(ρ1, T1) * atan(ξjl(ρ1, T1) /
           qD) + cvjl(ρ1, T1) / cpjl(ρ1, T1) * ξjl(ρ1, T1) / qD)
```

```
[228]: Ωnjl (generic function with 1 method)
```

Compare symbolic and Julia calculations.

```
[229]: N(Ωn(ρo2tab[end]/MO2, Tcold))
```

```
[229]: 0.1594910300518962
```

```
[230]: Ωnjl(ρo2tab[end]/MO2, Tcold)
```

[230]: 0.15949103005189677

These are consistent.

The following is Eq. (9) in Lemmon and Jacobsen [58].

[231]:
```
Ω0n(ρ1, T1) = (2 / π * (1 - exp(-1 / ((ξ(ρ1, T1) /
    qD)^-1 + 1/3 * (ξ(ρ1, T1) / qD)^2 * (ρc /
    ρ)^2)))(δ => ρ/ρc, τ => Tc/T))(ρ => ρ1, T => T1)
```

[231]: Ω0n (generic function with 1 method)

Define a Julia function for this using the Julia functions for the parameters.

[232]:
```
Ω0njl(ρ1, T1) = 2 / π * (1 - exp(-1 / ((ξjl(ρ1, T1) /
    qD)^-1 + 1/3 * (ξjl(ρ1, T1) / qD)^2 * (ρc /
    ρ1)^2)))
```

[232]: Ω0njl (generic function with 1 method)

Compare symbolic and Julia calculations.

[233]:
```
N(Ω0n(ρo2tab[end]/MO2, Tcold))
```

[233]: 0.14182550739186106

[234]:
```
Ω0njl(ρo2tab[end]/MO2, Tcold)
```

[234]: 0.14182550739186153

These are consistent.

The following expression is Eq. (7) in Lemmon and Jacobsen [58].

[235]:
```
λc(ρ1, T1) =
    if (((χn(ρ => ρ1, T => T1)) - (χn(ρ => ρ1, T =>
        Tref)) * (Tref / T1)) / Γ) > 0
        ((ρ * cp * k * R0 * T1 / (6 * π * ξ(ρ1, T1) * η)
            * (Ωn(ρ1, T1) - Ω0n(ρ1, T1)))(δ =>
            ρ/ρc, τ => Tc/T))(ρ => ρ1, T => T1)
    else
        0
    end
```

[235]: λc (generic function with 1 method)

Define a Julia function for this using the Julia functions for the parameters.

[236]:
```
function λcjl(ρ1, T1)
    if ((χnjl(ρ1, T1) - (χnjl(ρ1, Tref)) * (Tref / T1)) / Γ) > 0
       ρ1 * cpjl(ρ1, T1) * k * R0 * T1 / (6 * π * ξjl(ρ1, T1) * ηjl(ρ1, T1, MO2)) *
```

```
            (Ωnjl(ρ1, T1) - Ω0njl(ρ1, T1))
    else
        0
    end
end
```

[236]: λcjl (generic function with 1 method)

Compare symbolic and Julia calculations.

[237]:
```
N(λc(ρo2tab[end]/MO2, Tcold)(M=>MO2))
```

[237]: 0.000530703253670222412185176359432321554010875723549134702061466374871540787203
1297

[238]:
```
λcjl(ρo2tab[end]/MO2, Tcold)
```

[238]: 0.0005307032536702239

These are consistent.

The following expression is Eq. (4) in Lemmon and Jacobsen [58].

[239]:
```
λ(ρ1, T1) = ((λ0 + λr + λc(ρ1, T1))(δ => ρ/ρc, τ => Tc/T))(ρ => ρ1, T => T1, M => MO2)
```

[239]: λ (generic function with 1 method)

Define a Julia function for this using the Julia functions for the parameters.

[240]:
```
λjl(ρ1, T1) = λ0jl(T1, MO2) + λrjl(ρ1, T1) + λcjl(ρ1, T1)
```

[240]: λjl (generic function with 1 method)

Compare symbolic and Julia calculations.

[241]:
```
N(λ(ρo2tab[end]/MO2, Tcold))
```

[241]: 0.052752955444510433116782046173814225875561385888676864015658200658423711192827
48

[242]:
```
λjl(ρo2tab[end]/MO2, Tcold)
```

[242]: 0.05275295544451044

[243]:
```
λjl(BigFloat(ρo2tab[end]/MO2), BigFloat(Tcold))
```

[243]: 0.052752955444510435097040430296883057002664877990561645179916718569370718665817
93

The values calculated above are consistent, but for more extreme parameters, we must use BigFloat (extended precision) number in the Julia calculations to get accurate results.

Compare some more symbolic and Julia values.

```
[244]: N(λ(5000, 300))
```

```
[244]: 0.032549088189674745272177722669106169298009244690226531900219840205313025037267
       71
```

```
[245]: λjl(BigFloat(5000), BigFloat(300))
```

```
[245]: 0.032549088189674749235256888276931301378206660675632141193947198318837028272821
       74
```

```
[246]: N(λ(10000, 200))
```

```
[246]: 0.034612415901784129551773222073790642974803219930639791353203080869332307171466
       17
```

```
[247]: λjl(BigFloat(10000), BigFloat(200))
```

```
[247]: 0.034612415901784122184802622661381821097952670781125652531965929828868437874540
       69
```

```
[248]: ρatm = ρo2(1.01325e5, 293.15)
```

```
[248]: 41.60020299418699
```

```
[249]: N(λ(ρatm, 293.15))
```

```
[249]: 0.025945926563591421676302437731400475809614162580656869029161562201909570993281
       45
```

Now produce some values to compare to Table V in Lemmon and Jacobsen [58].

```
[250]: N(λ(0, 300))
```

```
[250]: 0.026440301365016994529222664434908377718385018837462810152056658015696900130742
       98
```

```
[251]: λjl(BigFloat(0), BigFloat(300))
```

```
[251]: 0.026440301365016998365289370818460662631949267650210060547467109782879850939401
       15
```

```
[252]: N(λ(0, 100))
```

```
[252]: 0.008943340238199923166633864347394669058215070422100077280135001759203942532086
       278
```

```
[253]: λjl(BigFloat(0), BigFloat(100))
```

[253]:
```
0.0089433402381999253376252658041242631066359026229322406326611829724013889760833
374
```

[254]:
```
N(λ(35000, 100))
```

[254]:
```
0.14604365655620845224839611221724142935354654854099656165513500175920394253208 5
9
```

[255]:
```
λjl(BigFloat(35000), BigFloat(100))
```

[255]:
```
0.14604365655620844382351075348133597918352008095162490643425096863930782345640 8
2
```

[256]:
```
N(λ(10000, 200))
```

[256]:
```
0.034612415901784129551773222073790642974803219930639791353203080869332307171466
17
```

[257]:
```
λjl(BigFloat(10000), BigFloat(200))
```

[257]:
```
0.034612415901784122184802622661381821097952670781125652531965929828868437874540
69
```

[258]:
```
N(λ(13600, 154.6))
```

[258]:
```
0.37749328392118061417372892800888013013025378321682643212616736645738735057611 3
2
```

[259]:
```
λjl(BigFloat(13600), BigFloat(154.6))
```

[259]:
```
0.37749328392005837162574500535122417093222930290024722551244938730566568268531
9
```

These values are consistent.

Produce a Julia function of pressure and temperature.

[260]:
```
λo2pT(p1, T1) = λjl(ρo2(p1, T1), T1)
```

[260]:
```
λo2pT (generic function with 1 method)
```

Warm surface value

[261]:
```
λo2pT(BigFloat(ptab[1]), BigFloat(Ts))
```

[261]:
```
0.025945926563591425501461687379475476392469688171268793962554713231377103501947
19
```

Cold surface value

[262]:
```
λo2pT(BigFloat(ptab[1]), BigFloat(Tcold))
```

[262]:
```
0.0244704983147746610026740127521786512752634512583272040038294775239480902636207
1
```

Value at depth 1000 m (`dvals` index 1001)

[263]:
```
λo2pT(BigFloat(ptab[1001]), BigFloat(Tcold))
```

[263]:
```
0.030377400104053634025203664127849429020382439472413429588153815786206409205391
84
```

Value at depth 2000 m (`dvals` index 2001)

[264]:
```
λo2pT(BigFloat(ptab[2001]), BigFloat(Tcold))
```

[264]:
```
0.038893942408249203598977578907682186575172363603088298316963006729574767391846
74
```

Deep water value

[265]:
```
λo2pT(BigFloat(ptab[end]), BigFloat(Tcold))
```

[265]:
```
0.052752955444510427850000692062504067249589219155402483190331162606499152333250
96
```

Calculate values for the depth profile.

[266]:
```
λo2tab = map(p -> convert(Float64, λo2pT(BigFloat(p), BigFloat(Tcold))), ptab);
```

[267]:
```
λo2tab[1], λo2tab[1001], λo2tab[2001], λo2tab[end]
```

[267]:
```
(0.02447049831477466, 0.030377400104053633, 0.038893942408249206,
0.052752955444510426)
```

5.1.8. Thermal Diffusivity

Use the thermal diffusivity definition in Ainslie and Leighton [1].

[268]:
```
α(ρ1, T1) = ((λ(ρ1, T1) / (ρ1 * cp))(δ => ρ/ρc, τ => Tc/T))(ρ => ρ1, T => T1, M => MO2)
```

[268]:
```
α (generic function with 1 method)
```

Calculate a value. Recall that the `ρo2tab` array is a mass density. We must divide it by molar mass `MO2` to get molar density.

[269]:
```
N(α(ρo2tab[end]/MO2, Tcold))
```

[269]:
```
7.487219495404354540511455887588474269715990060322184300547357639720694389173966e
-08
```

Define a Julia function.

```
[270]: αjl(ρ1, T1) = λjl(ρ1, T1) / (ρ1 * cpjl(ρ1, T1))
```

```
[270]: αjl (generic function with 1 method)
```

```
[271]: αjl(BigFloat(ρo2tab[end]/MO2), BigFloat(Tcold))
```

```
[271]: 7.487219495404364329811396921172735904625033812475985565163462702756592086379079
e-08
```

```
[272]: αo2pT(p1, T1) = αjl(ρo2(p1, T1), T1)
```

```
[272]: αo2pT (generic function with 1 method)
```

```
[273]: αo2pT(BigFloat(ptab[end]), BigFloat(Tcold))
```

```
[273]: 7.487219495404364865532554975198480907690881770854359231836399180562323383626429
e-08
```

Warm surface value

```
[274]: αo2pT(BigFloat(ptab[1]), BigFloat(Ts))
```

```
[274]: 2.120985841493112653296219166693105071621395430111438146683085448897844454307706
e-05
```

Cold surface value

```
[275]: αo2pT(BigFloat(ptab[1]), BigFloat(Tcold))
```

```
[275]: 1.877973098323995278124008176810104328920435150793272695302657778424528852338461
e-05
```

Value at depth 1000 m (`dvals` index 1001)

```
[276]: αo2pT(BigFloat(ptab[1001]), BigFloat(Tcold))
```

```
[276]: 1.719742443384715854418450771641508186342003600288733110583327019015409320791956
e-07
```

Value at depth 2000 m (`dvals` index 2001)

```
[277]: αo2pT(BigFloat(ptab[2001]), BigFloat(Tcold))
```

```
[277]: 9.264192554939795735130225941054086882870839425383077380518022707172419462590197
e-08
```

Deep water value

```
[278]: αo2pT(BigFloat(ptab[end]), BigFloat(Tcold))
```

[278]: 7.487219495404364865532554975198480907690881770854359231836399180562323383626429
e-08

```
[279]: αo2tab = map(p -> convert(Float64, αo2pT(BigFloat(p), BigFloat(Tcold))),
           ptab);
```

```
[280]: αo2tab[1], αo2tab[1001], αo2tab[2001], αo2tab[end]
```

[280]: (1.8779730983239953e-5, 1.7197424433847157e-7, 9.264192554939795e-8,
7.487219495404365e-8)

```
[281]: valso2.thermal_diffusivity = αo2tab;
```

5.1.9. Export Oxygen Parameters

We have all the needed oxygen parameters in `valso2`. Export them to a CSV file.

Here are the column names.

```
[282]: names(valso2)
```

```
[282]: 8-element Vector{String}:
 "depth"
 "pressure"
 "water_density"
 "water_dyn_viscosity"
 "water_surface_tension"
 "water_sound_speed"
 "gamma"
 "thermal_diffusivity"
```

Reorder the dataframe columns to match the order that the acoustic calculations require.

```
[283]: select!(valso2, ["depth", "water_density", "pressure", "water_dyn_viscosity",
               "water_surface_tension", "water_sound_speed",
               "thermal_diffusivity", "gamma"]);
```

```
[284]: CSV.write("DeepWaterPropertiesO2.csv", valso2)
```

[284]: "DeepWaterPropertiesO2.csv"

5.2. Nitrogen

We calculate nitrogen densities, heat capacities, and sound speeds using the relationships from Span *et al.* [55]. We calculate thermal conductivities using the relationships in Lemmon and Jacobsen [58]. We calculate thermal diffusivities using the relationship in Ainslie and Leighton [1].

Define some gas parameters from Span *et al.* [55].

Make sure `R` is defined.

```
[285]: R = 8.314510; # J mol^-1 K^-1, universal gas constant
```

```
[286]: MN2 = 28.01348/1000; # kg, nitrogen molar mass
```

Define variables used in symbolic calculations.

```
[287]: @vars δ τ ρ T M
```

```
[287]: (δ, τ, ρ, T, M)
```

Define the coefficients for Eq. (53) in Span *et al.* [55].

```
[288]: a = (2.5, -12.76952708, -0.00784163, -1.934819e-4, -1.247742e-5,
           6.678326e-8, 1.012941, 26.65788)
```

```
[288]: (2.5, -12.76952708, -0.00784163, -0.0001934819, -1.247742e-5, 6.678326e-8,
1.012941, 26.65788)
```

This is Eq. (53) in Span *et al.* [55].

Output from the following symbolic calculation has been suppressed. Remove the trailing semicolon before entering the cell to display it.

```
[289]: α0 =  log(δ) + a[1] * log(τ) + a[2] + a[3] * τ +
         a[4] * τ^-1 + a[5] * τ^-2 + a[6] * τ^-3 +
         a[7] * log(1 - exp(-a[8] * τ));
```

The following parameters are from Table 17 in Span *et al.* [55].

```
[290]: nk = (0.924803575275, -0.492448489428, 0.661883336938,
           -0.192902649201e1, -0.622469309629e-1, 0.349943957581,
           0.564857472498, -0.161720005987e1, -0.481395031883,
           0.421150636384, -0.161962230825e-1, 0.172100994165,
           0.735448924933e-2, 0.168077305479e-1, -0.107626664179e-2,
           -0.137318088513e-1, 0.635466899859e-3, 0.304432279419e-2,
           -0.435762336045e-1, -0.723174889316e-1, 0.389644315272e-1,
           -0.212201363910e-1, 0.408822981509e-2, -0.551990017948e-4,
           -0.462016716479e-1, -0.300311716011e-2, 0.368825891208e-1,
           -0.255856846220e-2, 0.896915264558e-2, -0.441513370350e-2,
           0.133722924858e-2, 0.264832491957e-3, 0.196688194015e2,
           -0.209115600730e2, 0.167788306989e-1, 0.262767566274e4)
```

```
[290]: (0.924803575275, -0.492448489428, 0.661883336938, -1.92902649201,
-0.0622469309629, 0.349943957581, 0.564857472498, -1.61720005987,
-0.481395031883, 0.421150636384, -0.0161962230825, 0.172100994165,
0.00735448924933, 0.0168077305479, -0.00107626664179, -0.0137318088513,
0.000635466899859, 0.00304432279419, -0.0435762336045, -0.0723174889316,
0.0389644315272, -0.021220136391, 0.00408822981509, -5.51990017948e-5,
-0.0462016716479, -0.00300311716011, 0.0368825891208, -0.0025585684622,
```

```
0.00896915264558, -0.0044151337035, 0.00133722924858, 0.000264832491957,
19.6688194015, -20.911560073, 0.0167788306989, 2627.67566274)
```

```
[291]: ik = (1, 1, 2, 2, 3, 3, 1, 1, 1, 3, 3, 4, 6, 6, 7, 7, 8, 8, 1, 2, 3,
           4, 5, 8, 4, 5, 5, 8, 3, 5, 6, 9, 1, 1, 3, 2)
```

```
[291]: (1, 1, 2, 2, 3, 3, 1, 1, 1, 3, 3, 4, 6, 6, 7, 7, 8, 8, 1, 2, 3, 4, 5, 8, 4, 5,
5, 8, 3, 5, 6, 9, 1, 1, 3, 2)
```

```
[292]: jk = (0.25, 0.875, 0.5, 0.875, 0.375, 0.75, 0.5, 0.75, 2, 1.25, 3.5,
           1, 0.5, 3, 0, 2.75, 0.75, 2.5, 4, 6, 6, 3, 3, 6, 16, 11, 15, 12,
           12, 7, 4, 16, 0, 1, 2, 3)
```

```
[292]: (0.25, 0.875, 0.5, 0.875, 0.375, 0.75, 0.5, 0.75, 2, 1.25, 3.5, 1, 0.5, 3, 0,
2.75, 0.75, 2.5, 4, 6, 6, 3, 3, 6, 16, 11, 15, 12, 12, 7, 4, 16, 0, 1, 2, 3)
```

```
[293]: lk = (0, 0, 0, 0, 0, 0, 1, 1, 1, 1, 1, 1, 1, 1, 1, 1, 1, 1, 2, 2, 2,
           2, 2, 2, 3, 3, 3, 3, 4, 4, 4, 4, 2, 2, 2, 2)
```

```
[293]: (0, 0, 0, 0, 0, 0, 1, 1, 1, 1, 1, 1, 1, 1, 1, 1, 1, 1, 2, 2, 2, 2, 2, 2, 3, 3,
3, 3, 4, 4, 4, 4, 2, 2, 2, 2)
```

The following parameters are from Table 18 in Span *et al.* [55]. The first ϕ, β, and γ indices in the equation are each 33. these are the first terms below.

```
[294]: ϕk = (20, 20, 15, 25)
```

```
[294]: (20, 20, 15, 25)
```

```
[295]: βk = (325, 325, 300, 275)
```

```
[295]: (325, 325, 300, 275)
```

```
[296]: γk = (1.16, 1.16, 1.13, 1.25)
```

```
[296]: (1.16, 1.16, 1.13, 1.25)
```

This is Eq (55) from Span *et al.* [55].

Output from the following symbolic calculation has been suppressed. Remove the trailing semicolon before entering the cell to display it.

```
[297]: αr = sum(nk[k] * δ^ik[k] * τ^jk[k] for k in 1:6) +
           sum(nk[k] * δ^ik[k] * τ^jk[k] * exp(-δ^lk[k])
           for k in 7:32) + sum(nk[k] * δ^ik[k] * τ^jk[k] *
           exp(-ϕk[k - 32] * (δ - 1)^2 - βk[k - 32] * (τ -
           γk[k - 32])^2) for k in 33:36);
```

Define the critical temperature and density.

[298]:
```
Tc = 126.192; # K
```

[299]:
```
ρc = 11.1839e3; # mol/m^3
```

5.2.1. Density

This is Eq. (56) from Span *et al.* [55].

Output from the following symbolic calculation has been suppressed. Remove the trailing semicolon before entering the cell to display it.

[300]:
```
ρeq = Eq(p, ρ * R * T * (1 + δ * diff(αr, δ))(δ => ρ/ρc, τ => Tc/T));
```

Create a Julia function that we can use to find roots numerically.

[301]:
```
ρeqjl = lambdify(lhs(ρeq) - rhs(ρeq), (p, T, ρ))
```

[301]:
```
#118 (generic function with 1 method)
```

Now create a function that numerically finds a root for the density at a given value of pressure and temperature.

[302]:
```
ρn2(p, T, ρ0=24000.0) = find_zero(ρ1 -> ρeqjl(p, T, ρ1), ρ0)
```

[302]:
```
ρn2 (generic function with 2 methods)
```

Now compare to densities from Span *et al.* [55] N2 data. Find the density at $p = 75$ Mbar (750×10^5 Pa) and $T = 270$ K.

[303]:
```
ρn2(750e5, 270)
```

[303]:
```
19395.841644638156
```

Now find the density at $p = 75$ Mbar (750×10^5 Pa) and $T = 300$ K.

[304]:
```
ρn2(750e5, 300)
```

[304]:
```
18053.5804495223
```

These values are consistent.

Warm surface values

[305]:
```
ρn2(ptab[1], Ts)
```

[305]:
```
41.58105951222148
```

[306]:
```
ρn2(ptab[1], Ts) * MN2
```

[306]:
```
1.1648301790244262
```

Cold surface values

[307]:
```
ρn2(ptab[1], Tcold)
```

[307]: 44.39057228867911

[308]:
```
ρn2(ptab[1], Tcold) * MN2
```

[308]: 1.2435344089974665

Values at depth 1000 m (`dvals` index 1001)

[309]:
```
ρn2(ptab[1001], Tcold)
```

[309]: 4524.56749585226

[310]:
```
ρn2(ptab[1001], Tcold) * MN2
```

[310]: 126.74888105370738

Values at depth 2000 m (`dvals` index 2001)

[311]:
```
ρn2(ptab[2001], Tcold)
```

[311]: 8578.084713569095

[312]:
```
ρn2(ptab[2001], Tcold) * MN2
```

[312]: 240.30200456187356

Deep water values

[313]:
```
ρn2(ptab[end], Tcold)
```

[313]: 13047.278015394078

[314]:
```
ρn2(ptab[end], Tcold) * MN2
```

[314]: 365.4996617386817

Now tabulate mass density values for the depth profile by multiplying each molar density by the molar mass.

[315]:
```
ρn2tab = map(p1 -> ρn2(p1, Tcold) * MN2, ptab);
```

[316]:
```
ρn2tab[1], ρn2tab[1001], ρn2tab[2001], ρn2tab[end]
```

[316]: (1.2435344089974665, 126.74888105370738, 240.30200456187356, 365.4996617386817)

5.2.2. Isochoric Heat Capacity c_v

The following is Eq. (62) from Span *et al.* [55].

Output from the following symbolic calculation has been suppressed. Remove the trailing semicolon before entering the cell to display it.

[317]:
```
cv = -τ^2 * (diff(α0, τ, 2) + diff(αr, τ, 2)) * R;
```

Define a Julia function for calculations.

[318]:
```
cvjl = lambdify(cv(δ => ρ/ρc, τ => Tc/T),(ρ, T))
```

[318]:
```
#118 (generic function with 1 method)
```

Define a function of pressure and temperature.

[319]:
```
cvpTn2(p, T) = cvjl(ρn2(p, T), T)
```

[319]:
```
cvpTn2 (generic function with 1 method)
```

Calculate some values and compare with Span *et al.* [55].

[320]:
```
cvpTn2(0.2e6, 290)
```

[320]:
```
20.82243462328305
```

[321]:
```
cvpTn2(75e6, 270)
```

[321]:
```
23.810136385096367
```

These values are consistent.

Warm surface value

[322]:
```
cvpTn2(ptab[1], Ts)
```

[322]:
```
20.81602794850762
```

Cold surface value

[323]:
```
cvpTn2(ptab[1], Tcold)
```

[323]:
```
20.81106217231203
```

Value at depth 1000 m (`dvals` index 1001)

[324]:
```
cvpTn2(ptab[1001], Tcold)
```

[324]:
```
21.575464252247457
```

Value at depth 2000 m (`dvals` index 2001)

[325]:
```
cvpTn2(ptab[2001], Tcold)
```

[325]: 22.104361935551182

Deep water value

[326]:
```
cvpTn2(ptab[end], Tcold)
```

[326]: 22.655294332204793

5.2.3. Isobaric Heat Capacity c_p

The following is Eq. (63) from Span *et al.* [55].

Output from the following symbolic calculation has been suppressed. Remove the trailing semicolon before entering the cell to display it.

[327]:
```
cp = (cv + (1 + δ * diff(αr, δ) - δ * τ *
    diff(αr, δ, τ))^2 / (1 + 2 * δ * diff(αr, δ) +
    δ^2 * diff(αr, δ, δ)) * R);
```

Define a Julia function to calculate values.

[328]:
```
cpjl = lambdify(cp(δ => ρ/ρc, τ => Tc/T),(ρ, T))
```

[328]: #118 (generic function with 1 method)

Define another Julia function that uses an input pressure and temperature to calculate the needed density value.

[329]:
```
cppTn2(p, T) = cpjl(ρn2(p, T), T)
```

[329]: cppTn2 (generic function with 1 method)

Calculate some values.

[330]:
```
cppTn2(0.2e6, 290)
```

[330]: 29.21999613937129

[331]:
```
cppTn2(75e6, 270)
```

[331]: 39.36103974887872

These values are consistent with Span *et al.* [55].

Warm surface value

[332]:
```
cppTn2(ptab[1], Ts)
```

[332]: 29.171517219967

Cold surface value

[333]:
```
cppTn2(ptab[1], Tcold)
```

[333]: 29.173417101590708

Value at depth 1000 m (`dvals` index 1001)

[334]:
```
cppTn2(ptab[1001], Tcold)
```

[334]: 34.778572361839494

Value at depth 2000 m (`dvals` index 2001)

[335]:
```
cppTn2(ptab[2001], Tcold)
```

[335]: 38.42633485169769

Deep water value

[336]:
```
cppTn2(ptab[end], Tcold)
```

[336]: 39.87145571233225

5.2.4. Ratio of Specific Heats γ

Use the values of the two specific heats to calculate $\gamma = c_p/c_v$.

[337]:
```
γn2(p, T) = cppTn2(p, T) / cvpTn2(p, T)
```

[337]: γn2 (generic function with 1 method)

Warm surface value

[338]:
```
γn2(ptab[1], Ts)
```

[338]: 1.4013969087728102

Cold surface value

[339]:
```
γn2(ptab[1], Tcold)
```

[339]: 1.4018225912757267

Value at depth 1000 m (`dvals` index 1001)

[340]:
```
γn2(ptab[1001], Tcold)
```

[340]: 1.6119501279429806

Value at depth 2000 m (`dvals` index 2001)

[341]:
```
γn2(ptab[2001], Tcold)
```

[341]: 1.7384050697204398

Deep water value

[342]:
```
γn2(ptab[end], Tcold)
```

[342]: 1.7599177979186287

Calculate values for the depth profile.

[343]:
```
γn2tab = map(p1 -> γn2(p1, Tcold), ptab);
```

[344]:
```
γn2tab[1], γn2tab[1001], γn2tab[2001], γn2tab[end]
```

[344]: (1.4018225912757267, 1.6119501279429806, 1.7384050697204398, 1.7599177979186287)

Add these values to the dataframe.

[345]:
```
valsn2.gamma = γn2tab;
```

5.2.5. Sound Speed

The following expression is Eq. (64) from Span *et al.* [55].

Output from the following symbolic calculation has been suppressed. Remove the trailing semicolon before entering the cell to display it.

[346]:
```
w = sqrt( R * T / M * (1 + 2 * δ * diff(αr, δ) + δ^2 * diff(αr, δ, δ) -
    (1 + δ * diff(αr, δ) - δ * τ * diff(αr, δ, τ))^2 / (τ^2 *
    (diff(α0, τ, τ) + diff(αr, τ, τ)))));
```

Define a Julia function.

[347]:
```
wjl = lambdify(w(δ => ρ/ρc, τ => Tc/T),(ρ, T, M))
```

[347]: #118 (generic function with 1 method)

Define a function of pressure and temperature as before.

[348]:
```
wpT(p, T) = wjl(ρn2(p, T), T, MN2)
```

[348]: wpT (generic function with 1 method)

Check values for 0.2 MPa

[349]:
```
wpT(0.2e6, 290)
```

[349]: 347.3589765325666

Check values for 75 MPa and 270 K.

[350]:
```
wpT(75e6, 270)
```

[350]:
```
749.3016933093184
```

These are consistent with Span *et al.* [55].

Warm surface value

[351]:
```
wpT(ps, Ts)
```

[351]:
```
349.1044228816854
```

Cold surface value

[352]:
```
wpT(ps, Tcold)
```

[352]:
```
337.89465634739565
```

Value at depth 1000 m (`dvals` index 1001)

[353]:
```
wpT(ptab[1001], Tcold)
```

[353]:
```
363.76133310795603
```

Value at depth 2000 m (`dvals` index 2001)

[354]:
```
wpT(ptab[2001], Tcold)
```

[354]:
```
416.69020841364494
```

Deep water value

[355]:
```
wpT(ptab[end], Tcold)
```

[355]:
```
517.0273889822301
```

Tabulate values for depth profile.

[356]:
```
wn2tab = map(p1 -> wpT(p1, Tcold), ptab);
```

[357]:
```
wn2tab[1], wn2tab[1001], wn2tab[2001], wn2tab[end]
```

[357]:
```
(337.89465634739565, 363.76133310795603, 416.69020841364494, 517.0273889822301)
```

5.2.6. Viscosity

Use the relationships from Lemmon and Jacobsen [58].

[358]:
```
pc = 3.3958e6; # Pa
```

The following parameters were defined in the oxygen section above. We reuse them here.

[359]:
```
bi
```

[359]: (0.431, -0.4623, 0.08406, 0.005341, -0.00331)

[360]:
```
Ω(T)
```

[360]: $\frac{1.53879554995687e^{-0.00331\log{(T)}^4+0.005341\log{(T)}^3+0.08406\log{(T)}^2}}{T^{0.4623}}$

Define some other needed parameters.

[361]:
```
ϵoverk = 98.94 # K
```

[361]: 98.94

[362]:
```
Ω(300/ϵoverk)
```

[362]: 1.0241852717016806

[363]:
```
σvis = 0.3656e-9
```

[363]: 3.656e-10

The following is Eq. (2) from Lemmon and Jacobsen [58] with the constants adjusted so all values are in SI units. The resulting value is in Pa s.

Output from the following symbolic calculation has been suppressed. Remove the trailing semicolon before entering the cell to display it.

[364]:
```
η0 = 0.0266958e-24 * sqrt(1000 * M * T) / (σvis^2 * Ω(T/ϵoverk));
```

Calculate a value for $T = 300$ K.

[365]:
```
N(η0(T=>300, M=>MN2))
```

[365]: 1.787706414600966559101560198695408335590729400220960251909887121698465030839747
e-05

These are the parameters in Table III in Lemmon and Jacobsen [58].

[366]:
```
Nvis = (10.72, 0.03989, 0.001208, -7.402, 4.620)
```

[366]: (10.72, 0.03989, 0.001208, -7.402, 4.62)

[367]:
```
tvis = (0.1, 0.25, 3.2, 0.9, 0.3)
```

[367]: (0.1, 0.25, 3.2, 0.9, 0.3)

[368]:
```
dvis = (2, 10, 12, 2, 1)
```

[368]: (2, 10, 12, 2, 1)

[369]:
```
lvis = (0, 1, 1, 2, 3)
```

[369]:
```
(0, 1, 1, 2, 3)
```

This is Eq. (3) in Lemmon and Jacobsen [58].

Output from the following symbolic calculation has been suppressed. Remove the trailing semicolon before entering the cell to display it.

[370]:
```
ηr = 10^-6 * sum(Nvis[i] * τ^tvis[i] * δ^dvis[i] *
    exp(lvis[i] == 0 ? 0 : -δ^lvis[i]) for i in 1:5);
```

This is Eq. (1) in Lemmon and Jacobsen [58]. The viscosity of oxygen is η.

Output from the following symbolic calculation has been suppressed. Remove the trailing semicolon before entering the cell to display it.

[371]:
```
η = η0 + ηr;
```

Define a Julia function.

[372]:
```
ηjl = lambdify(η(δ => ρ/ρc, τ => Tc/T), (ρ, T, M))
```

[372]:
```
#118 (generic function with 1 method)
```

Calculate some values to compare with Lemmon and Jacobsen [58].

[373]:
```
1e6 * ηjl(5e3, 300, MN2)
```

[373]:
```
20.743041742625184
```

[374]:
```
1e6 * ηjl(25e3, 100, MN2)
```

[374]:
```
79.7417506134048
```

[375]:
```
1e6 * ηjl(10e3, 200, MN2)
```

[375]:
```
21.081044490030866
```

These values are consistent with the values in Lemmon and Jacobsen [58].

Define a function of pressure and temperature.

[376]:
```
ηpT(p, T) = ηjl(ρn2(p, T), T, MN2)
```

[376]:
```
ηpT (generic function with 1 method)
```

Warm surface value

[377]:
```
ηpT(ps, Ts)
```

[377]:
```
1.7572933092983353e-5
```

Cold surface value

[378]:
```
ηpT(ps, Tcold)
```

[378]:
```
1.6700484916609287e-5
```

Value at depth 1000 m (`dvals` index 1001)

[379]:
```
ηpT(ptab[1001], Tcold)
```

[379]:
```
1.9183615129628023e-5
```

Value at depth 2000 m (`dvals` index 2001)

[380]:
```
ηpT(ptab[2001], Tcold)
```

[380]:
```
2.31078724711162e-5
```

Deep water value

[381]:
```
ηpT(ptab[end], Tcold)
```

[381]:
```
2.982149636014933e-5
```

Calculate values for the depth profile.

[382]:
```
ηn2tab = map(p1 -> ηpT(p1, Tcold), ptab);
```

[383]:
```
ηn2tab[1], ηn2tab[1001], ηn2tab[2001], ηn2tab[end]
```

[383]:
```
(1.6700484916609287e-5, 1.9183615129628023e-5, 2.31078724711162e-5,
2.982149636014933e-5)
```

5.2.7. Thermal Conductivity

Use the relationships from Lemmon and Jacobsen [58].

Define the coefficients in Table IV in Lemmon and Jacobsen [58].

[384]:
```
Ncon = (1.511, 2.117, -3.332, 8.862, 31.11, -73.13, 20.03, -0.7096, 0.2672)
```

[384]:
```
(1.511, 2.117, -3.332, 8.862, 31.11, -73.13, 20.03, -0.7096, 0.2672)
```

The first term in the list below is for $i = 2$.

[385]:
```
tcon = (-1.0, -0.7, 0, 0.03, 0.2, 0.8, 0.6, 1.9)
```

[385]:
```
(-1.0, -0.7, 0, 0.03, 0.2, 0.8, 0.6, 1.9)
```

The first term in the list below is for $i = 4$.

[386]:
```
dcon = (1, 2, 3, 4, 8, 10)
```

[386]: (1, 2, 3, 4, 8, 10)

The first term in the list below is for $i = 4$.

```
[387]: lcon = (0, 0, 1, 2, 2, 2)
```

[387]: (0, 0, 1, 2, 2, 2)

The equation below is Eq (5) in Lemmon and Jacobsen [58] adjusted so all parameters are in SI units.

Output from the following symbolic calculation has been suppressed. Remove the trailing semicolon before entering the cell to display it.

```
[388]: λ0 = 10^-3 * (Ncon[1] * (η0 / 10^-6) + Ncon[2] * τ^tcon[2 - 1] + Ncon[3] *
           τ^tcon[3 - 1]);
```

Define a Julia function for calculations.

```
[389]: λ0jl = lambdify(λ0(τ=>Tc/T),(T, M))
```

[389]: #118 (generic function with 1 method)

Compare symbolic and Julia function calculations.

```
[390]: N(λ0(τ=>Tc/T, M=>MN2)(T=>100))
```

[390]: 0.00927749392871776713910514104183435305005724945855386717792512728375971553062074

```
[391]: λ0jl(100, MN2)
```

[391]: 0.00927749392871777

```
[392]: N(λ0(τ=>Tc/T, M=>MN2)(T=>300))
```

[392]: 0.02593608667121783418557850017966115010524922926872603486862866737795929613647187

```
[393]: λ0jl(300, MN2)
```

[393]: 0.025936086671217842

These values are consistent.

This is Eq. (6) in Lemmon and Jacobsen [58].

Output from the following symbolic calculation has been suppressed. Remove the trailing semicolon before entering the cell to display it.

```
[394]: λr = 10^-3 * sum( Ncon[i] * τ^tcon[i - 1] * δ^dcon[i -
           3] * exp(lcon[i - 3] == 0 ? 0 : -δ^lcon[i - 3]) for i in
```

```
    4:9);
```

Define a Julia function for this.

[395]:
```
λrjl = lambdify(λr(δ => ρ/ρc, τ => Tc/T), (ρ, T))
```

[395]:
```
#118 (generic function with 1 method)
```

Compare symbolic and Julia function values.

[396]:
```
N(λr(δ => ρ/ρc, τ => Tc/T)(ρ => ρn2tab[end]/MN2, T=>Tcold))
```

[396]:
```
0.025539505273607738
```

[397]:
```
λrjl(ρn2tab[end]/MN2, Tcold)
```

[397]:
```
0.025539505273607738
```

In order to calculate λ^c in Eq. (7) in Lemmon and Jacobsen [58], we must calculate the parameters in Eqs. (8-11).

First, we define some of the parameters in these equations.

[398]:
```
ξ0 = 0.17e-9; # m
```

[399]:
```
pc = 3.3958e6; # Pa
```

[400]:
```
ρc = 11.1839e3; # mol/m^3
```

We need $\left(\frac{\partial \rho}{\partial p}\right)_T$. Use the equation of state from Eq. (56) from Lemmon and Jacobsen [58] to get $\left(\frac{\partial p}{\partial \rho}\right)_T$, and invert it.

Output from the following symbolic calculation has been suppressed. Remove the trailing semicolon before entering the cell to display it.

[401]:
```
dpdρ = diff(ρ * R * T * (1 + δ * diff(αr, δ))(δ => ρ/ρc, τ => Tc/T), ρ);
```

$\left(\frac{\partial \rho}{\partial p}\right)_T$ is the reciprocal of the previous result.

Output from the following symbolic calculation has been suppressed. Remove the trailing semicolon before entering the cell to display it.

[402]:
```
dρdp = 1/dpdρ;
```

The following is Eq. (11) from Lemmon and Jacobsen [58].

Output from the following symbolic calculation has been suppressed. Remove the trailing semicolon before entering the cell to display it.

[403]:
```
χn = pc * ρ/ρc^2 * dρdp;
```

Calculate some values to test this.

[404]:
```
χn(ρ => 15654, T => 309.162)
```

[404]:

0.0602175741298228

[405]:
```
N(χn(ρ => 15654, T => 309.162))
```

[405]: 0.06021757412982283

[406]:
```
lambdify(χn(T=>309.162), (ρ,))(15654)
```

[406]: 0.05678388205384859

[407]:
```
lambdify(χn, (ρ,T))(BigFloat("15654."), BigFloat("309.162"))
```

[407]: 0.060217574129822887007799763061890037026730709124110658707483880417092010663093
67

[408]:
```
convert(Float64, lambdify(χn, (ρ,T))(BigFloat("15654."), BigFloat("309.162")))
```

[408]: 0.060217574129822884

There is a significant loss in precision when we go to a floating point calculation from the symbolic calculation. We are going to have to use BigFloat numbers for `χn` and the parameters that depend on it.

Define a Julia function.

[409]:
```
χnjl = lambdify(χn, (ρ,T))
```

[409]: #118 (generic function with 1 method)

Define some more needed parameters.

[410]:
```
Γ = 0.055
```

[410]: 0.055

[411]:
```
ν = 0.63
```

[411]: 0.63

[412]:
```
γtc = 1.2415
```

[412]: 1.2415

[413]:
```
Tref = 252.384 # K
```

[413]: 252.384

Compare some symbolic and Julia calculations to make sure everything is consistent.

[414]:
```
N(χn(ρ => ρn2(ptab[end], Tcold), T => Tcold))
```

[414]: 0.08324797739948357

[415]:
```
χnjl(BigFloat(ρn2(ptab[end], Ts)), BigFloat(Ts))
```

[415]: 0.076145000193314448988648918807544146655676569118794179944088188349044090213834
84

[416]:
```
χnjl(BigFloat(ρn2(ptab[end], Tref)), BigFloat(Tref))
```

[416]: 0.091986841582792361048359538515230457305691383217881175805080217419166680283764
03

[417]:
```
N(χn(ρ => ρn2(ptab[end], Tref), T => Tref))
```

[417]: 0.09198684158279254

[418]:
```
χnjl(BigFloat(ρn2(ptab[end], Tref)), BigFloat(Tref))
```

[418]: 0.091986841582792361048359538515230457305691383217881175805080217419166680283764
03

The values above are consistent.

This is Eq. (10) in Lemmon and Jacobsen [58]. We define it here as a symbolic function (which produces a SymPy expression).

[419]:
```
ξ(ρ1, T1) = ξ0 * (((χn(ρ => ρ1, T => T1)) -
    (χn(ρ => ρ1, T => Tref)) * (Tref/T1)) /
    Γ)^(ν/γtc)
```

[419]: ξ (generic function with 1 method)

Define a Julia function for this.

[420]:
```
ξjl(ρ1, T1) = ξ0 * (((χnjl(ρ1, T1)) - (χnjl(ρ1, Tref)) * (Tref/T1)) / Γ)^(ν/γtc)
```

[420]: ξjl (generic function with 1 method)

Compare symbolic and Julia floating point calculations.

[421]:
```
N(ξ(ρn2tab[end]/MN2, Tcold))
```

[421]: -1.3442826598788053e-12 + 5.7420423384909375e-11im

[422]:
```
ξjl(BigFloat(ρn2tab[end]+ 0im), BigFloat(Tcold+0im))
```

[422]: NaN

The results above are correct. The temperatures given (and the temperatures in all the underwater environments in the paper) are greater than the critical temperature; therefore ξ does not contribute to the thermal conductivity through the λ_c term defined in Eq. (7) in Lemmon and Jacobsen [58].

Define some more paraeters.

[423]:
```
R0 = 1.01;
```

[424]:
```
k = 1.380658e-23; # J/K
```

[425]:
```
qD = 0.40e-9; # m
```

The following is Eq. (8) in Lemmon and Jacobsen [58].

[426]:
```
Ωn(ρ1, T1) = (2/π * ((cp - cv) / cp * atan(ξ(ρ1, T1) /
    qD) + cv / cp * ξ(ρ1, T1) / qD)(δ => ρ/ρc,
    τ => Tc/T))(ρ => ρ1, T => T1)
```

[426]:
```
Ωn (generic function with 1 method)
```

Define a Julia function for this using the Julia functions for the parameters.

[427]:
```
Ωnjl(ρ1, T1) = 2/π * ((cpjl(ρ1, T1) - cvjl(ρ1, T1)) /
    cpjl(ρ1, T1) * atan(ξjl(ρ1, T1) /
    qD) + cvjl(ρ1, T1) / cpjl(ρ1, T1) * ξjl(ρ1, T1) / qD)
```

[427]:
```
Ωnjl (generic function with 1 method)
```

Compare symbolic and Julia calculations.

[428]:
```
N(Ωn(ρn2tab[end], Tcold))
```

[428]:
```
-0.00010979933356491272 + 0.004689978682126876im
```

[429]:
```
Ωnjl(ρn2tab[end], Tcold + 0im)
```

[429]:
```
-0.00010979933356491013 + 0.004689978682126766im
```

The following is Eq. (9) in Lemmon and Jacobsen [58].

[430]:
```
Ω0n(ρ1, T1) = (2 / π * (1 - exp(-1 / ((ξ(ρ1, T1) /
    qD)^-1 + 1/3 * (ξ(ρ1, T1) / qD)^2 * (ρc /
    ρ)^2)))(δ => ρ/ρc, τ => Tc/T))(ρ => ρ1, T => T1)
```

[430]:
```
Ω0n (generic function with 1 method)
```

Define a Julia function for this using the Julia functions for the parameters.

[431]:
```
Ω0njl(ρ1, T1) = 2 / π * (1 - exp(-1 / ((ξjl(ρ1, T1) /
    qD)^-1 + 1/3 * (ξjl(ρ1, T1) / qD)^2 * (ρc /
    ρ1)^2)))
```

[431]: Ω0njl (generic function with 1 method)

Compare symbolic and Julia calculations.

```
[432]: N(Ω0n(ρn2tab[end], Tcold))
```

[432]: -9.311257188915345e-5 + 0.004690669700665422im

```
[433]: Ω0njl(ρn2tab[end], Tcold + 0im)
```

[433]: -9.311257188919424e-5 + 0.004690669700665313im

These are consistent.

The following expression is Eq. (7) in Lemmon and Jacobsen [58].

```
[434]: λc(ρ1, T1) =
    if (((χn(ρ => ρ1, T => T1)) - (χn(ρ => ρ1, T =>
        Tref)) * (Tref / T1)) / Γ) > 0
        ((ρ * cp * k * R0 * T1 / (6 * π * ξ(ρ1, T1) * η)
            * (Ωn(ρ1, T1) - Ω0n(ρ1, T1)))(δ =>
            ρ/ρc, τ => Tc/T))(ρ => ρ1, T => T1)
    else
        0
    end
```

[434]: λc (generic function with 1 method)

Define a Julia function for this using the Julia functions for the parameters.

```
[435]: function λcjl(ρ1, T1)
    if ((χnjl(ρ1, T1) - (χnjl(ρ1, Tref)) * (Tref / T1)) / Γ) > 0
       ρ1 * cpjl(ρ1, T1) * k * R0 * T1 / (6 * π * ξjl(ρ1, T1) * ηjl(ρ1, T1, MN2)) *
            (Ωnjl(ρ1, T1) - Ω0njl(ρ1, T1))
    else
        0
    end
end
```

[435]: λcjl (generic function with 1 method)

Compare symbolic and Julia calculations.

```
[436]: λc(ρn2tab[end]/MN2, Tcold)
```

[436]: 0

```
[437]: λcjl(ρn2tab[end]/MN2, Tcold)
```

[437]: 0

These are consistent.

The following expression is Eq. (4) in Lemmon and Jacobsen [58].

[438]:
```
λ(ρ1, T1) = ((λ0 + λr + λc(ρ1, T1))(δ => ρ/ρc, τ => Tc/T))(ρ => ρ1, T => T1, M => MN2)
```

[438]:
```
λ (generic function with 1 method)
```

Define a Julia function for this using the Julia functions for the parameters.

[439]:
```
λjl(ρ1, T1) = λ0jl(T1, MN2) + λrjl(ρ1, T1) + λcjl(ρ1, T1)
```

[439]:
```
λjl (generic function with 1 method)
```

Compare symbolic and Julia calculations.

[440]:
```
N(λ(ρn2(ptab[end], Tcold), Tcold))
```

[440]:
```
0.049616519403474207031096168609947088052581303858746925646843920896990484841775
91
```

[441]:
```
λjl(BigFloat(ρn2(ptab[end], Tcold)), BigFloat(Tcold))
```

[441]:
```
0.049616519403474200897719979075481186647608518538320295905639954780018200954626
53
```

Compare some more symbolic and Julia values.

[442]:
```
N(λ(5000, 300))
```

[442]:
```
0.032769431881436595764435386959552229104887888057690054045065901402031279198819
55
```

[443]:
```
λjl(BigFloat(5000), BigFloat(300))
```

[443]:
```
0.032769431881436593056666137886092306521561595675800102769380288936416809120920
59
```

[444]:
```
N(λ(10000, 200))
```

[444]:
```
0.036009906646684426440780547760034865817597274455767271744598938205464636453408
96
```

[445]:
```
λjl(BigFloat(10000), BigFloat(200))
```

[445]:
```
0.036009906646684426086009608220607938082101697109938732338886829788792716563352
82
```

[446]:
```
ρatm = ρn2(1.01325e5, 293.15)
```

[446]:
```
41.58105951222148
```

[447]:
```
N(λ(ρatm, 293.15))
```

[447]:
```
0.02547268399436569854852004147471750939605031705263036031149280542692716386774205
```

[448]:
```
N(λ(0, 300))
```

[448]:
```
0.02593608667121784255154131527931323480079714976118126498256590140203127919881928
```

[449]:
```
λjl(BigFloat(0), BigFloat(300))
```

[449]:
```
0.02593608667121784013963971373148787296652647801054477325138569618925646741186437
```

[450]:
```
N(λ(0, 100))
```

[450]:
```
0.00927749392871776713910514104183435305005724945855386717792512728375971553062074
```

[451]:
```
λjl(BigFloat(0), BigFloat(100))
```

[451]:
```
0.009277493928717769570614236010251825856602571761573130419573458478105581491863788
```

[452]:
```
N(λ(25000, 100))
```

[452]:
```
0.1038342450302420641403411394640530736047340847244110758645760258348835338671782
```

[453]:
```
λjl(BigFloat(25000), BigFloat(100))
```

[453]:
```
0.1038342450302421167092440470479961347902925704881382204594676233341878766113536
```

[454]:
```
N(λ(10000, 200))
```

[454]:
```
0.03600990664668442644078054776003486581759727445576727174459893820546463645340896
```

[455]:
```
λjl(BigFloat(10000), BigFloat(200))
```

[455]:
```
0.03600990664668442608600960822060793808210169710993873233888682978879271656335282
```

[456]:
```
N(λ(11.18*1000, 126.195))
```

[456]:
```
0.6758005439333020275702825386057180036631593812691489950818328132721624041932334
```

```
[457]: λjl(BigFloat(11.18*1000), BigFloat(126.195))
```

```
[457]: 0.675800543906010368981785532559248206105755976052774039976447777713859571563029
6
```

These values are consistent with Lemmon and Jacobsen [58].

Define a Julia function of pressure and temperature.

```
[458]: λn2pT(p1, T1) = λjl(ρn2(p1, T1), T1)
```

```
[458]: λn2pT (generic function with 1 method)
```

Warm surface value

```
[459]: λn2pT(BigFloat(ptab[1]), BigFloat(Ts))
```

```
[459]: 0.025472683994365702814421504599293940260682950639470834112253777475811761784260
63
```

Cold surface value

```
[460]: λn2pT(BigFloat(ptab[1]), BigFloat(Tcold))
```

```
[460]: 0.024112663646929646481173735052034339601905749433790170082932129165287497724007
13
```

Value at depth 1000 m (`dvals` index 1001)

```
[461]: λn2pT(BigFloat(ptab[1001]), BigFloat(Tcold))
```

```
[461]: 0.030115175702480974874904726458872704060556230069180431206078376955022603534857
43
```

Value at depth 2000 m (`dvals` index 2001)

```
[462]: λn2pT(BigFloat(ptab[2001]), BigFloat(Tcold))
```

```
[462]: 0.037676046997134363674845678979295960411033635881113047819727368138175814700286
05
```

Deep water value

```
[463]: λn2pT(BigFloat(ptab[end]), BigFloat(Tcold))
```

```
[463]: 0.049616519403474198830039019811598479498649519995845358011554803277948977062390
42
```

Calculate values for the depth profile.

```
[464]: λn2tab = map(p -> convert(Float64, λn2pT(BigFloat(p), BigFloat(Tcold))), ptab);
```

[465]:
```
λn2tab[1], λn2tab[1001], λn2tab[2001], λn2tab[end]
```

[465]:
```
(0.024112663646929648, 0.030115175702480974, 0.037676046997134366,
0.049616519403474196)
```

5.2.8. Thermal Diffusivity

Use the thermal diffusivity definition in Ainslie and Leighton [1].

[466]:
```
α(ρ1, T1) = ((λ(ρ1, T1) / (ρ1 * cp))(δ => ρ/ρc, τ => Tc/T))(ρ => ρ1,
    T => T1, M => MO2)
```

[466]:
```
α (generic function with 1 method)
```

Calculate a value. Recall that the `ρn2tab` array is a mass density. We must divide it by molar mass `MN2` to get molar density.

[467]:
```
N(α(ρn2tab[end]/MN2, Tcold))
```

[467]:
```
9.53771380581238216611683959570908533506480226109361649956403874857159298485278e
-08
```

Define a Julia function.

[468]:
```
αjl(ρ1, T1) = λjl(ρ1, T1) / (ρ1 * cpjl(ρ1, T1))
```

[468]:
```
αjl (generic function with 1 method)
```

[469]:
```
αjl(BigFloat(ρn2tab[end]/MN2), BigFloat(Tcold))
```

[469]:
```
9.537713805812383382597846633568194286885392932676980953772982349040826645988277
e-08
```

[470]:
```
αn2pT(p1, T1) = αjl(ρn2(p1, T1), T1)
```

[470]:
```
αn2pT (generic function with 1 method)
```

[471]:
```
αn2pT(BigFloat(ptab[end]), BigFloat(Tcold))
```

[471]:
```
9.53771380581238389620628978927454593976835927404818426253464594573344068200228e
-08
```

Warm surface value

[472]:
```
αn2pT(BigFloat(ptab[1]), BigFloat(Ts))
```

[472]:
```
2.100004083247022093503946771936644530470311760559136620822034024827056013541225
e-05
```

Cold surface value

[473]:
```
αn2pT(BigFloat(ptab[1]), BigFloat(Tcold))
```

[473]:
```
1.861946262897033998571748386675681825481124421867655633040351932072680469144438
e-05
```

Value at depth 1000 m (`dvals` index 1001)

[474]:
```
αn2pT(BigFloat(ptab[1001]), BigFloat(Tcold))
```

[474]:
```
1.913800129237647329608957909779612257816209851330371210293404763124807863569153
e-07
```

Value at depth 2000 m (`dvals` index 2001)

[475]:
```
αn2pT(BigFloat(ptab[2001]), BigFloat(Tcold))
```

[475]:
```
1.14299948890961679506500684513208826851235137428382537456727572856169743355277e
-07
```

Deep water value

[476]:
```
αn2pT(BigFloat(ptab[end]), BigFloat(Tcold))
```

[476]:
```
9.53771380581238389620628978927454593976835927404818426253464594573344068200228e
-08
```

Calculate values for the depth profile.

[477]:
```
αn2tab = map(p -> convert(Float64, αn2pT(BigFloat(p), BigFloat(Tcold))),
    ptab);
```

[478]:
```
αn2tab[1], αn2tab[1001], αn2tab[2001], αn2tab[end]
```

[478]:
```
(1.861946262897034e-5, 1.9138001292376474e-7, 1.1429994889096169e-7,
 9.537713805812384e-8)
```

Add these values to the dataframe.

[479]:
```
valsn2.thermal_diffusivity = αn2tab;
```

5.2.9. Export Nitrogen Parameters

We have all the needed nitrogen parameters in `valsn2`. Export them to a CSV file.

Here are the column names.

[480]:
```
names(valsn2)
```

[480]:
```
8-element Vector{String}:
 "depth"
 "pressure"
```

```
"water_density"
"water_dyn_viscosity"
"water_surface_tension"
"water_sound_speed"
"gamma"
"thermal_diffusivity"
```

Reorder the dataframe columns to match the order that the acoustic calculations require.

[481]:
```
select!(valsn2, ["depth", "water_density", "pressure", "water_dyn_viscosity",
        "water_surface_tension", "water_sound_speed",
        "thermal_diffusivity", "gamma"]);
```

[482]:
```
CSV.write("DeepWaterPropertiesN2.csv", valsn2)
```

[482]:
```
"DeepWaterPropertiesN2.csv"
```
