# Supplementary material for: An investigation of bubble resonance and its implications for sound production by deep-water fishes
Source: PLoS One. 2022 Jul 12;17(7):e0267338. doi: 10.1371/journal.pone.0267338 (PMC9275728; doi:10.1371/journal.pone.0267338)
Supplement: S4 File — PDF file created from S3 File. This file does not require Jupyter or Julia for viewing. (PDF) [file pone.0267338.s004.pdf]

Deep Water Bubble Calculations

1. Introduction

In this Jupyter notebook we perform the calculations for the paper Mark W. Sprague, Michael L. Fine, and Timothy M. Cameron (2022), "An investigation of bubble resonance and its implications for sound production by deep-water fishes," with documentation/explanation of each step. Refer to the paper for the references cited in this notebook. This notebook runs in the Julia programming language. Julia version 1.7.2 was used for all calculations in the paper.

We use following Julia packages for the calculations in this notebook.

- CSV - read parameter values stored in CSV files.
- DataFrames - store parameter values in dataframes.
- Interpolations - interpolate between parameter values calculated at specific depth. Gas and water parameters were calculated for a range of depths in a different notebook. We import the calculated parameter values and create interpolating functions to provide parameters at any depth in the range. All interpolations use a cubic spline method.
- SymPy (which calls the SymPy Python package) - symbolic expressions and symbolic calculations. Once each expression is in a form for numerical calculations, a Julia numerical function is generated with the SymPy `lambdify` function.
- Roots - numerically solving equations.
- Optim - find the maximum values of parameters. We used the `optimize` function to find a constrained minimum of the negative of the parameter using Brent's method.
- PyPlot - generate figures using the Python Matpoltlib package.
- PyCall - generate a Python function needed to customize the PyPlot figure format.
- Statistics - use the mean and max and std functions.

Some of the symbolic calculations in this notebook produce long symbolic expressions as output. These expressions are many lines long and often do not fit within the page margins or screen size. Output from these expressions has been suppressed using a trailing semicolon (`;`). To view the output, delete the trailing semicolon before entering the input cell.

2. Initializations

Load the packages used in the notebook.

[1]:
```
using CSV, DataFrames, Interpolations
using SymPy, Roots, Optim, PyPlot, PyCall
import Statistics: mean, max, std
```

Import SymPy symbols into the document.

[2]:
```
import_from(sympy)
```

This function that executes Python code to interface with PyPlot (which also runs in Python) will make it easier to generate subplots with the PyPlot `gridspec` method.

[3]:
```
slice(i,j) = pycall(pybuiltin("slice"), PyObject, i,j)
```

[3]:
```
slice (generic function with 1 method)
```

3. Parameters

Define the parameters and value substitutions.

First define the imaginary number i.

[4]:
```
i = IM
```

[4]:
i

Define the symbols for use in mathematical expressions.

[5]:
```
ρ, pw, μ, σ, c, dd, γ, a, ω = symbols("ρ, pw, μ, σ, c, dd, γ, a, ω", real=true)
```

[5]:
```
(ρ, pw, μ, σ, c, dd, γ, a, ω)
```

Define f as a variable.

[6]:
```
@vars f
```

[6]:
```
(f,)
```

[7]:
```
"""
    vals(gas, depth, temp="w")

Function to produce a Dictionary with numerical values for each of the
water and gas parameters in a specified environment. The resulting
dictionary can be used to substitute these values for the symbols in a
symbolic expression.

# Arguments
`gas` - type of gas in the bubble. Use `"N"`, `"N2"`, or `"n2"`
            for nitrogen. Use `"O"`, `"O2"`, or `"o2"` for oxygen.\n
`depth` - depth of the environment. Use `0`, `"S"`, `"s"`, or
            `"surface"` for 0 m. Use `7.2`, `"Sh"`, `"sh"`, or
            `"shallow"` for 7.2 m. Use `1000` for 1000 m. Use `2000`
            for 2000 m. Use `3500`, `"D"`, `"d"`, or
            `"deep"` for 3500 m (deep water). Use `7200`, `"dd"`,
            `"DD"`, or `"very deep"` for 7200 m (very deep)\n
`temp` - optional parameter for the water temperature (only for
            the sueface and shallow water) environments. Use `"w"` or
            `"warm"` for the warm water (20 °C) environment. Use `"c"`
```

```
            or `"cold"` for the cold water environment (1.50 °C). The
            default value is `"warm"`.\n
# Output Dictionary Keys
`"ρ"` - water density (kg/m^3)\n
`"pw"` - ambient pressure (Pa)\n
`"μ"` - water dynamic viscosity (Pa s)\n
`"σ"` - water surface tension (N/m)\n
`"c"` - water sound speed (m/s)\n
`"dd"` - gas thermal diffusivity (m^2/s)\n
`"γ"` - gas ratio of specific heats

"""
function vals(gas, depth; temp="w")
    if (gas == "N") | (gas == 1) | (gas == "N2") | (gas == "n2")
        if (depth == 0) | (depth == "s") | (depth == "S") | (depth == "surface")
            if (temp == "w") | (temp == "warm")
                return (
                    [ρ, pw, μ, σ, c, dd, γ] .=>
                    (1028, 1.01325e5, 1.077e-3, 7.352e-2, 1522, 2.100e-5, 1.401)
                )
            elseif (temp == "c") | (temp == "cold")
                return (
                    [ρ, pw, μ, σ, c, dd, γ] .=>
                    (1027, 1.01325e5, 1.812e-3, 7.601e-2, 1456, 1.862e-5, 1.402)
                )
            end
        elseif depth == 1000
            return (
                [ρ, pw, μ, σ, c, dd, γ] .=>
                (1032, 1.019e7, 1.812e-3, 7.601e-2, 1473, 1.914e-7, 1.612)
            )
        elseif depth == 2000
            return (
                [ρ, pw, μ, σ, c, dd, γ] .=>
                (1036, 2.033e7, 1.812e-3, 7.601e-2, 1490, 1.143e-7, 1.738)
            )
        elseif (depth == 3500) | (depth == "d") | (depth == "D") | (depth == "deep")
            return (
                [ρ, pw, μ, σ, c, dd, γ] .=>
                (1043, 3.562e7, 1.812e-3, 7.601e-2, 1516, 9.538e-8, 1.760)
            )
        end
    elseif (gas == "O") | (gas == 2) | (gas == "O2") | (gas == "o2")
        if (depth == 0) | (depth == "s") | (depth == "S") | (depth == "surface")
            if (temp == "w") | (temp == "warm")
                return (
                    [ρ, pw, μ, σ, c, dd, γ] .=>
```

```
                    (1028, 1.01325e5, 1.077e-3, 7.352e-2, 1522, 2.1210e-5, 1.397)
                )
            elseif (temp == "c") | (temp == "cold")
                return (
                    [ρ, pw, μ, σ, c, dd, γ] .=>
                    (1027, 1.01325e5, 1.812e-3, 7.601e-2, 1456, 1.878e-5, 1.400)
                )
            end
        elseif depth == 1000
            return (
                [ρ, pw, μ, σ, c, dd, γ] .=>
                (1032, 1.019e7, 1.812e-3, 7.601e-2, 1473, 1.720e-7, 1.668)
            )
        elseif depth == 2000
            return (
                [ρ, pw, μ, σ, c, dd, γ] .=>
                (1036, 2.033e7, 1.812e-3, 7.601e-2, 1490, 9.264e-8, 1.897)
            )
        elseif (depth == 3500) | (depth == "d") | (depth == "D") | (depth == "deep")
            return (
                [ρ, pw, μ, σ, c, dd, γ] .=>
                (1043, 3.562e7, 1.812e-3, 7.601e-2, 1516, 7.487e-8, 1.950)
            )
        end
    end
    error("Values not found.")
end

"""
    ρ, pw, μ, σ, c, dd, γ = valst(gas, depth[, temp])

Function to call `vals` and return the parameters values in an Array. This function is␣
  ↪useful for supplying the parameters to a
Julia function.

# Arguments
`gas` - type of gas in the bubble. Use `"N"`, `"N2"`, or `"n2"`
            for nitrogen. Use `"O"`, `"O2"`, or `"o2"` for oxygen.\n
`depth` - depth of the environment. Use `0`, `"S"`, `"s"`, or
            `"surface"` for 0 m. Use `7.2`, `"Sh"`, `"sh"`, or
            `"shallow"` for 7.2 m. Use `1000` for 1000 m. Use `2000`
            for 2000 m. Use `3500`, `"D"`, `"d"`, or
            `"deep"` for 3500 m (deep water). Use `7200`, `"dd"`,
            `"DD"`, or `"very deep"` for 7200 m (very deep)\n
`temp` - optional parameter for the water temperature (only for
            the sueface and shallow water) environments. Use `"w"` or
            `"warm"` for the warm water (20 °C) environment. Use `"c"`
```

```
            or `"cold"` for the cold water environment (1.50 °C). The
            default value is `"warm"`.\n
# Output
`ρ` - water density (kg/m^3)\n
`pw` - ambient pressure (Pa)\n
`μ` - water dynamic viscosity (Pa s)\n
`σ` - water surface tension (N/m)\n
`c` - water sound speed (m/s)\n
`dd` - gas thermal diffusivity (m^2/s)\n
`γ` - gas ratio of specific heats

"""
function valst(gas, depth; temp="w")
    v1 = Dict(vals(gas, depth, temp=temp))
    return v1[ρ], v1[pw], v1[μ], v1[σ], v1[c], v1[dd], v1[γ]
end
```

[7]: valst

Define a list of the symbols in the same order as the output of values in the `valst` function.

[8]:
```
vlist = (ρ, pw, μ, σ, c, dd, γ)
```

[8]: (ρ, pw, μ, σ, c, dd, γ)

4. Property interpolation with depth

4.1. Oxygen

Read calculated parameter values for oxygen bubbles into a dataframe. This data file was produced by the S1 Notebook in Section 5.1.9.

[9]:
```
valso2 = CSV.read("DeepWaterPropertiesO2.csv", DataFrame);
```

[10]:
```
names(valso2)
```

[10]:
```
8-element Vector{String}:
 "depth"
 "water_density"
 "pressure"
 "water_dyn_viscosity"
 "water_surface_tension"
 "water_sound_speed"
 "thermal_diffusivity"
 "gamma"
```

Define an array containing interpolation functions for each column of the dataframe.

[11]:
```
ivalo2 = Array{ScaledInterpolation}(undef,7)
for n in 1:7
    itp = Interpolations.interpolate(valso2[:,n+1], BSpline(Cubic(Line(OnGrid()))))
    ivalo2[n] = scale(itp, (valso2[1,1]:valso2[end,1]))
end
```

Define a function that returns a Dictionary of parameter values for a given depth. This dictionary is useful for substituting into symbolic expressions.

[12]:
```
"""
    valsfo2(d)
Return a Dictionary of interpolated parameter values for oxygen bubbles at depth `d`.
"""
function valsfo2(d)
    flist = (ivalo2[n](d) for n in 1:7)
    vlist .=> flist
end
```

[12]: valsfo2

Define a function that returns an array of parameter values for a given depth. This array is useful for providing values to functions.

[13]:
```
"""
    valsfo2list(d)
Return an Array of interpolated parameter values for oxygen bubbles at depth `d`.
"""
function valso2list(d)
    collect(ivalo2[n](d) for n in 1:7)
end
```

[13]: valso2list

4.2. Nitrogen

Read calculated parameter values for nitrogen bubbles into a dataframe. This data file was produced by the S1 Notebook in Section 5.2.9.

[14]:
```
valsn2 = CSV.read("../GasParameters/ValsN2.csv", DataFrame, header=false,␣
  ↪transpose=true);
```

Define an array containing interpolation functions for each column of the dataframe.

[15]:
```
ivaln2 = Array{ScaledInterpolation}(undef,7)
for n in 1:7
    itp = Interpolations.interpolate(valsn2[:,n+1], BSpline(Cubic(Line(OnGrid()))))
    ivaln2[n] = scale(itp, (valsn2[1,1]:valsn2[end,1]))
end
```

Define a function that returns a Dictionary of parameter values for a given depth. This dictionary is useful for substituting into symbolic expressions.

[16]:
```
"""
    valsfn2(d)
Return a Dictionary of interpolated parameter values for nitrogen bubbles at depth `d`.
"""
function valsfn2(d)
    flist = (ivaln2[n](d) for n in 1:7)
    vlist .=> flist
end
```

[16]: valsfn2

Define a function that returns an array of parameter values for a given depth. This array is useful for providing values to functions.

[17]:
```
"""
    valsfn2list(d)
Return an Array of interpolated parameter values for nitrogen bubbles at depth `d`.
"""
function valsn2list(d)
    collect(ivaln2[n](d) for n in 1:7)
end
```

[17]: valsn2list

5. Bubble Size and Resonance

5.1. Definitions

The equation numbers given here are those in Ainslie and Leighton [1]. Note: We use `dd` to represent the thermal diffusivity, D_p in Ainslie and Leighton [1].

The following expression is Eq. (8).

[18]:
```
lth = sqrt(dd / (2ω))
```

[18]:
$$\frac{\sqrt{2}\sqrt{\frac{dd}{\omega}}}{2}$$

The following expression for the bubble Laplace radius is Eq. (11). The parameter `σ` is τ in Ainslie and Leighton [1] is , and `pw` is P_{liq}.

[19]:
```
RLap = 2σ / pw
```

[19]:
$$\frac{2\sigma}{pw}$$

As a check, use the dictionary generated by the `vals` function to generate a numerical value.

[20]:
```
N(RLap(vals("n2", 3500)...))
```

[20]: 4.267827063447501e-9

The following equation is Eq. (12) for the natural angular frequency. This is the Minnaert angular frequency.

[21]:
```
ωM = expand_power_base(1/a * sqrt(3γ * pw / ρ), force=true)
```

[21]: $\frac{\sqrt{3}\sqrt{pw}\sqrt{\gamma}\sqrt{\frac{1}{\rho}}}{a}$

As a check, generate a numerical value for `ωM` using `vals`.

[22]:
```
N(ωM(vals("n2", 3500)..., a=>0.01))
```

[22]: 42464.08442394073539373615396379947556004437812655596490962434667038011121324344

Define a Julia function for the Minneart frequency.

[23]:
```
fMjl = lambdify(ωM/(2*π), (a, vlist...))
```

[23]: #118 (generic function with 1 method)

[24]:
```
2*π * fMjl(0.01, valst("n2", 3500)...)
```

[24]: 42464.08442394074

The following is Eq. (14) for the thermal diffusion ratio. The parameter `a` is R_0, the bubble radius, in Ainslie and Leighton [1].

[25]:
```
X = expand_power_base(a / lth, force=true)
```

[25]: $\frac{\sqrt{2}a}{\sqrt{dd}\sqrt{\frac{1}{\omega}}}$

As a check, generate a numerical value for `X` using `vals`.

[26]:
```
N(X(vals("n2", 3500)..., a=>0.01, ω=>2*π*500))
```

[26]: 2566.618233025503586652631289132593889747080961471964214565849780904802650517199

The following is Eq. (16) for the complex polytropic index.

Symbolic output from the expression below has been suppressed. To show the output, delete the trailing semicolon before entering the entering the expression.

[27]:
```
Γ = simplify(γ /
    (1 -
        (((1 + i) * X/2) / (tanh((1 + i) * X/2)) - 1) *
        (6 * i * (γ - 1)) / (X^2)
    )
);
```

As a check, generate a numerical value for `Γ` using `vals`.

[28]:
```
N(Γ(vals("n2", 3500)..., a=>0.01, ω=>2*π*500))
```

[28]: 1.7584365465467842 + 0.0015594666757119 14im

We solve Eq. (15) for ω to get the resonant frequency ω_{res}.

First enter an expression for Eq. (15). Each defined parameter is expanded in the resulting expression.

Symbolic output from the expression below has been suppressed. To show the output, delete the trailing semicolon before entering the entering the expression.

[29]:
```
eq15 = Eq(
    γ * ω^2 / ωM^2,
    (1 + RLap/a) * re(Γ) - RLap / (3a)
);
```

Now we can use `vals` and enter numerical values for `a` and `ω` to evaluate the left and right sides of the equation. We will use this below to find numerical solutions.

[30]:
```
(N(lhs(eq15)(vals("n2", 3500)..., a=>0.01, ω=>2*π*500)), N(rhs(eq15)(vals("n2", 3500)...
↪, a=>0.01, ω=>2*π*500)))
```

[30]: (0.009633162446505897, 1.758437154756190352918898161325051116620666094925326833635863147467430535808923)

Note that if we enter values for `a` and the parameters supplied by the `vals` function, both sides of Eq. (15) become functions of `ω` only. We will use this to solve numerically for frequency values.

Symbolic output from the expression below has been suppressed. To show the output, delete the trailing semicolon before entering the entering the expression.

[31]:
```
eq15(a=>0.1, vals("n2", 0)...);
```

The following is Eq. (34) for the dimensionless frequency.

[32]:
```
ϵ = ω * a / c
```

[32]: $\frac{a\omega}{c}$

As a check, generate a numerical value for `ϵ` using `vals`.

[33]:
```
N(ϵ(vals("n2", 3500)..., a=>0.01, ω=>2*π*500))
```

[33]: 0.020722906685948502

This is Eq. (46) for the equilibrium pressure inside the bubble.

[34]:
```
pgas = pw + 2σ/a
```

[34]: $pw + \frac{2\sigma}{a}$

As a check, generate a numerical value for `pgas` using `vals`.

[35]:
```
N(pgas(vals("n2", 3500)..., a=>0.01, ω=>2*π*500))
```

[35]:
```
3.5620015202e7
```

This is Eq. (93) for a frequency-dependent parameter related to the resonant frequency.

Symbolic output from the expression below has been suppressed. To show the output, delete the trailing semicolon before entering the entering the expression.

[36]:
```
ω0 = simplify(sqrt(3 * re(Γ) * pgas / (ρ * a^2) - 2 * σ / (ρ * a^3)));
```

As a check, generate a numerical value for `ω0` using `vals`.

[37]:
```
N(ω0(vals("n2", 3500)..., a=>0.01, ω=>2*π*500))
```

[37]:
```
42445.22660247035756165512249712223906496083819417985503674056170985506937390732
```

This is Eq. (87) for the stiffness parameter `K`.

Symbolic output from the expression below has been suppressed. To show the output, delete the trailing semicolon before entering the entering the expression.

[38]:
```
K = simplify(ω0^2 + ϵ^2 / (1 + ϵ^2) * ω^2);
```

As a check, generate a numerical value for `K` using `vals`.

[39]:
```
N(K(vals("n2", 3500)..., a=>0.01, ω=>2*π*500))
```

[39]:
```
1.801601497907386526890443524370992964559564711017030564728345747037665056562364
e+09
```

This is Eq. (91) for the viscous damping factor. The parameter `μ` is η_s, the shear viscosity coefficient of the liquid, in Ainslie and Leighton [1].

[40]:
```
βvis = 2μ / (ρ * a^2)
```

[40]: $\frac{2\mu}{a^2\rho}$

As a check, generate a numerical value for `βvis` using `vals`.

[41]:
```
N(βvis(vals("n2", 3500)..., a=>0.01, ω=>2*π*500))
```

[41]:
```
0.03474592521572387
```

This is Eq. (92) for the thermal damping factor.

Symbolic output from the expression below has been suppressed. To show the output, delete the trailing semicolon before entering the entering the expression.

[42]:
```
βth = simplify((3 * pgas) / (2ρ * a^2 * ω) * imag(Γ));
```

As a check, generate a numerical value for `βvth` using `vals`.

[43]:
```
N(βth(vals("n2", 3500)..., a=>0.01, ω=>2*π*500))
```

[43]:
```
254.2888262311701877290558552074788321531971508530733444014724842977070258107796
```

This is Eq. (90) for the non-acoustic damping factor.

Symbolic output from the expression below has been suppressed. To show the output, delete the trailing semicolon before entering the entering the expression.

[44]:
```
β0 = βvis + βth;
```

As a check, generate a numerical value for `β0` using `vals`.

[45]:
```
N(β0(vals("n2", 3500)..., a=>0.01, ω=>2*π*500))
```

[45]:
```
254.3235721563859116022697906983456204669212514428255416670974842977070258107796
```

This is Eq. (88) for the (total) damping factor.

Symbolic output from the expression below has been suppressed. To show the output, delete the trailing semicolon before entering the entering the expression.

[46]:
```
β = β0 + ϵ / (1 + ϵ^2) * ω/2;
```

As a check, generate a numerical value for `β` using `vals`.

[47]:
```
N(β(vals("n2", 3500)..., a=>0.01, ω=>2*π*500))
```

[47]:
```
286.8610649953059419810940236955492009199818188218233444014724842977070258107819
```

5.2. Resonant Frequency

Convert the symbolic expression `eq15` to a Julia function so we can find a numerical solution (right side - left side = 0).

[48]:
```
eq15jl = lambdify((lhs(eq15) - rhs(eq15))(ω=>2*pi*f),(a, vlist..., f))
```

[48]:
```
#118 (generic function with 1 method)
```

Now define a function to find frequencies for the zeros of `eq15jl` with supplied parameters.

[49]:
```
"""
    fres(a1, v1[, f0])
Find the resonance frequency that is a numerical solution `eq15jl`.
The argment `f0` is the optional starting value for the numerical
solution with default value 200 Hz.
"""
fres(a1, v1, f0=200) = find_zero(eq15jl(a1, v1..., f), f0)
fres(a1, v1; f0=200) = find_zero(eq15jl(a1, v1..., f), f0)
```

[49]:
```
fres (generic function with 2 methods)
```

5.3. Natural Frequency

To solve Eq. (124) for the natural oscillation frequency we subtract the right side from the left side so we can find the root numerically.

Symbolic output from the expression below has been suppressed. To show the output, delete the trailing semicolon before entering the entering the expression.

[50]:
```
eq124z = ω^2 - (K - β^2);
```

Convert the symbolic expression `eq124z` to a Julia function so we can find a numerical solution (right side - left side = 0).

[51]:
```
eq124zjl = lambdify(eq124z(ω=>2*pi*f),(a,vlist...,f))
```

[51]:
```
#118 (generic function with 1 method)
```

Now define a function to find frequencies for the zeros of `eq124zjl` with supplied parameters.

[52]:
```
"""
    fnat(a1, v1[, f0])
Find the natural frequency of oscillation that is a numerical solution
`eq124zjl`. The argment `f0` is the optional starting value for the
numerical solution with default value 200 Hz.
"""
fnat(a1, v1, f0) = find_zero(eq124zjl(a1, v1..., f), f0)
fnat(a1, v1; f0=200) = find_zero(eq124zjl(a1, v1..., f), f0)
```

[52]:
```
fnat (generic function with 2 methods)
```

As a check, evaluate the function for some parameters.

[53]:
```
fnat(0.1, valst("n2", 0))
```

[53]:
```
32.35148197122246
```

5.4. Far-Field Resonance

This is Eq. (85) for the scattering cross section. This relationship is from Eq. (43) in a previous study by Ainslie and Leighton [42]. Equation (149) in Ainslie and Leighton [1] is based on this with ω_0 and β_0 held constant.

Symbolic output from the expression below has been suppressed. To show the output, delete the trailing semicolon before entering the entering the expression.

[54]:
```
σs = 4 * pi * a^2 / ((ω0^2/ω^2 - 1 - 2 * β0 * ϵ / ω)^2 + (2 * β0/ω + ω0^2 * ϵ/ω^2)^2);
```

Convert the symbolic expression `σs` to a Julia function so we can find a numerical solution for the maximum value.

[55]:
```
σsjl = lambdify(σs(ω=>2*pi*f),(a, vlist..., f))
```

[55]: #118 (generic function with 1 method)

Now define a set of functions that maximizes `σsjl` returning the frequency of the maximum and the maximum value.

[56]:
```
"""
   fff(a1, v1[, f1, f2])
Numerically determine the resonance frequency that maximizes the value
of `σsjl` between frequencies `f1` and `f2`. `f1` and `f2` are optional
arguments with default values 0 Hz and 10000 Hz respectively.
"""
fff(a1, v1, f1, f2) = optimize(fin -> -σsjl(a1, v1..., fin), f1, f2).minimizer
fff(a1, v1; f1=0.0, f2=10000.0) = optimize(fin -> -σsjl(a1, v1..., fin), f1, f2).minimizer

"""
   σres(a1, v1[, f1, f2])
Numerically determine the maximum value of `σsjl` between frequencies
`f1` and `f2`. `f1` and `f2` are optional arguments with default
values 0 Hz and 10000 Hz respectively.
"""
σres(a1, v1, f1, f2) = -optimize(fin -> -σsjl(a1, v1..., fin), f1, f2).minimum
σres(a1, v1; f1=0.0, f2=10000.0) = -optimize(fin -> -σsjl(a1, v1..., fin), f1, f2).minimum
```

[56]: σres (generic function with 2 methods)

5.5. Far-field pressure resonance plot

Define a range of bubble radii for evaluation of radius dependencies and for plotting.

[57]:
```
avals = 0.01:0.005:0.2
```

[57]: 0.01:0.005:0.2

5.5.1. Calculation of far-field resonance frequencies

Arrays of `fff` values are calculated below for the bubble radii in `avals`.

[58]:
```
fffns = map(a1 -> fff(a1, valst("n2", 0)), avals);
```

[59]:
```
fffncs = map(a1 -> fff(a1, valst("n2", 0, temp="c")), avals);
```

[60]:
```
fffn1k = map(a1 -> fff(a1, valst("n2", 1000)), avals);
```

[61]:
```
fffn2k = map(a1 -> fff(a1, valst("n2", 2000)), avals);
```

[62]:
```
fffnd = map(a1 -> fff(a1, valst("n2", 3500)), avals);
```

[63]:
```
fffos = map(a1 -> fff(a1, valst("o2", 0)), avals);
```

[64]:
```
fffocs = map(a1 -> fff(a1, valst("o2", 0, temp="c")), avals);
```

[65]:
```
fffo1k = map(a1 -> fff(a1, valst("o2", 1000)), avals);
```

[66]:
```
fffo2k = map(a1 -> fff(a1, valst("o2", 2000)), avals);
```

[67]:
```
fffod = map(a1 -> fff(a1, valst("o2", 3500)), avals);
```

5.5.2. Plotting far-field resonance frequencies

Plot the far-field resonance frequencies vs. bubble radius. This is Fig 1B.

[68]:
```
plot(avals, fffns, linestyle="-", color=get_cmap("plasma")(0), label=L"N$_2$ Warm␣
  ↪Surface")
plot(avals, fffos, linestyle="--", color=get_cmap("plasma")(0.1), label=L"O$_2$ Warm␣
  ↪Surface")
plot(avals, fffncs, linestyle="-", color=get_cmap("plasma")(0.2), label=L"N$_2$ Cold␣
  ↪Surface")
plot(avals, fffocs, linestyle="--", color=get_cmap("plasma")(0.3), label=L"O$_2$ Cold␣
  ↪Surface")
plot(avals, fffn1k, linestyle="-", color=get_cmap("plasma")(0.4), label=L"N$_2$ 1000 m␣
  ↪Deep")
plot(avals, fffo1k, linestyle="--", color=get_cmap("plasma")(0.5), label=L"O$_2$ 1000 m␣
  ↪Deep")
plot(avals, fffn2k, linestyle="-", color=get_cmap("plasma")(0.6), label=L"N$_2$ 2000 m␣
  ↪Deep")
plot(avals, fffo2k, linestyle="--", color=get_cmap("plasma")(0.7), label=L"O$_2$ 2000 m␣
  ↪Deep")
plot(avals, fffnd, linestyle="-", color=get_cmap("plasma")(0.8), label=L"N$_2$ 3500 m␣
  ↪Deep")
plot(avals, fffod, linestyle="--", color=get_cmap("plasma")(0.9), label=L"O$_2$ 3500 m␣
  ↪Deep")
xlim(-0.002, 0.202)
ylim(-75, 7575)
xlabel("Bubble Radius (m)")
ylabel("Frequency (Hz)")
legend();
```

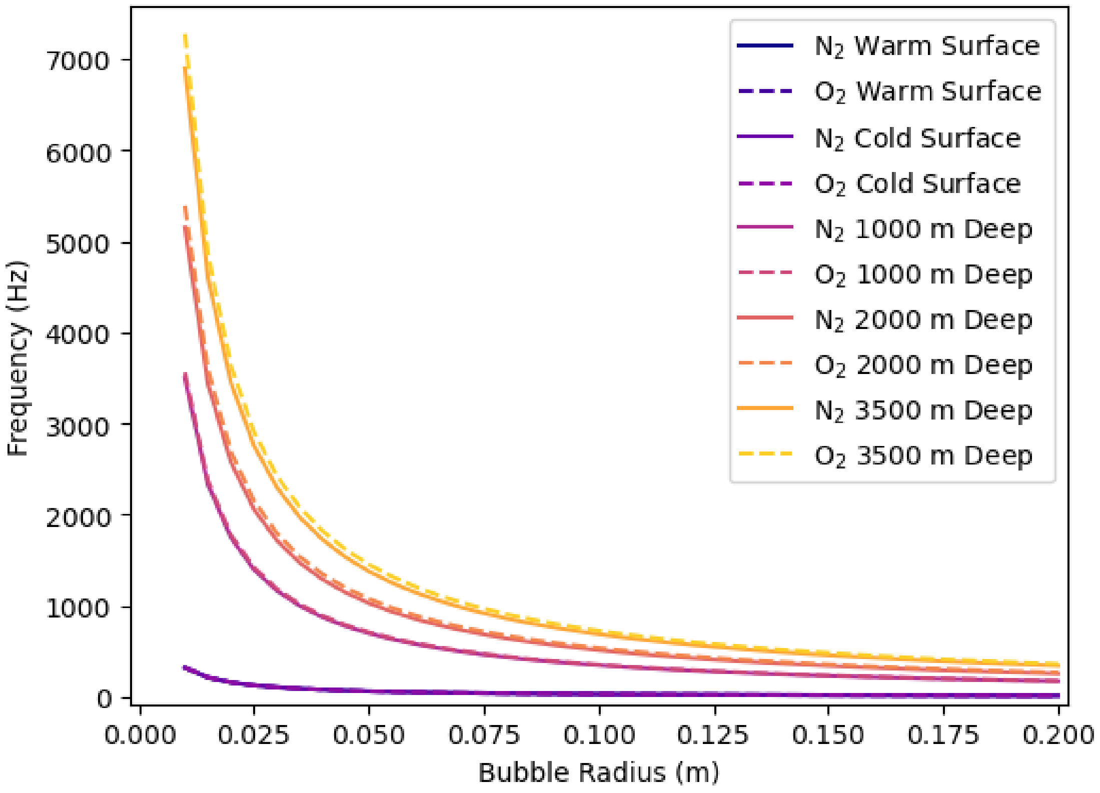

This is Fig. 1B in the paper. (The entire Fig. 1 is generated below.)

5.6. Undamped frequency

The frequency for undamped motion is also the frequency of maximum bubble wall velocity defined in Eq. (141). The expression below represents Eq. (141).

Symbolic output from the expression below has been suppressed. To show the output, delete the trailing semicolon before entering the entering the expression.

[69]:
```
funeq = sqrt(K) - ω;
```

Convert the symbolic expression `funeq` to a Julia function so we can find a numerical solution.

[70]:
```
funeqjl = lambdify(funeq(ω=>2*pi*f),(a, vlist..., f))
```

[70]: `#118 (generic function with 1 method)`

Now define a function to find the roots of `funeqjl`.

[71]:
```
"""
    fun(a1, v1[, f0])
Find the frequency that is the root of `funeqjl`. The optional argument
`f0` is the initial value for the `find_zero` function with default
```

```
value 20 Hz.
"""
fun(a1, v1, f0) = find_zero(f1 -> funeqjl(a1, v1..., f1), f0)
fun(a1, v1; f0=20.0) = find_zero(f1 -> funeqjl(a1, v1..., f1), f0)
fun(a1, ρ, pw, μ, σ, c, dd, γ, f0) = find_zero(f1 ->
    funeqjl(a1, ρ, pw, μ, σ, c, dd, γ, f1), f0)
fun(a1, ρ, pw, μ, σ, c, dd, γ; f0=20.0) = find_zero(f1 ->
    funeqjl(a1, ρ, pw, μ, σ, c, dd, γ, f1), f0)
```

[71]:
```
fun (generic function with 4 methods)
```

Arrays of `fun` values are calculated below for the bubble radii in `avals`.

[72]:
```
funns = map(a->fun(a, valst("n2", 0)), avals);
```

[73]:
```
funos = map(a->fun(a, valst("o2", 0)), avals);
```

[74]:
```
funncs = map(a->fun(a, valst("n2", 0, temp="c")), avals);
```

[75]:
```
funocs = map(a->fun(a, valst("o2", 0, temp="c")), avals);
```

[76]:
```
funn1k = map(a->fun(a, valst("n2", 1000)), avals);
```

[77]:
```
funo1k = map(a->fun(a, valst("o2", 1000)), avals);
```

[78]:
```
funn2k = map(a->fun(a, valst("n2", 2000)), avals);
```

[79]:
```
funo2k = map(a->fun(a, valst("o2", 2000)), avals);
```

[80]:
```
funnd = map(a->fun(a, valst("n2", 3500)), avals);
```

[81]:
```
funod = map(a->fun(a, valst("o2", 3500)), avals);
```

Plot the undamped resonance frequencies vs. bubble radius. This is Fig 1A.

[82]:
```
plot(avals, funns, linestyle="-", color=get_cmap("plasma")(0), label=L"N$_2$ Warm␣
  ↪Surface")
plot(avals, funos, linestyle="--", color=get_cmap("plasma")(0.1), label=L"O$_2$ Warm␣
  ↪Surface")
plot(avals, funncs, linestyle="-", color=get_cmap("plasma")(0.2), label=L"N$_2$ Cold␣
  ↪Surface")
plot(avals, funocs, linestyle="--", color=get_cmap("plasma")(0.3), label=L"O$_2$ Cold␣
  ↪Surface")
plot(avals, funn1k, linestyle="-", color=get_cmap("plasma")(0.4), label=L"N$_2$ 1000 m␣
  ↪Deep")
plot(avals, funo1k, linestyle="--", color=get_cmap("plasma")(0.5), label=L"O$_2$ 1000 m␣
  ↪Deep")
```

```
plot(avals, funn2k, linestyle="-", color=get_cmap("plasma")(0.6), label=L"N$_2$ 2000 m␣
  ↪Deep")
plot(avals, funo2k, linestyle="--", color=get_cmap("plasma")(0.7), label=L"O$_2$ 2000 m␣
  ↪Deep")
plot(avals, funnd, linestyle="-", color=get_cmap("plasma")(0.8), label=L"N$_2$ 3500 m␣
  ↪Deep")
plot(avals, funod, linestyle="--", color=get_cmap("plasma")(0.9), label=L"O$_2$ 3500 m␣
  ↪Deep")
legend()
xlim(-0.002, 0.202)
ylim(-75, 7575)
xlabel("Bubble Radius (m)")
ylabel("Frequency (Hz)");
```

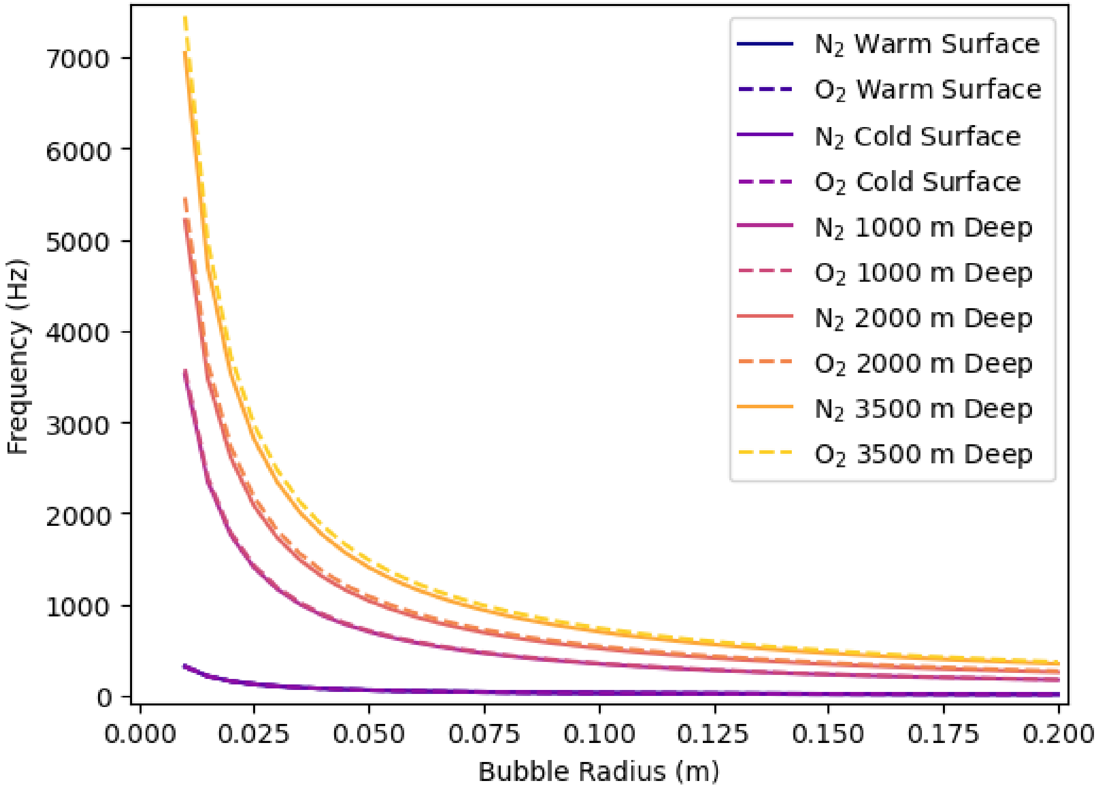

This is Fig. 1A in the paper. The entire Fig. 1 is plotted below.

5.7. Figure 1

Plot the undamped and far-field resonance frequencies vs. bubble radius. This is Fig. 1 in the paper.

[83]:

```
heights = [3, 1]
fig = figure(constrained_layout=true, dpi=300, figsize=(7.38, 3))
gs = fig.add_gridspec(2, 2, height_ratios=heights)
ax1 = fig.add_subplot(get(gs, (0, 0)))
ax1.plot(avals, funns, linestyle="-", color=get_cmap("plasma")(0), label=L"N$_2$ Warm␣
↪Surface")
ax1.plot(avals, funos, linestyle="--", color=get_cmap("plasma")(0.1), label=L"O$_2$ Warm␣
↪Surface")
ax1.plot(avals, funncs, linestyle="-", color=get_cmap("plasma")(0.2), label=L"N$_2$ Cold␣
↪Surface")
ax1.plot(avals, funocs, linestyle="--", color=get_cmap("plasma")(0.3), label=L"O$_2$␣
↪Cold Surface")
ax1.plot(avals, funn1k, linestyle="-", color=get_cmap("plasma")(0.4), label=L"N$_2$ 1000␣
↪m Deep")
ax1.plot(avals, funo1k, linestyle="--", color=get_cmap("plasma")(0.5), label=L"O$_2$␣
↪1000 m Deep")
ax1.plot(avals, funn2k, linestyle="-", color=get_cmap("plasma")(0.6), label=L"N$_2$ 2000␣
↪m Deep")
ax1.plot(avals, funo2k, linestyle="--", color=get_cmap("plasma")(0.7), label=L"O$_2$␣
↪2000 m Deep")
ax1.plot(avals, fundd, linestyle="-", color=get_cmap("plasma")(0.8), label=L"N$_2$ 3500␣
↪m Deep")
ax1.plot(avals, funod, linestyle="--", color=get_cmap("plasma")(0.9), label=L"O$_2$ 3500␣
↪m Deep")
#ax1.set_aspect(0.618 * 0.2 / 7500)
ax1.set_xlim(-0.002, 0.202)
ax1.set_ylim(-75, 7575)
ax1.set_xlabel("Bubble Radius (m)")
ax1.set_ylabel("Frequency (Hz)")

ax2 = fig.add_subplot(get(gs, (0, 1)))
ax2.plot(avals, fffns, linestyle="-", color=get_cmap("plasma")(0))
ax2.plot(avals, fffos, linestyle="--", color=get_cmap("plasma")(0.1))
ax2.plot(avals, fffncs, linestyle="-", color=get_cmap("plasma")(0.2))
ax2.plot(avals, fffocs, linestyle="--", color=get_cmap("plasma")(0.3))
ax2.plot(avals, fffn1k, linestyle="-", color=get_cmap("plasma")(0.4))
ax2.plot(avals, fffo1k, linestyle="--", color=get_cmap("plasma")(0.5))
ax2.plot(avals, fffn2k, linestyle="-", color=get_cmap("plasma")(0.6))
ax2.plot(avals, fffo2k, linestyle="--", color=get_cmap("plasma")(0.7))
ax2.plot(avals, fffnd, linestyle="-", color=get_cmap("plasma")(0.8))
ax2.plot(avals, fffod, linestyle="--", color=get_cmap("plasma")(0.9))
#ax2.set_aspect(0.618 * 0.2 / 7500)
ax2.set_xlim(-0.002, 0.202)
ax2.set_ylim(-75, 7575)
ax2.set_xlabel("Bubble Radius (m)")
ax2.set_ylabel("Frequency (Hz)")
```

```
fig.text(0.01, 0.955, "A")
fig.text(0.51, 0.955, "B")
fig.legend(ncol=5, loc="lower center");
```

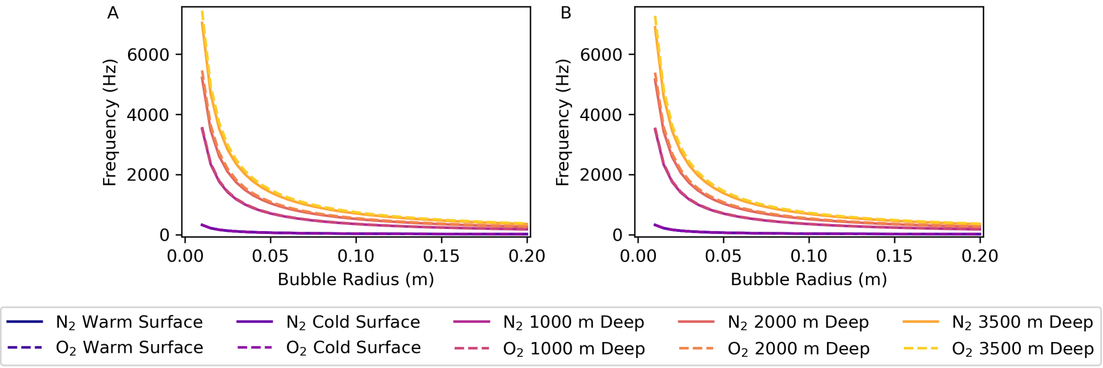

[84]:
```
fig.savefig("Figure1.tiff", bbox_inches="tight")
fig.savefig("Figure1.png", bbox_inches="tight")
```

6. Frequency variation with depth

Define a range of depth values for function evaluation and plotting.

[85]:
```
dvals = 0:10:3500;
```

6.1. Undamped Frequency

Arrays of `fun` values are calculated below for the depths in `dvals` using the interpolated parameters supplied by `valsn2list` and `valso2list`.

[86]:
```
funnd01 = map(d->fun(0.01, valsn2list(d)), dvals);
```

[87]:
```
funnd05 = map(d->fun(0.05, valsn2list(d)), dvals);
```

[88]:
```
funnd1 = map(d->fun(0.1, valsn2list(d)), dvals);
```

[89]:
```
funod01 = map(d->fun(0.01, valso2list(d)), dvals);
```

[90]:
```
funod05 = map(d->fun(0.05, valso2list(d)), dvals);
```

[91]:
```
funod1 = map(d->fun(0.1, valso2list(d)), dvals);
```

6.2. Far-Field Resonant Frequency

Arrays of `fff` values are calculated below for the depths in `dvals` using the interpolated parameters supplied by `valsn2list` and `valso2list`.

[92]:
```
fffnd01 = map(d->fff(0.01, valsn2list(d)), dvals);
```

[93]:
```
fffnd05 = map(d->fff(0.05, valsn2list(d)), dvals);
```

[94]:
```
fffnd1 = map(d->fff(0.1, valsn2list(d)), dvals);
```

[95]:
```
fffod01 = map(d->fff(0.01, valso2list(d)), dvals);
```

[96]:
```
fffod05 = map(d->fff(0.05, valso2list(d)), dvals);
```

[97]:
```
fffod1 = map(d->fff(0.1, valso2list(d)), dvals);
```

6.3. Figure 2

Plot the undamped and far-field resonance frequencies vs. depth for various bubble radii. This is Fig. 2 in the paper.

[98]:
```
heights = [3, 1]
fig = figure(constrained_layout=true, dpi=300, figsize=(7.38, 4))
gs = fig.add_gridspec(2, 2, height_ratios=heights)
ax1 = fig.add_subplot(get(gs, (0, 0)))
ax1.plot(dvals, funnd01, linestyle="-", color=get_cmap("plasma")(0.7), label=L"0.01,␣
  ↪N$_2$")
ax1.plot(dvals, funnd05, linestyle="-", color=get_cmap("plasma")(0.4), label=L"0.05,␣
  ↪N$_2$")
ax1.plot(dvals, funnd1, linestyle="-", color=get_cmap("plasma")(0), label=L"0.1, N$_2$")
ax1.plot(dvals, funod01, linestyle="--", color=get_cmap("plasma")(0.7), label=L"0.01,␣
  ↪O$_2$")
ax1.plot(dvals, funod05, linestyle="--", color=get_cmap("plasma")(0.4), label=L"0.05,␣
  ↪O$_2$")
ax1.plot(dvals, funod1, linestyle="--", color=get_cmap("plasma")(0), label=L"0.1, O$_2$")
ax1.set_ylim([-100, 7600])
ax1.set_xlabel("Depth (m)")
ax1.set_ylabel("Frequency (Hz)")

ax2 = fig.add_subplot(get(gs, (0, 1)))
ax2.plot(dvals, fffnd01, linestyle="-", color=get_cmap("plasma")(0.7))
ax2.plot(dvals, fffnd05, linestyle="-", color=get_cmap("plasma")(0.4))
ax2.plot(dvals, fffnd1, linestyle="-", color=get_cmap("plasma")(0))
ax2.plot(dvals, fffod01, linestyle="--", color=get_cmap("plasma")(0.7))
ax2.plot(dvals, fffod05, linestyle="--", color=get_cmap("plasma")(0.4))
ax2.plot(dvals, fffod1, linestyle="--", color=get_cmap("plasma")(0))
ax2.set_ylim([-100, 7600])
```

```
ax2.set_xlabel("Depth (m)")
ax2.set_ylabel("Frequency (Hz)")

fig.text(0.01, 0.955, "A")
fig.text(0.51, 0.955, "B")
fig.legend(title="Radius (m), Gas", ncol=2, loc="lower center");
```

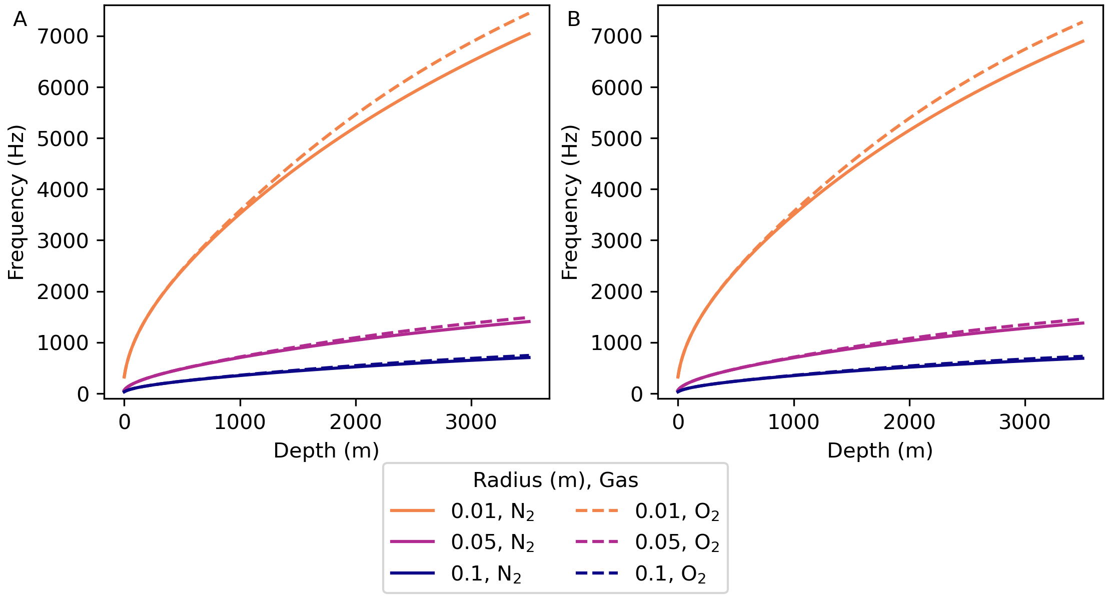

[99]:
```
fig.savefig("Figure2.tiff", bbox_inches="tight")
fig.savefig("Figure2.png", bbox_inches="tight")
```

7. Comparison of Far-Field Resonant Frequency to Minnaert Frequency

In this section we compare the far-field resonant frequency to the Minnaert frequency. First look at how the ratio of the two frequencies varies with bubble radius in the different environments.

[100]:
```
ffmns = map(a1 -> fff(a1, valst("n2", 0)) / fMjl(a1, valst("n2", 0)...), avals);
ffmos = map(a1 -> fff(a1, valst("o2", 0)) / fMjl(a1, valst("o2", 0)...), avals);
ffmncs = map(a1 -> fff(a1, valst("n2", 0, temp="c")) / fMjl(a1, valst("n2", 0, temp="c").
  ↪..), avals);
ffmocs = map(a1 -> fff(a1, valst("o2", 0, temp="c")) / fMjl(a1, valst("o2", 0, temp="c").
  ↪..), avals);
ffmn1k = map(a1 -> fff(a1, valst("n2", 1000)) / fMjl(a1, valst("n2", 1000)...), avals);
ffmo1k = map(a1 -> fff(a1, valst("o2", 1000)) / fMjl(a1, valst("o2", 1000)...), avals);
ffmn2k = map(a1 -> fff(a1, valst("n2", 2000)) / fMjl(a1, valst("n2", 2000)...), avals);
ffmo2k = map(a1 -> fff(a1, valst("o2", 2000)) / fMjl(a1, valst("o2", 2000)...), avals);
```

```
ffmnd = map(a1 -> fff(a1, valst("n2", 3500)) / fMjl(a1, valst("n2", 3500)...), avals);
ffmod = map(a1 -> fff(a1, valst("o2", 3500)) / fMjl(a1, valst("o2", 3500)...), avals);
```

[101]:

```
plot(avals, ffmns, linestyle="-", color=get_cmap("plasma")(0), label=L"N$_2$ Warm␣
 ↪Surface");
plot(avals, ffmos, linestyle="--", color=get_cmap("plasma")(0.1), label=L"O$_2$ Warm␣
 ↪Surface");
plot(avals, ffmncs, linestyle="-", color=get_cmap("plasma")(0.2), label=L"N$_2$ Cold␣
 ↪Surface");
plot(avals, ffmocs, linestyle="--", color=get_cmap("plasma")(0.3), label=L"O$_2$ Cold␣
 ↪Surface");
plot(avals, ffmn1k, linestyle="-", color=get_cmap("plasma")(0.4), label=L"N$_2$ 1000 m␣
 ↪Deep");
plot(avals, ffmo1k, linestyle="--", color=get_cmap("plasma")(0.5), label=L"O$_2$ 1000 m␣
 ↪Deep");
plot(avals, ffmn2k, linestyle="-", color=get_cmap("plasma")(0.6), label=L"N$_2$ 2000 m␣
 ↪Deep");
plot(avals, ffmo2k, linestyle="--", color=get_cmap("plasma")(0.7), label=L"O$_2$ 2000 m␣
 ↪Deep");
plot(avals, ffmnd, linestyle="-", color=get_cmap("plasma")(0.8), label=L"N$_2$ 3500 m␣
 ↪Deep");
plot(avals, ffmod, linestyle="--", color=get_cmap("plasma")(0.9), label=L"O$_2$ 3500 m␣
 ↪Deep");
xlabel("Bubble Radius (m)")
ylabel("Far Field Frequency / Minnaert Frequency")
legend(bbox_to_anchor=[1.05,1],loc=2,borderaxespad=0);
```

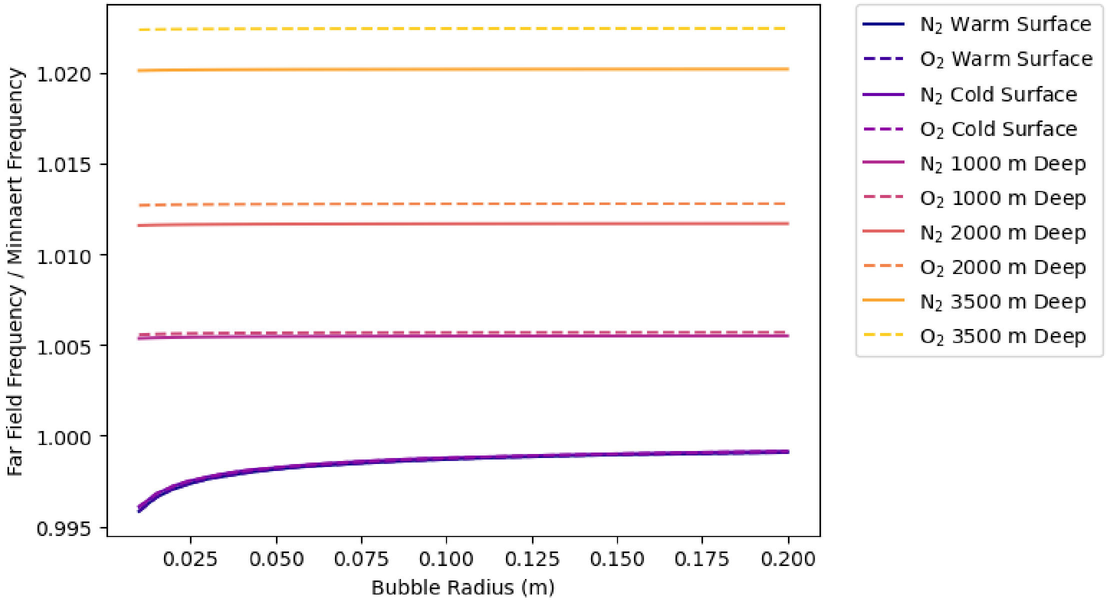

Since the deep water values do not change significantly with bubble radius, use the ratios at a radius of 0.2 m for comparison.

Nitrogen at depths 1000 m, 2000 m, and 3500 m

[102]:
```
ffmn1k[end]
```

[102]: 1.0055089887708542

The far-field resonance frequency is a 0.5509% greater than the Minnaert frequency.

[103]:
```
ffmn2k[end]
```

[103]: 1.0116960118967846

The far-field resonance frequency is a 1.170% greater than the Minnaert frequency.

[104]:
```
ffmnd[end]
```

[104]: 1.020188927947519

The far-field resonance frequency is a 2.019% greater than the Minnaert frequency.

Oxygen at depths 1000 m, 2000 m, and 3500 m

[105]:
```
ffmo1k[end]
```

[105]: 1.005702437168656

The far-field resonance frequency is a 0.5702% greater than the Minnaert frequency.

[106]:
```
ffmo2k[end]
```

[106]: 1.0127875030467226

The far-field resonance frequency is a 1.279% greater than the Minnaert frequency.

[107]:
```
ffmod[end]
```

[107]: 1.0224433087118485

The far-field resonance frequency is a 2.244% greater than the Minnaert frequency.

As seen above, the ratios for nitrogen and oxygen bubbles have the same percent difference (from 1) to one significant figure.

Now look at how the ratio of the frequencies varies with depth for different bubble radii.

[108]:
```
ffmnd01 = map(d -> fff(0.01, valsn2list(d)) / fMjl(0.01, valsn2list(d)...), dvals);
ffmnd05 = map(d -> fff(0.05, valsn2list(d)) / fMjl(0.05, valsn2list(d)...), dvals);
ffmnd1 = map(d -> fff(0.1, valsn2list(d)) / fMjl(0.1, valsn2list(d)...), dvals);
ffmod01 = map(d -> fff(0.01, valso2list(d)) / fMjl(0.01, valso2list(d)...), dvals);
ffmod05 = map(d -> fff(0.05, valso2list(d)) / fMjl(0.05, valso2list(d)...), dvals);
```

```
ffm0d1 = map(d -> fff(0.1, valso2list(d)) / fMjl(0.1, valso2list(d)...), dvals);
```

[109]:
```
plot(dvals, ffmnd01, linestyle="-", color=get_cmap("plasma")(0.7), label=L"0.01, N$_2$")
plot(dvals, ffmnd05, linestyle="-", color=get_cmap("plasma")(0.4), label=L"0.05, N$_2$")
plot(dvals, ffmnd1, linestyle="-", color=get_cmap("plasma")(0), label=L"0.1, N$_2$")
plot(dvals, ffmod01, linestyle=":", color=get_cmap("plasma")(0.7), label=L"0.01, O$_2$")
plot(dvals, ffmod05, linestyle=":", color=get_cmap("plasma")(0.4), label=L"0.05, O$_2$")
plot(dvals, ffm0d1, linestyle=":", color=get_cmap("plasma")(0), label=L"0.1, O$_2$")
xlabel("Depth (m)")
ylabel("Far Field Frequency / Minnaert Frequency")
legend(title="Radius (m), Gas");
```

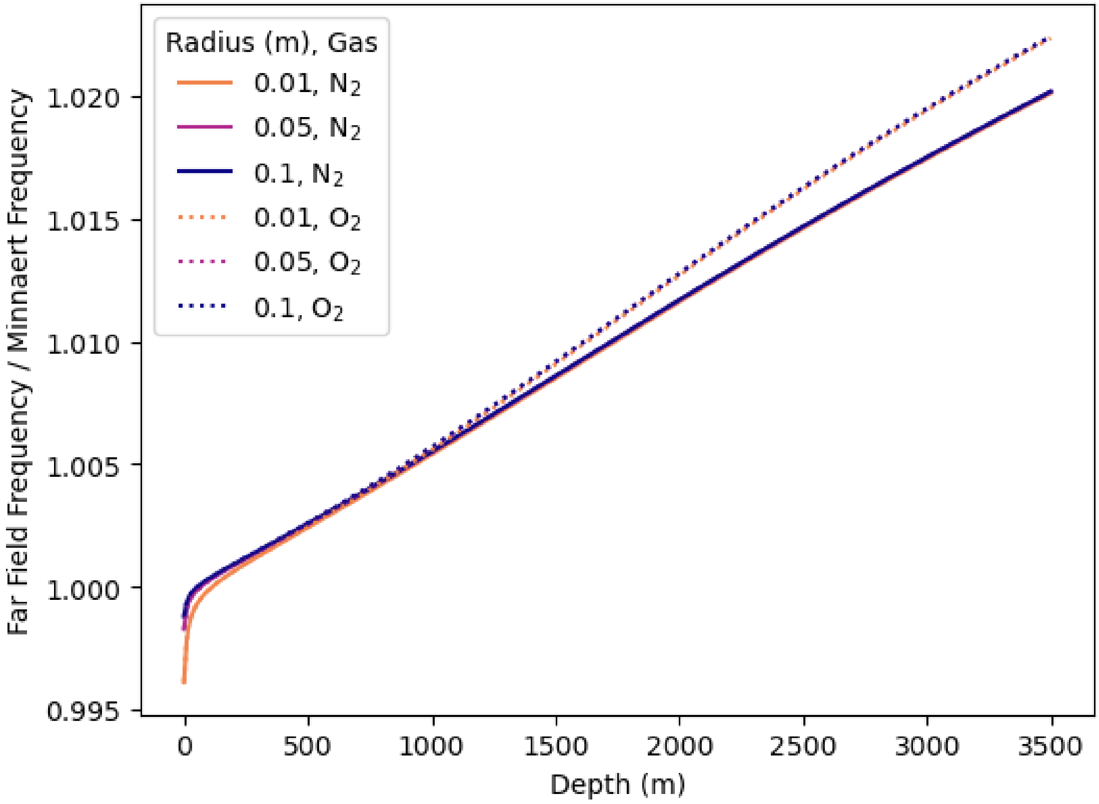

Reformat this graph as Figure 3.

7.1. Figure 3

[110]:
```
fig = figure(dpi=300, figsize=[5.5,3.5])
plot(dvals, ffmnd01, linestyle="-", color=get_cmap("plasma")(0.7), label=L"0.01, N$_2$")
plot(dvals, ffmnd05, linestyle="-", color=get_cmap("plasma")(0.4), label=L"0.05, N$_2$")
plot(dvals, ffmnd1, linestyle="-", color=get_cmap("plasma")(0), label=L"0.1, N$_2$")
plot(dvals, ffmod01, linestyle=":", color=get_cmap("plasma")(0.7), label=L"0.01, O$_2$")
```

```
plot(dvals, ffmod05, linestyle=":", color=get_cmap("plasma")(0.4), label=L"0.05, O$_2$")
plot(dvals, ffm0d1, linestyle=":", color=get_cmap("plasma")(0), label=L"0.1, O$_2$")
xlabel("Depth (m)")
ylabel("Far Field Frequency / Minnaert Frequency")
legend(title="Radius (m), Gas", ncol=2, loc="lower right");
```

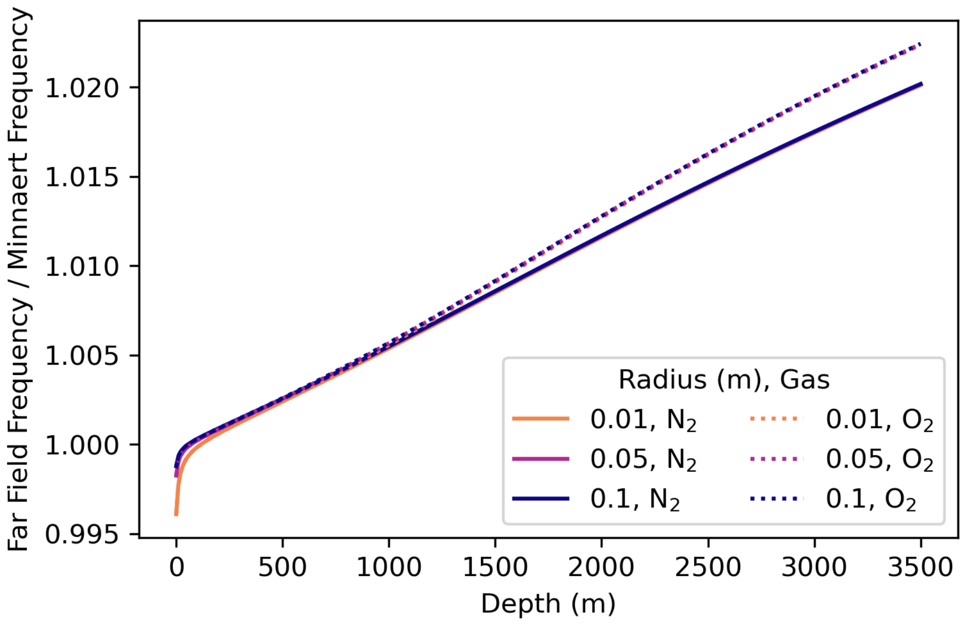

[111]:
```
fig.savefig("Figure3.tiff", bbox_inches="tight")
fig.savefig("Figure3.png", bbox_inches="tight")
```

8. Normalized Parameters

In this section we investigate the scaling properties of the scattering coefficient and the resonant frequencies. We normalize the scattering coefficient by dividing it by the bubble surface area $4\pi a^2$, and we normalize the frequency values by dividing by f_{nat}.

Create a function for the normalized scattering coefficient.

[112]:
```
σsjlnorm(a1, ρ, pw, μ, σ, c, dd, γ, f1) = σsjl(a1, ρ, pw, μ, σ, c, dd, γ, f1)/(4*π*a1^2)
```

[112]:
```
σsjlnorm (generic function with 1 method)
```

Produce a list of normalized frequency values to use for graphs. Use 1000 points to get sufficient resolution near peaks of σ_s'.

[113]:
```
fnorm = exp10.(range(log10(0.01), stop=log10(5), length=1000));
```

8.1. Nitrogen

Now produce arrays of normalized scattering coefficient values for each normalized frequency value in `fnorm`. Since the `σsjlnorm` function needs the frequency value instead of the normalized frequency value (because it was defined using the non-normalized scattering coefficient function), we multiply the normalized frequency values by the undamped frequency in the arguments of `σsjlnorm`.

8.1.1. Warm Surface

[114]:
```
σsns1 = map(f1->σsjlnorm(0.1, valst("n2", 0)..., f1),
    fun(0.1, valst("n2", 0)) .* fnorm);
```

[115]:
```
σsns05 = map(f1->σsjlnorm(0.05, valst("n2", 0)..., f1),
    fun(0.05, valst("n2", 0)) .* fnorm);
```

[116]:
```
σsns01 = map(f1->σsjlnorm(0.01, valst("n2", 0)..., f1),
    fun(0.01, valst("n2", 0)) .* fnorm);
```

Graph the normalized scattering coefficient vs. normalized frequency for these three bubble radii.

[117]:
```
fig, ax = subplots()
ax.semilogx(fnorm, σsns01, linestyle="-", color=get_cmap("plasma")(0))
ax.semilogx(fnorm, σsns05, linestyle="--", color=get_cmap("plasma")(0.5))
ax.semilogx(fnorm, σsns1, linestyle=":", color=get_cmap("plasma")(0.8))
ax.set_xlabel("Normalized Frequency")
ax.set_ylabel(L"${\sigma_s}'$");
```

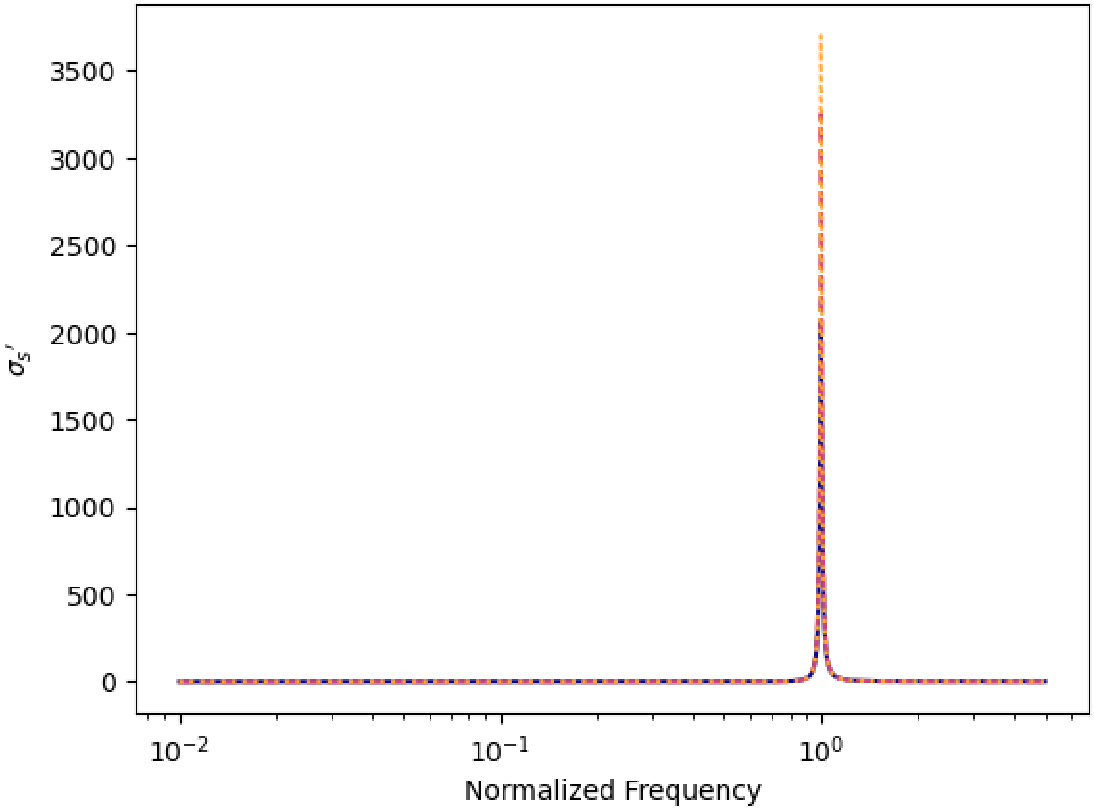

This is Fig. 3(a) in the paper.

Get the maximum values for comparison.

```
[118]: maximum((maximum(σsns1), maximum(σsns05), maximum(σsns01)))
```

```
[118]: 3700.0589172766777
```

8.1.2. Cold Surface

```
[119]: σsncs1 = map(f1->σsjlnorm(0.1, valst("n2", 0, temp="cold")..., f1), fun(0.1, valst("n2",␣
         ↪0,
                    temp="cold")) .* fnorm);
```

```
[120]: σsncs05 = map(f1->σsjlnorm(0.05, valst("n2", 0, temp="cold")..., f1), fun(0.05,␣
         ↪valst("n2", 0,
                    temp="cold")) .* fnorm);
```

```
[121]: σsncs01 = map(f1->σsjlnorm(0.01, valst("n2", 0, temp="cold")..., f1), fun(0.01,␣
         ↪valst("n2", 0,
                    temp="cold")) .* fnorm);
```

```
[122]: semilogx(fnorm, σsncs01, linestyle="-", color=get_cmap("plasma")(0))
       semilogx(fnorm, σsncs05, linestyle="--", color=get_cmap("plasma")(0.5))
       semilogx(fnorm, σsncs1, linestyle=":", color=get_cmap("plasma")(0.8))
       xlabel("Normalized Frequency")
       ylabel(L"${\sigma_s}'$");
```

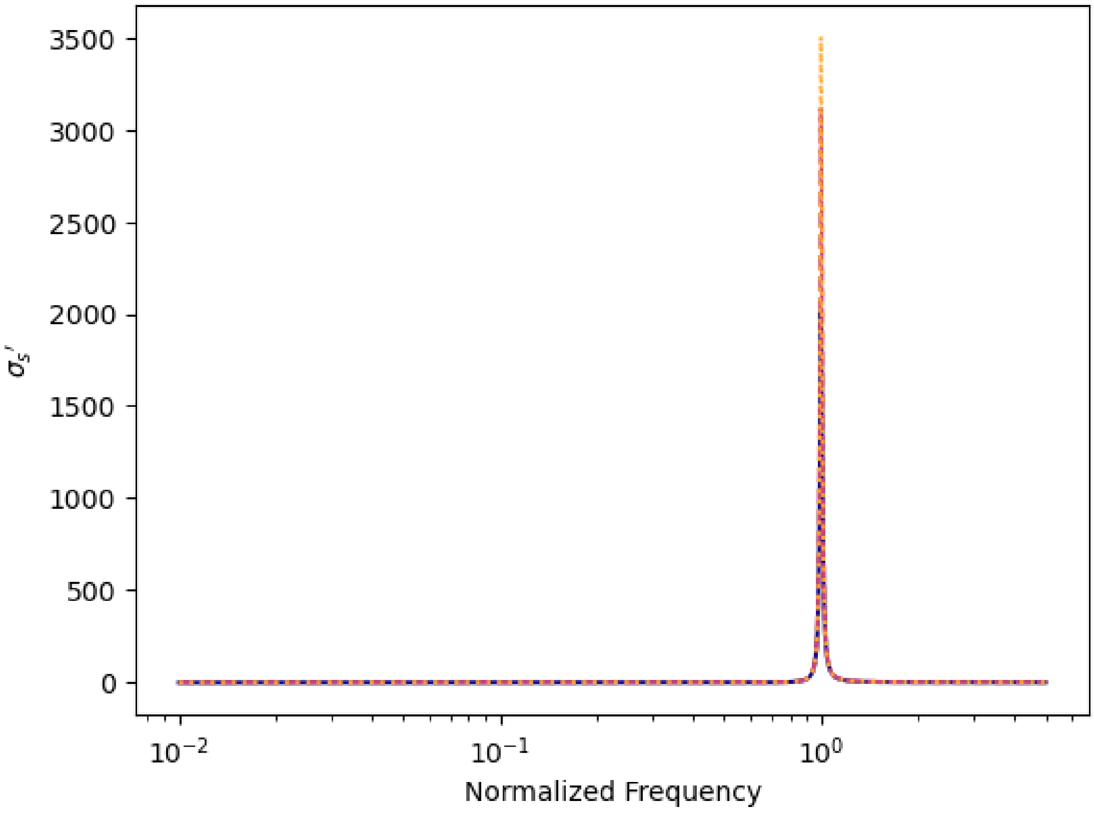

This is Fig. 3(c) in the paper.

Get the maximum values for comparison.

```
[123]: mcsn = maximum((maximum(σsncs1), maximum(σsncs05), maximum(σsncs01)))
```

```
[123]: 3503.349100567182
```

8.1.3. Depth 1000 m

```
[124]: σsn1k1 = map(f1->σsjlnorm(0.1, valst("n2", 1000, temp="cold")..., f1), fun(0.1,␣
        ↪valst("n2", 1000,
                   temp="cold")) .* fnorm);
```

[125]:
```
σsn1k05 = map(f1->σsjlnorm(0.05, valst("n2", 1000, temp="cold")..., f1), fun(0.05,␣
↪valst("n2", 1000,
            temp="cold")) .* fnorm);
```

[126]:
```
σsn1k01 = map(f1->σsjlnorm(0.01, valst("n2", 1000, temp="cold")..., f1), fun(0.01,␣
↪valst("n2", 1000,
            temp="cold")) .* fnorm);
```

[127]:
```
semilogx(fnorm, σsn1k01, linestyle="-", color=get_cmap("plasma")(0))
semilogx(fnorm, σsn1k05, linestyle="--", color=get_cmap("plasma")(0.5))
semilogx(fnorm, σsn1k1, linestyle=":", color=get_cmap("plasma")(0.8))
xlabel("Normalized Frequency")
ylabel(L"${\sigma_s}'$");
```

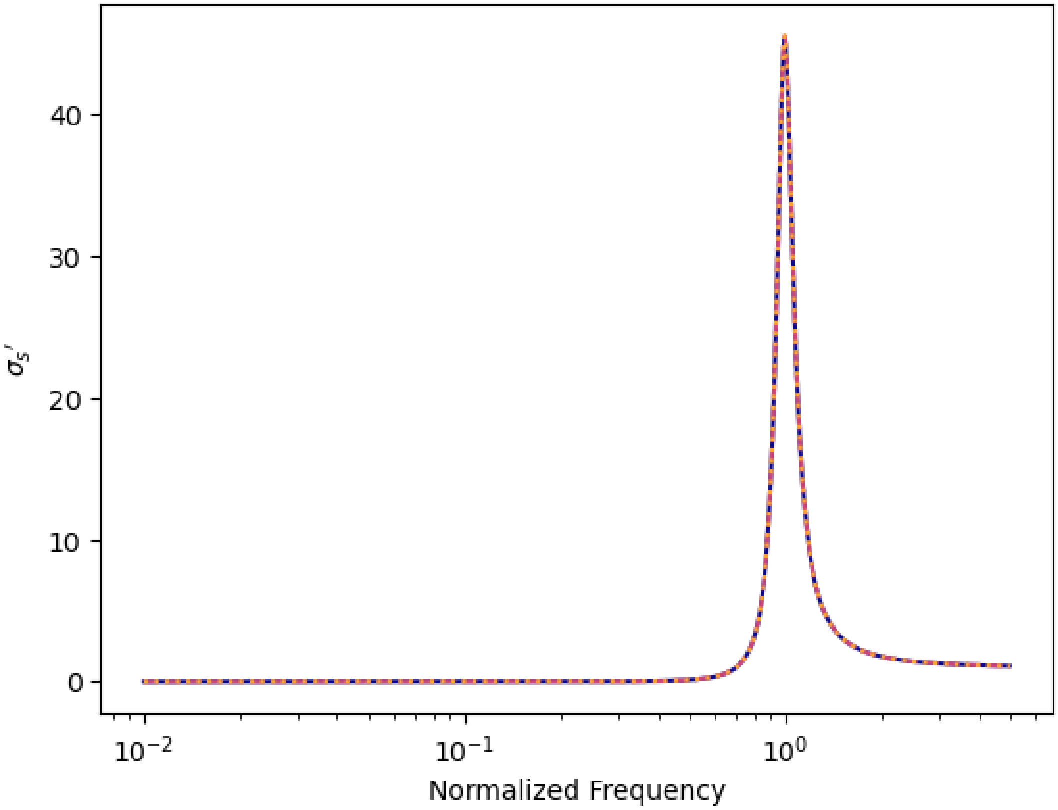

This is Fig. 3(c) in the paper.

Get the maximum values for comparison.

[128]:
```
maximum((maximum(σsn1k1), maximum(σsn1k05), maximum(σsn1k01)))
```

[128]: 45.57401941932281

[129]:
```
mcsn / maximum((maximum(σsn1k1), maximum(σsn1k05), maximum(σsn1k01)))
```

[129]: 76.87162873068436

8.1.4. Depth 2000 m

[130]:
```
σsn2k1 = map(f1->σsjlnorm(0.1, valst("n2", 2000, temp="cold")..., f1), fun(0.1,␣
↪valst("n2", 2000,
            temp="cold")) .* fnorm);
```

[131]:
```
σsn2k05 = map(f1->σsjlnorm(0.05, valst("n2", 2000, temp="cold")..., f1), fun(0.05,␣
↪valst("n2",2000,
            temp="cold")) .* fnorm);
```

[132]:
```
σsn2k01 = map(f1->σsjlnorm(0.01, valst("n2", 2000, temp="cold")..., f1), fun(0.01,␣
↪valst("n2", 2000,
            temp="cold")) .* fnorm);
```

[133]:
```
semilogx(fnorm, σsn2k01, linestyle="-", color=get_cmap("plasma")(0))
semilogx(fnorm, σsn2k05, linestyle="--", color=get_cmap("plasma")(0.5))
semilogx(fnorm, σsn2k1, linestyle=":", color=get_cmap("plasma")(0.8))
xlabel("Normalized Frequency")
ylabel(L"${\sigma_s}'$");
```

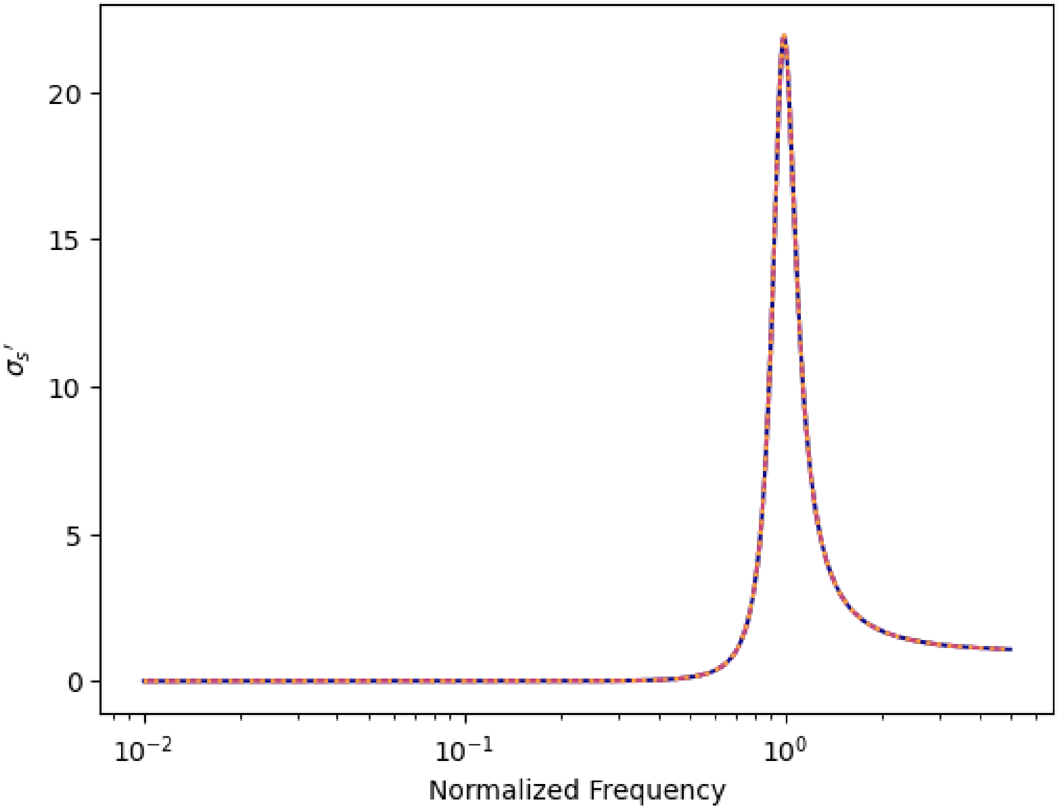

This is Fig. 3(c) in the paper.

Get the maximum values for comparison.

[134]:
```
maximum((maximum(σsn2k1), maximum(σsn2k05), maximum(σsn2k01)))
```

[134]: 21.924562570677537

[135]:
```
mcsn / maximum((maximum(σsn2k1), maximum(σsn2k05), maximum(σsn2k01)))
```

[135]: 159.79106033580115

8.1.5. Deep

[136]:
```
σsnd1 = map(f1->σsjlnorm(0.1, valst("n2", 3500)..., f1), fun(0.1, valst("n2", 3500)) .*
    fnorm);
```

[137]:
```
σsnd05 = map(f1->σsjlnorm(0.05, valst("n2", 3500)..., f1), fun(0.05, valst("n2", 3500)) .
  ↪*
    fnorm);
```

[138]:
```
σsnd01 = map(f1->σsjlnorm(0.01, valst("n2", 3500)..., f1), fun(0.01, valst("n2", 3500)) .
  ↪*
    fnorm);
```

[139]:
```
semilogx(fnorm, σsnd01, linestyle="-", color=get_cmap("plasma")(0))
semilogx(fnorm, σsnd05, linestyle="--", color=get_cmap("plasma")(0.5))
semilogx(fnorm, σsnd1, linestyle=":", color=get_cmap("plasma")(0.8))
xlabel("Normalized Frequency")
ylabel(L"${\sigma_s}'$");
```

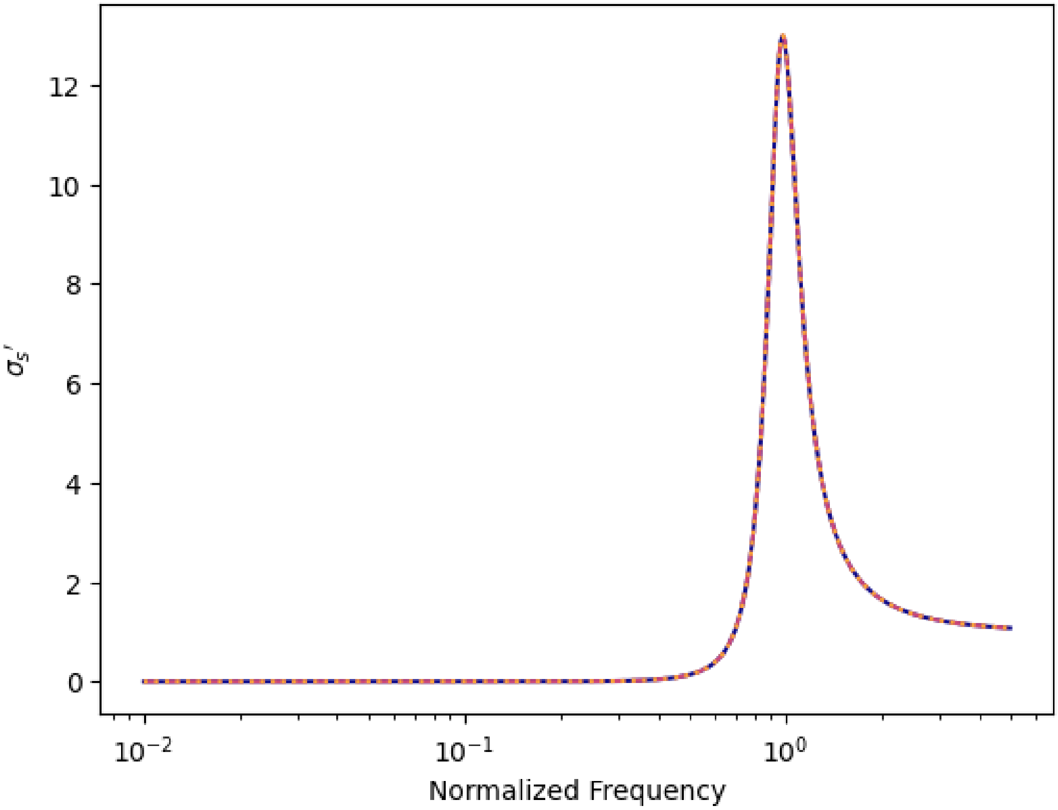

This is Fig. 3(e) in the paper.

Get the maximum values for comparison.

[140]:
```
maximum((maximum(σsnd1), maximum(σsnd05), maximum(σsnd01)))
```

[140]: 12.994265697172283

[141]:
```
mcsn / maximum((maximum(σsnd1), maximum(σsnd05), maximum(σsnd01)))
```

[141]: 269.60731619714034

8.2. Oxygen

Repeat the same normalized scattering coefficient vs. normalized frequency calculations for oxygen bubbles.

8.2.1. Warm Surface

[142]:
```
σsos1 = map(f1->σsjlnorm(0.1, valst("o2", 0)..., f1), fun(0.1, valst("o2", 0)) .*
    fnorm);
```

[143]:
```
σsos05 = map(f1->σsjlnorm(0.05, valst("o2", 0)..., f1), fun(0.05, valst("o2", 0)) .*
    fnorm);
```

[144]:
```
σsos01 = map(f1->σsjlnorm(0.01, valst("o2", 0)..., f1), fun(0.01, valst("o2", 0)) .*
    fnorm);
```

[145]:
```
semilogx(fnorm, σsos01, linestyle="-", color=get_cmap("plasma")(0))
semilogx(fnorm, σsos05, linestyle="--", color=get_cmap("plasma")(0.5))
semilogx(fnorm, σsos1, linestyle=":", color=get_cmap("plasma")(0.8))
xlabel("Normalized Frequency")
ylabel(L"${\sigma_s}'$");
```

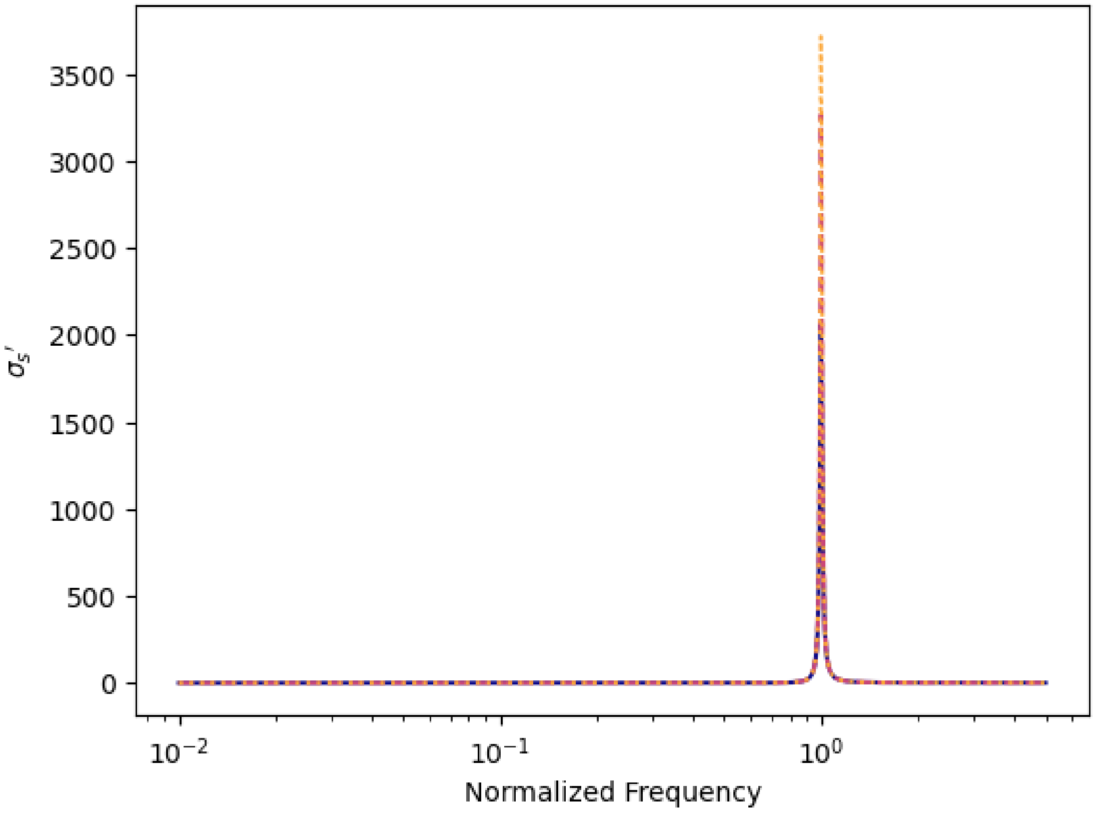

This is Fig. 3(b) in the paper.

Get the maximum values for comparison.

[146]:
```
maximum((maximum(σsos1), maximum(σsos05), maximum(σsos01)))
```

[146]: 3713.6377935132464

8.2.2. Cold Surface

[147]:
```
σsocs1 = map(f1->σsjlnorm(0.1, valst("o2", 0, temp="c")..., f1), fun(0.1, valst("o2", 0,
            temp="c")) .* fnorm);
```

[148]:
```
σsocs05 = map(f1->σsjlnorm(0.05, valst("o2", 0, temp="c")..., f1), fun(0.05, valst("o2",␣
 ↪0,
            temp="c")) .* fnorm);
```

[149]:
```
σsocs01 = map(f1->σsjlnorm(0.01, valst("o2", 0, temp="c")..., f1), fun(0.01, valst("o2",␣
 ↪0,
            temp="c")) .* fnorm);
```

[150]:
```
semilogx(fnorm, σsocs01, linestyle="-", color=get_cmap("plasma")(0))
semilogx(fnorm, σsocs05, linestyle="--", color=get_cmap("plasma")(0.5))
semilogx(fnorm, σsocs1, linestyle=":", color=get_cmap("plasma")(0.8))
xlabel("Normalized Frequency")
ylabel(L"${\sigma_s}'$");
```

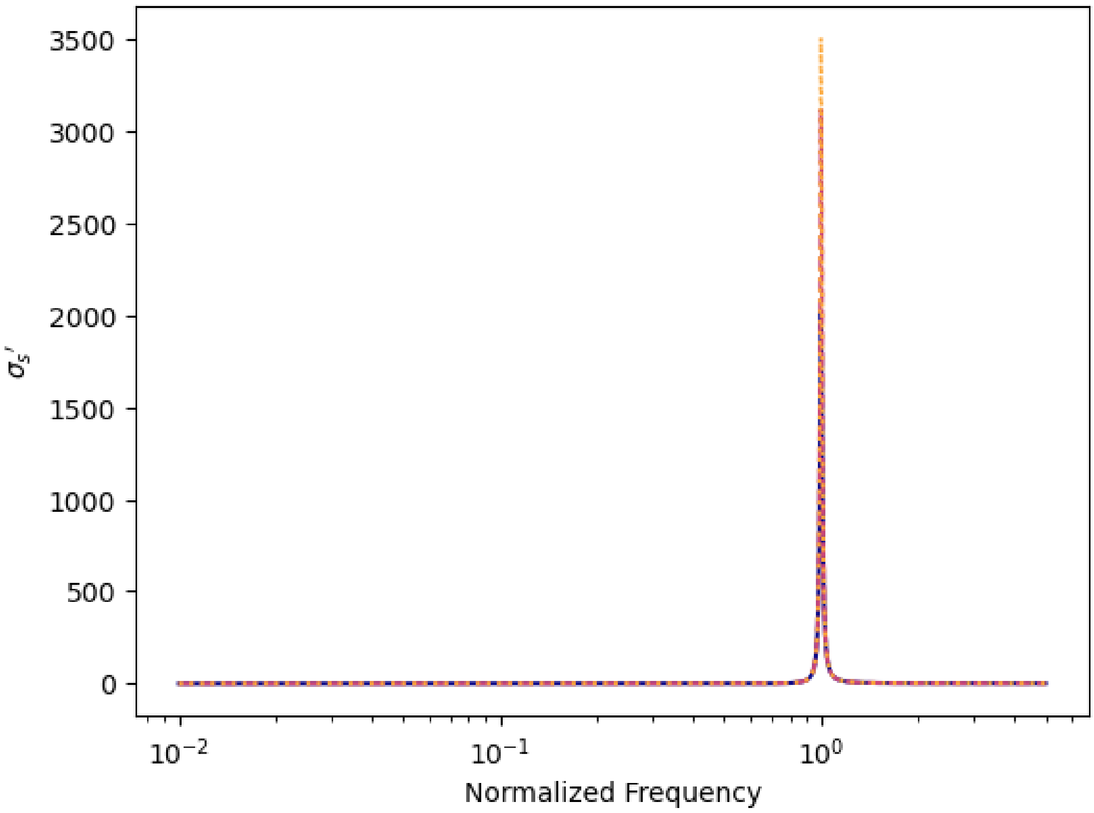

This is Fig. 3(d) in the paper.

Get the maximum values for comparison.

[151]:
```
mcso = maximum((maximum(σsocs1), maximum(σsocs05), maximum(σsocs01)))
```

[151]: 3507.7743920676457

8.2.3. Depth 1000 m

[152]:
```
σso1k1 = map(f1->σsjlnorm(0.1, valst("o2", 1000, temp="c")..., f1), fun(0.1, valst("o2",␣
↪1000,
            temp="c")) .* fnorm);
```

[153]:
```
σso1k05 = map(f1->σsjlnorm(0.05, valst("o2", 1000, temp="c")..., f1), fun(0.05,␣
↪valst("o2", 1000,
            temp="c")) .* fnorm);
```

[154]:
```
σso1k01 = map(f1->σsjlnorm(0.01, valst("o2", 1000, temp="c")..., f1), fun(0.01,␣
↪valst("o2", 1000,
            temp="c")) .* fnorm);
```

[155]:
```
semilogx(fnorm, σso1k01, linestyle="-", color=get_cmap("plasma")(0))
semilogx(fnorm, σso1k05, linestyle="--", color=get_cmap("plasma")(0.5))
semilogx(fnorm, σso1k1, linestyle=":", color=get_cmap("plasma")(0.8))
xlabel("Normalized Frequency")
ylabel(L"${\sigma_s}'$");
```

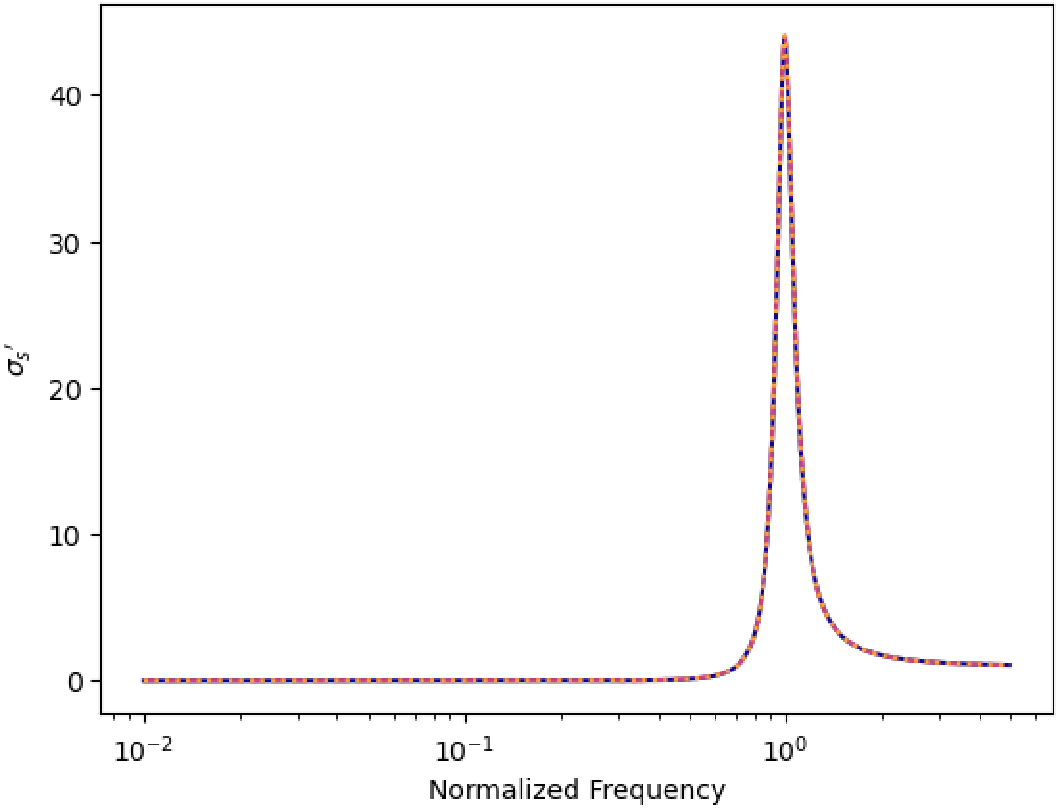

This is Fig. 3(d) in the paper.

Get the maximum values for comparison.

[156]:
```
maximum((maximum(σso1k1), maximum(σso1k05), maximum(σso1k01)))
```

[156]: 44.06041021609415

[157]:
```
mcso / maximum((maximum(σso1k1), maximum(σso1k05), maximum(σso1k01)))
```

[157]: 79.61284007261342

8.2.4. Depth 2000 m

[158]:
```
σso2k1 = map(f1->σsjlnorm(0.1, valst("o2", 2000, temp="c")..., f1), fun(0.1, valst("o2",␣
↪2000,
            temp="c")) .* fnorm);
```

[159]:
```
σso2k05 = map(f1->σsjlnorm(0.05, valst("o2", 2000, temp="c")..., f1), fun(0.05,␣
↪valst("o2", 2000,
            temp="c")) .* fnorm);
```

[160]:
```
σso2k01 = map(f1->σsjlnorm(0.01, valst("o2", 2000, temp="c")..., f1), fun(0.01,␣
↪valst("o2", 2000,
            temp="c")) .* fnorm);
```

[161]:
```
semilogx(fnorm, σso2k01, linestyle="-", color=get_cmap("plasma")(0))
semilogx(fnorm, σso2k05, linestyle="--", color=get_cmap("plasma")(0.5))
semilogx(fnorm, σso2k1, linestyle=":", color=get_cmap("plasma")(0.8))
xlabel("Normalized Frequency")
ylabel(L"${\sigma_s}'$");
```

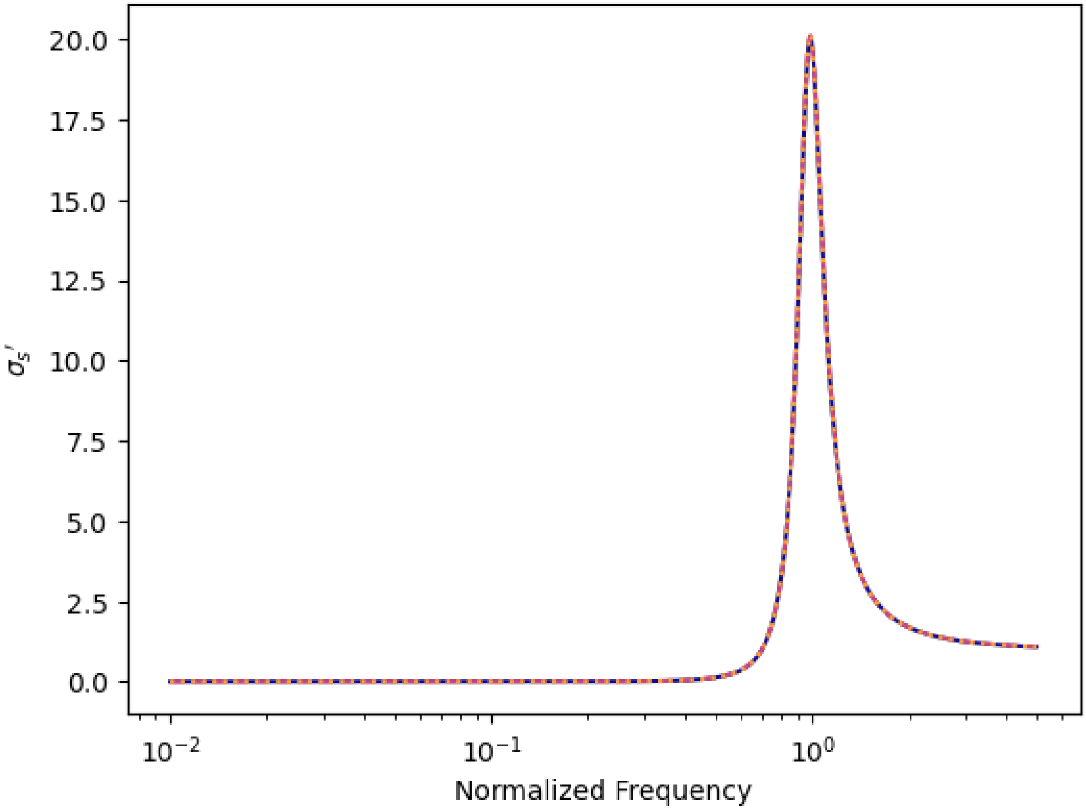

This is Fig. 3(d) in the paper.

Get the maximum values for comparison.

[162]:
```
maximum((maximum(σso2k1), maximum(σso2k05), maximum(σso2k01)))
```

[162]: 20.11480737475691

[163]:
```
mcso / maximum((maximum(σso2k1), maximum(σso2k05), maximum(σso2k01)))
```

[163]: 174.38767007382478

8.2.3. Deep

[164]:
```
σsod1 = map(f1->σsjlnorm(0.1, valst("o2", 3500)..., f1), fun(0.1, valst("o2", 3500)) .*
    fnorm);
```

[165]:
```
σsod05 = map(f1->σsjlnorm(0.05, valst("o2", 3500)..., f1), fun(0.05, valst("o2", 3500)) .
  ↪*
    fnorm);
```

[166]:
```
σsod01 = map(f1->σsjlnorm(0.01, valst("o2", 3500)..., f1), fun(0.01, valst("o2", 3500)) .
↪*
    fnorm);
```

[167]:
```
semilogx(fnorm, σsod01, linestyle="-", color=get_cmap("plasma")(0))
semilogx(fnorm, σsod05, linestyle="--", color=get_cmap("plasma")(0.5))
semilogx(fnorm, σsod1, linestyle=":", color=get_cmap("plasma")(0.8))
xlabel("Normalized Frequency")
ylabel(L"${\sigma_s}'$");
```

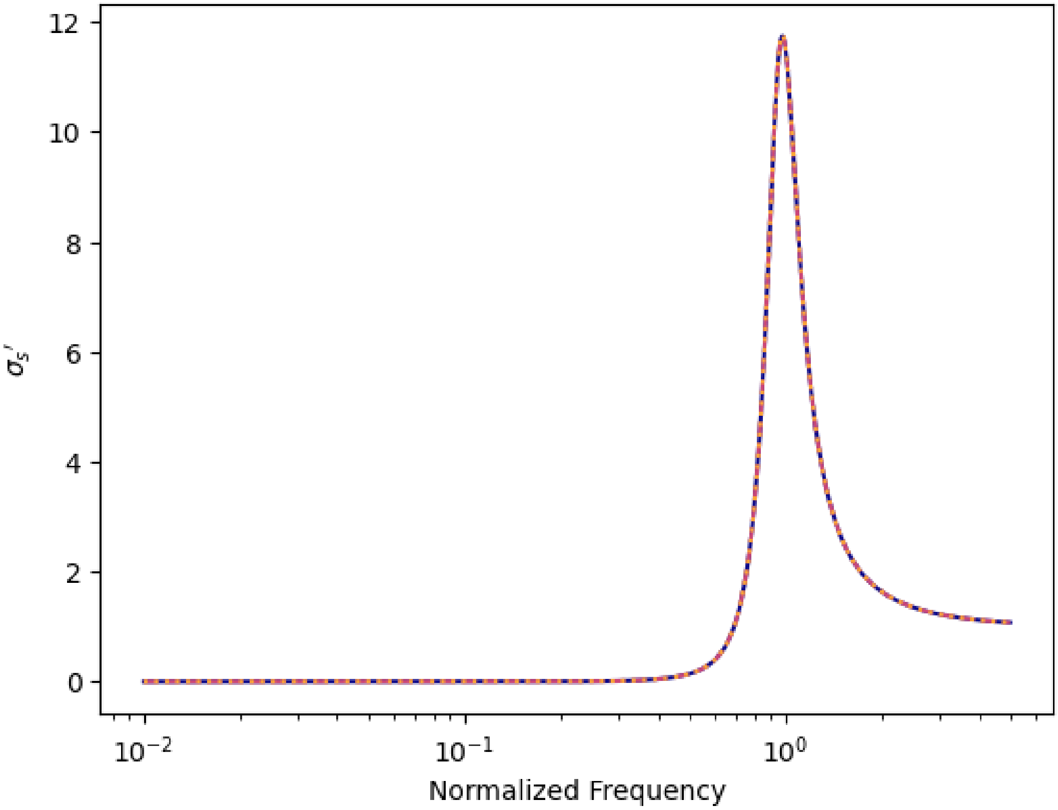

This is Fig. 3(f) in the paper.

Get the maximum values for comparison.

[168]:
```
maximum((maximum(σsod1), maximum(σsod05), maximum(σsod01)))
```

[168]: 11.749551352830728

[169]:
```
mcso / maximum((maximum(σsod1), maximum(σsod05), maximum(σsod01)))
```

[169]: 298.5453900946222

8.3. Figure 4

Combine these graphs into Fig. 4.

[170]:
```
yts = (0, 2000, 4000)
ytej = (0, 20, 40)
ytd = (0, 3, 6, 9, 12)
heights = [2, 2, 2, 2, 2, 1.5]



fig = figure(constrained_layout=true, dpi=300, figsize=(7.38,5.21))
gs = fig.add_gridspec(6, 4, height_ratios=heights)
ax1 = fig.add_subplot(get(gs, (0, slice(0, 2))))
ax1.semilogx(fnorm, σsns01, linestyle="-", color=get_cmap("plasma")(0), label="0.01")
ax1.semilogx(fnorm, σsns05, linestyle="--", color=get_cmap("plasma")(0.5), label="0.05")
ax1.semilogx(fnorm, σsns1, linestyle=":", color=get_cmap("plasma")(0.8), label="0.10")
#ax1.set_xlabel("Normalized Frequency")
ax1.set_ylabel(L"${\sigma_s}'$")
ax1.set_xticklabels(())
ax1.set_ylim((-200, 4100))
ax1.set_yticks(yts)

ax2 = fig.add_subplot(get(gs, (0, slice(2, 4))))
ax2.semilogx(fnorm, σsos01, linestyle="-", color=get_cmap("plasma")(0))
ax2.semilogx(fnorm, σsos05, linestyle="--", color=get_cmap("plasma")(0.5))
ax2.semilogx(fnorm, σsos1, linestyle=":", color=get_cmap("plasma")(0.8))
#ax2.set_xlabel("Normalized Frequency")
#ax2.set_ylabel(L"${\sigma_s}'$")
ax2.set_xticklabels(())
ax2.set_ylim((-200, 4100))
ax2.set_yticks(yts)
ax2.set_yticklabels(())

ax3 = fig.add_subplot(get(gs, (1, slice(0, 2))))
ax3.semilogx(fnorm, σsncs01, linestyle="-", color=get_cmap("plasma")(0))
ax3.semilogx(fnorm, σsncs05, linestyle="--", color=get_cmap("plasma")(0.5))
ax3.semilogx(fnorm, σsncs1, linestyle=":", color=get_cmap("plasma")(0.8))
#ax3.set_xlabel("Normalized Frequency")
ax3.set_ylabel(L"${\sigma_s}'$");
ax3.set_xticklabels(())
ax3.set_ylim((-200, 4100))
ax3.set_yticks(yts)

ax4 = fig.add_subplot(get(gs, (1, slice(2, 4))))
ax4.semilogx(fnorm, σsocs01, linestyle="-", color=get_cmap("plasma")(0))
ax4.semilogx(fnorm, σsocs05, linestyle="--", color=get_cmap("plasma")(0.5))
ax4.semilogx(fnorm, σsocs1, linestyle=":", color=get_cmap("plasma")(0.8))
#ax4.set_xlabel("Normalized Frequency")
```

```
#ax4.set_ylabel(L"${\sigma_s}'$");
ax4.set_xticklabels(())
ax4.set_ylim((-200, 4100))
ax4.set_yticks(yts)
ax4.set_yticklabels(())

ax5 = fig.add_subplot(get(gs, (2, slice(0, 2))))
ax5.semilogx(fnorm, σsn1k01, linestyle="-", color=get_cmap("plasma")(0))
ax5.semilogx(fnorm, σsn1k05, linestyle="--", color=get_cmap("plasma")(0.5))
ax5.semilogx(fnorm, σsn1k1, linestyle=":", color=get_cmap("plasma")(0.8))
#ax5.set_xlabel("Normalized Frequency")
ax5.set_ylabel(L"${\sigma_s}'$")
ax5.set_ylim((-2, 49))
ax5.set_yticks(ytej)


ax6 = fig.add_subplot(get(gs, (2, slice(2, 4))))
ax6.semilogx(fnorm, σso1k01, linestyle="-", color=get_cmap("plasma")(0))
ax6.semilogx(fnorm, σso1k05, linestyle="--", color=get_cmap("plasma")(0.5))
ax6.semilogx(fnorm, σso1k1, linestyle=":", color=get_cmap("plasma")(0.8))
#ax6.set_xlabel("Normalized Frequency")
#ax6.set_ylabel(L"${\sigma_s}'$")
ax6.set_ylim((-2, 49))
ax6.set_yticks(ytej)
ax6.set_yticklabels(())

ax7 = fig.add_subplot(get(gs, (3, slice(0, 2))))
ax7.semilogx(fnorm, σsn2k01, linestyle="-", color=get_cmap("plasma")(0))
ax7.semilogx(fnorm, σsn2k05, linestyle="--", color=get_cmap("plasma")(0.5))
ax7.semilogx(fnorm, σsn2k1, linestyle=":", color=get_cmap("plasma")(0.8))
#ax7.set_xlabel("Normalized Frequency")
ax7.set_ylabel(L"${\sigma_s}'$")
ax7.set_ylim((-2, 49))
ax7.set_yticks(ytej)

ax8 = fig.add_subplot(get(gs, (3, slice(2, 4))))
ax8.semilogx(fnorm, σso2k01, linestyle="-", color=get_cmap("plasma")(0))
ax8.semilogx(fnorm, σso2k05, linestyle="--", color=get_cmap("plasma")(0.5))
ax8.semilogx(fnorm, σso2k1, linestyle=":", color=get_cmap("plasma")(0.8))
#ax8.set_xlabel("Normalized Frequency")
#ax8.set_ylabel(L"${\sigma_s}'$")
ax8.set_ylim((-2, 49))
ax8.set_yticks(ytej)
ax8.set_yticklabels(())

ax9 = fig.add_subplot(get(gs, (4, slice(0, 2))))
ax9.semilogx(fnorm, σsnd01, linestyle="-", color=get_cmap("plasma")(0))
```

```
ax9.semilogx(fnorm, σsnd05, linestyle="--", color=get_cmap("plasma")(0.5))
ax9.semilogx(fnorm, σsnd1, linestyle=":", color=get_cmap("plasma")(0.8))
ax9.set_xlabel("Normalized Frequency")
ax9.set_ylabel(L"${\sigma_s}'$")
ax9.set_ylim((-2, 49))
ax9.set_yticks(ytej)

ax10 = fig.add_subplot(get(gs, (4, slice(2, 4))))
ax10.semilogx(fnorm, σsod01, linestyle="-", color=get_cmap("plasma")(0))
ax10.semilogx(fnorm, σsod05, linestyle="--", color=get_cmap("plasma")(0.5))
ax10.semilogx(fnorm, σsod1, linestyle=":", color=get_cmap("plasma")(0.8))
ax10.set_xlabel("Normalized Frequency")
#ax10.set_ylabel(L"${\sigma_s}'$")
ax10.set_ylim((-2, 49))
ax10.set_yticks(ytej)
ax10.set_yticklabels(())

ax1.get_shared_x_axes().join(ax1, ax3, ax5, ax7, ax9)
ax2.get_shared_x_axes().join(ax2, ax4, ax6, ax8, ax10)
ax1.get_shared_y_axes().join(ax1, ax2)
ax3.get_shared_y_axes().join(ax3, ax4)
ax5.get_shared_y_axes().join(ax5, ax6)
ax7.get_shared_y_axes().join(ax7, ax8)
ax9.get_shared_y_axes().join(ax9, ax10)

fig.text(0.095, 0.955, "A")
fig.text(0.56, 0.955, "B")
fig.text(0.095, 0.795, "C")
fig.text(0.56, 0.795, "D")
fig.text(0.095, 0.645, "E")
fig.text(0.56, 0.645, "F")
fig.text(0.095, 0.46, "G")
fig.text(0.56, 0.46, "H")
fig.text(0.095, 0.275, "I")
fig.text(0.56, 0.275, "J")
fig.legend(title="Bubble Radius (m)", ncol=3, loc="lower center");
```

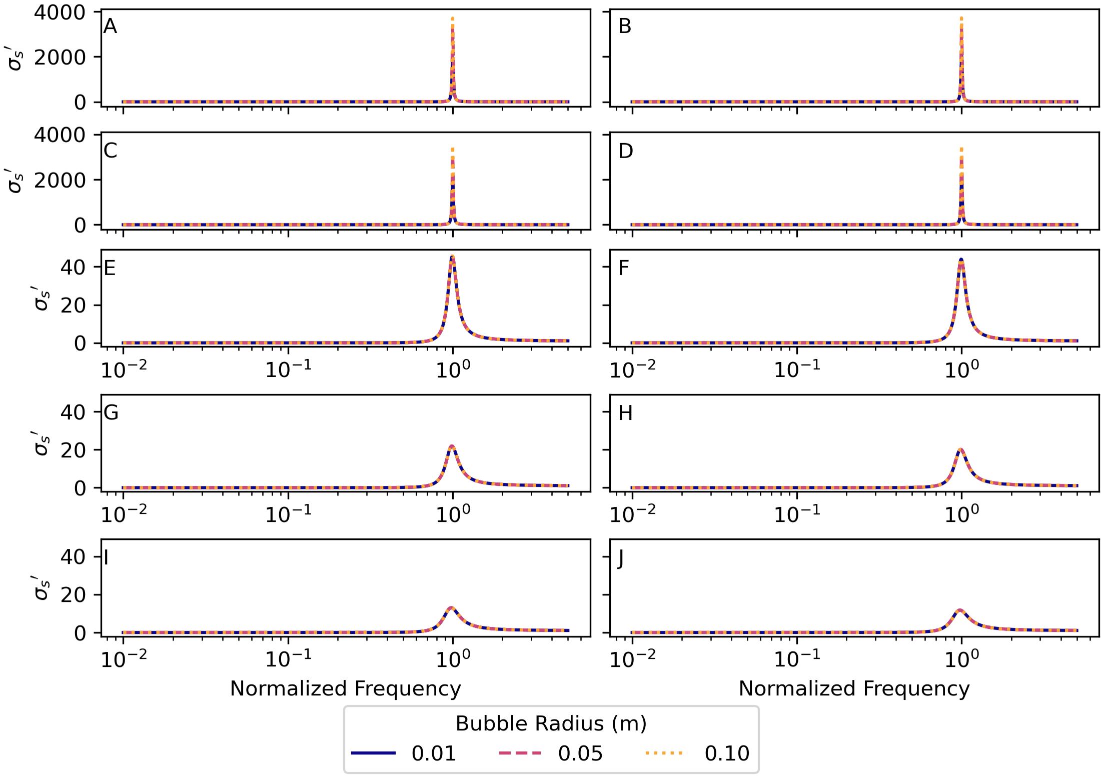


```
[171]: fig.savefig("Figure4.tiff", bbox_inches="tight")
       fig.savefig("Figure4.png", bbox_inches="tight")
```

8.4. Variation of normalized far-field resonance frequency

To investigate the variation of the normalized far-field resonance frequency with bubble, we compare its value for 10 equally-spaced bubble radii from 0.01 m to 0.20 m.

Define a range of 20 bubble radii from 0.01 m to 0.20 m. Also define an shorter array with a few selected radii in that range.

```
[172]: alistlong = 0.01:0.01:0.20
       alistff = [0.01, 0.05, 0.1, 0.15, 0.2];
```

```
[173]: alistlong[1], alistlong[5], alistlong[10], alistlong[15], alistlong[20]
```

```
[173]: (0.01, 0.05, 0.1, 0.15, 0.2)
```

This will pick out the `alistff` values from an `alistlog` indexed calculation.

```
[174]: nff = [1, 5, 10, 15, 20]
       getindex(alistlong, nff)
```

[174]: 5-element Vector{Float64}:
```
 0.01
 0.05
 0.1
 0.15
 0.2
```

Now produce arrays of far-field resonance frequencies and undamped resonance frequencies for each bubble radius in the two lists defined above.

8.4.1. Warm Surface N2

[175]:
```
ffflistl = map(a->fff(a, valst("n2",0)), alistlong);
```

[176]:
```
ffflist = map(a->fff(a, valst("n2",0), 10, 330), alistff);
```

[177]:
```
funlistl = map(a->fun(a, valst("n2",0)), alistlong);
```

[178]:
```
funlist = map(a->fun(a, valst("n2",0)), alistff);
```

Now divide the terms in the far-field resonance frequency array by the terms in the undamped resonance frequency array to produce an array of normalized far-field resonance frequency values for each of the bubble radii.

[179]:
```
fffnormlistl = ffflistl ./ funlistl
```

[179]: 20-element Vector{Float64}:
```
 1.0000422739278316
 1.0000080613994453
 0.9999951598464357
 0.9999881429518314
 0.9999836461644972
 0.9999804793230586
 0.9999781089720733
 0.9999762570717197
 0.9999747614617875
 0.99997351989748
 0.999972477375985
 0.999971581819283
 0.9999708024214033
 0.9999701194906387
 0.9999695149959349
 0.9999689635664119
 0.9999684842858063
 0.9999680379402751
 0.9999676313806477
 0.9999672634030569
```

[180]:
```
mean(fffnormlistl)
```

[180]:
```
0.9999797643847803
```

The mean is very close to 1.

[181]:
```
std(fffnormlistl)
```

[181]:
```
1.805454636656289e-5
```

The standard deviation is much smaller than the mean.

[182]:
```
max(abs.(ffflistl ./ funlistl .- mean(ffflistl ./ funlistl))...)
```

[182]:
```
6.250954305131451e-5
```

The maximum deviation from the mean is in the fifth decimal place.

The following are the values for Table 2 from this environment.

[183]:
```
getindex(fffnormlistl, nff)
```

[183]:
```
5-element Vector{Float64}:
 1.0000422739278316
 0.9999836461644972
 0.99997351989748
 0.9999695149959349
 0.9999672634030569
```

8.4.2. Warm Surface O2

[184]:
```
ffflistl = map(a->fff(a, valst("o2",0)), alistlong);
```

[185]:
```
funlistl = map(a->fun(a, valst("o2",0)), alistlong);
```

[186]:
```
fffnormlistl = ffflistl ./ funlistl
```

[186]:
```
20-element Vector{Float64}:
 1.000041770832729
 1.0000078175118854
 0.9999950109366527
 0.9999880475001817
 0.999983583452981
 0.9999804396637221
 0.9999780858588511
 0.9999762458315071
 0.9999747598506935
 0.9999735303389217
 0.9999724936505499
```

```
 0.9999716041951869
 0.9999708311839155
 0.9999701527270101
 0.9999695497003618
 0.9999690094277129
 0.9999685321434942
 0.999968084577116
 0.9999676831443989
 0.9999673147950437
```

[187]:
```
mean(fffnormlistl)
```

[187]:
```
0.9999797273661457
```

The mean is very close to 1.

[188]:
```
std(fffnormlistl)
```

[188]:
```
1.792108740475664e-5
```

The standard deviation is much smaller than the mean.

[189]:
```
max(abs.(ffflistl ./ funlistl .- mean(ffflistl ./ funlistl))...)
```

[189]:
```
6.204346658322812e-5
```

The maximum deviation from the mean is in the fifth decimal place.

The following are the values for Table 2 from this environment.

[190]:
```
getindex(fffnormlistl, nff)
```

[190]:
```
5-element Vector{Float64}:
 1.000041770832729
 0.999983583452981
 0.9999735303389217
 0.9999695497003618
 0.9999673147950437
```

8.4.3. Cold Surface N2

[191]:
```
ffflistl = map(a->fff(a, valst("n2",0, temp="c")), alistlong);
```

[192]:
```
funlistl = map(a->fun(a, valst("n2",0, temp="c")), alistlong);
```

[193]:
```
fffnormlistl = ffflistl ./ funlistl
```

[193]:
```
20-element Vector{Float64}:
 1.0000328854478497
```

```
 1.0000010797613668
 0.999989020460026
 0.9999824360578193
 0.9999782055831516
 0.9999752183474854
 0.9999729779993168
 0.9999712238476832
 0.999969805260231
 0.9999686311405512
 0.9999676372530464
 0.9999667861101317
 0.9999660447993731
 0.9999654000049347
 0.9999648145116197
 0.999964297950578
 0.9999638299904993
 0.999963406656838
 0.9999630210451285
 0.9999626655940121
```

[194]:
```
mean(fffnormlistl)
```

[194]:
```
0.9999744693910821
```

The mean is very close to 1.

[195]:
```
std(fffnormlistl)
```

[195]:
```
1.692157536781091e-5
```

The standard deviation is much smaller than the mean.

[196]:
```
max(abs.(ffflistl ./ funlistl .- mean(ffflistl ./ funlistl))...)
```

[196]:
```
5.84160567675962e-5
```

The maximum deviation from the mean is in the fifth decimal place.

The following are the values for Table 2 from this environment.

[197]:
```
getindex(fffnormlistl, nff)
```

[197]:
```
5-element Vector{Float64}:
 1.0000328854478497
 0.9999782055831516
 0.9999686311405512
 0.9999648145116197
 0.9999626655940121
```

8.4.4. Cold Surface O2

[198]:
```julia
ffflistl = map(a->fff(a, valst("o2",0, temp="c")), alistlong);
```

[199]:
```julia
funlistl = map(a->fun(a, valst("o2",0, temp="c")), alistlong);
```

[200]:
```julia
fffnormlistl = ffflistl ./ funlistl
```

[200]:
```
20-element Vector{Float64}:
 1.0000328742000621
 1.0000011003757872
 0.9999890521538807
 0.9999824748403581
 0.9999782474954214
 0.9999752623135829
 0.9999730252619691
 0.9999712737999279
 0.9999698571513934
 0.9999686830478665
 0.9999676981396904
 0.9999668405905526
 0.9999660942013784
 0.9999654486730899
 0.9999648697134675
 0.9999643586651362
 0.9999638893878453
 0.9999634636514187
 0.9999630841866848
 0.9999627235562006
```

[201]:
```julia
mean(fffnormlistl)
```

[201]:
```
0.9999745160702858
```

The mean is very close to 1.

[202]:
```julia
std(fffnormlistl)
```

[202]:
```
1.6904659556376253e-5
```

The standard deviation is much smaller than the mean.

[203]:
```julia
max(abs.(ffflistl ./ funlistl .- mean(ffflistl ./ funlistl))...)
```

[203]:
```
5.835812977639243e-5
```

The maximum deviation from the mean is in the fifth decimal place.

The following are the values for Table 2 from this environment.

[204]:
```
getindex(fffnormlistl, nff)
```

[204]:
```
5-element Vector{Float64}:
 1.0000328742000621
 0.9999782474954214
 0.9999686830478665
 0.9999648697134675
 0.9999627235562006
```

8.4.5. Depth 1000 m N2

[205]:
```
ffflistl = map(a->fff(a, valst("n2", 1000)), alistlong);
```

[206]:
```
funlistl = map(a->fun(a, valst("n2", 1000)), alistlong);
```

[207]:
```
fffnormlistl = ffflistl ./ funlistl
```

[207]:
```
20-element Vector{Float64}:
 0.9944439991378958
 0.9944373341669628
 0.9944343848519562
 0.9944326255561504
 0.9944314297473622
 0.9944305446538582
 0.9944298579539759
 0.9944293081510701
 0.9944288413237914
 0.9944284571631895
 0.9944281252322742
 0.9944278319892746
 0.9944275744216104
 0.9944273397122341
 0.9944271355415132
 0.9944269548469361
 0.994426781782807
 0.994426632547899
 0.9944264834313161
 0.9944263560985946
```

[208]:
```
mean(fffnormlistl)
```

[208]:
```
0.9944298999155334
```

The mean is close to 1.

[209]:
```
std(fffnormlistl)
```

[209]:
```
4.402410392078019e-6
```

The standard deviation is much smaller than the mean.

```
[210]: max(abs.(ffflistl ./ funlistl .- mean(ffflistl ./ funlistl))...)
```

```
[210]: 1.4099222362395913e-5
```

The maximum deviation from the mean is in the fifth decimal place.

The following are the values for Table 2 from this environment.

```
[211]: getindex(fffnormlistl, nff)
```

```
[211]: 5-element Vector{Float64}:
 0.9944439991378958
 0.9944314297473622
 0.9944284571631895
 0.9944271355415132
 0.9944263560985946
```

8.4.6. Depth 1000 m O2

```
[212]: ffflistl = map(a->fff(a, valst("o2", 1000)), alistlong);
```

```
[213]: funlistl = map(a->fun(a, valst("o2", 1000)), alistlong);
```

```
[214]: fffnormlistl = ffflistl ./ funlistl
```

```
[214]: 20-element Vector{Float64}:
 0.9942484123630143
 0.9942414466108351
 0.9942383692616659
 0.9942365336002847
 0.9942352838246026
 0.9942343572121722
 0.994233641608876
 0.9942330550172012
 0.9942325878078511
 0.9942321734709387
 0.9942318321628771
 0.994231523681671
 0.9942312541692847
 0.9942310178575845
 0.9942307947783692
 0.9942306055162311
 0.9942304288627555
 0.9942302724337988
 0.9942301250228202
 0.9942299810012369
```

[215]:
```
mean(fffnormlistl)
```

[215]:
```
0.9942336848132035
```

The mean is close to 1.

[216]:
```
std(fffnormlistl)
```

[216]:
```
4.5980238775872056e-6
```

The standard deviation is much smaller than the mean.

[217]:
```
max(abs.(ffflistl ./ funlistl .- mean(ffflistl ./ funlistl))...)
```

[217]:
```
1.4727549810800156e-5
```

The maximum deviation from the mean is in the fifth decimal place.

The following are the values for Table 2 from this environment.

[218]:
```
getindex(fffnormlistl, nff)
```

[218]:
```
5-element Vector{Float64}:
 0.9942484123630143
 0.9942352838246026
 0.9942321734709387
 0.9942307947783692
 0.9942299810012369
```

8.4.7. Depth 2000 m N2

[219]:
```
ffflistl = map(a->fff(a, valst("n2", 2000)), alistlong);
```

[220]:
```
funlistl = map(a->fun(a, valst("n2", 2000)), alistlong);
```

[221]:
```
fffnormlistl = ffflistl ./ funlistl
```

[221]:
```
20-element Vector{Float64}:
 0.9881622747828717
 0.9881546239020846
 0.9881512347172852
 0.9881492162627445
 0.9881478393153572
 0.9881468251685518
 0.988146032386607
 0.9881453931276593
 0.9881448664777331
 0.9881444163486393
 0.9881440365520092
```

```
 0.9881437044817037
 0.9881434079258296
 0.9881431444899327
 0.9881429118522854
 0.9881426944523174
 0.9881424971930852
 0.9881423169181096
 0.9881421595452688
 0.9881419994684596
```

[222]:
```
mean(fffnormlistl)
```

[222]:
```
0.9881460797684267
```

The mean is close to 1.

[223]:
```
std(fffnormlistl)
```

[223]:
```
5.05826296948031e-6
```

The standard deviation is much smaller than the mean.

[224]:
```
max(abs.(ffflistl ./ funlistl .- mean(ffflistl ./ funlistl))...)
```

[224]:
```
1.619501444494187e-5
```

The maximum deviation from the mean is in the fifth decimal place.

The following are the values for Table 2 from this environment.

[225]:
```
getindex(fffnormlistl, nff)
```

[225]:
```
5-element Vector{Float64}:
 0.9881622747828717
 0.9881478393153572
 0.9881444163486393
 0.9881429118522854
 0.9881419994684596
```

8.4.8. Depth 2000 m O2

[226]:
```
ffflistl = map(a->fff(a, valst("o2", 2000)), alistlong);
```

[227]:
```
funlistl = map(a->fun(a, valst("o2", 2000)), alistlong);
```

[228]:
```
fffnormlistl = ffflistl ./ funlistl
```

[228]:
```
20-element Vector{Float64}:
 0.9870448426872233
```

```
 0.9870362365080702
 0.987032429053879
 0.9870301519434979
 0.9870286097613181
 0.9870274732053747
 0.9870265775294275
 0.9870258702209873
 0.9870252746132757
 0.987024771982518
 0.9870243346455848
 0.9870239602484834
 0.9870236261053222
 0.9870233315257054
 0.9870230668360946
 0.9870228263609525
 0.9870226076951586
 0.9870224033901452
 0.9870222237166362
 0.9870220541275683
```

[229]:
```
mean(fffnormlistl)
```

[229]:
```
0.9870266336078611
```

The mean is close to 1.

[230]:
```
std(fffnormlistl)
```

[230]:
```
5.686456907489979e-6
```

The standard deviation is much smaller than the mean.

[231]:
```
max(abs.(ffflistl ./ funlistl .- mean(ffflistl ./ funlistl))...)
```

[231]:
```
1.820907936211924e-5
```

The maximum deviation from the mean is in the fifth decimal place.

The following are the values for Table 2 from this environment.

[232]:
```
getindex(fffnormlistl, nff)
```

[232]:
```
5-element Vector{Float64}:
 0.9870448426872233
 0.9870286097613181
 0.987024771982518
 0.9870230668360946
 0.9870220541275683
```

8.4.9. Deep N2

[233]:
```
ffflistl = map(a->fff(a, valst("n2",3500)), alistlong);
```

[234]:
```
funlistl = map(a->fun(a, valst("n2",3500)), alistlong);
```

[235]:
```
fffnormlistl = ffflistl ./ funlistl
```

[235]:
```
20-element Vector{Float64}:
 0.9794002936819295
 0.9793918473674884
 0.9793881009121015
 0.9793858718979817
 0.9793843570910541
 0.9793832343722773
 0.9793823599533249
 0.9793816531569284
 0.9793810752744049
 0.9793805809301561
 0.9793801567746211
 0.979379783865793
 0.9793794619162964
 0.9793791680393665
 0.9793789115114189
 0.9793786735406113
 0.9793784657681186
 0.979378261394295
 0.9793780873263287
 0.9793779150732873
```

[236]:
```
mean(fffnormlistl)
```

[236]:
```
0.9793824129923892
```

The mean is close to 1.

[237]:
```
std(fffnormlistl)
```

[237]:
```
5.5841293573777175e-6
```

The standard deviation is much smaller than the mean.

[238]:
```
max(abs.(ffflistl ./ funlistl .- mean(ffflistl ./ funlistl))...)
```

[238]:
```
1.788068954033939e-5
```

The maximum deviation from the mean is in the fifth decimal place.

The following are the values for Table 2 from this environment.

[239]:
```
getindex(fffnormlistl, nff)
```

[239]:
```
5-element Vector{Float64}:
 0.9794002936819295
 0.9793843570910541
 0.9793805809301561
 0.9793789115114189
 0.9793779150732873
```

8.4.10. Deep O2

[240]:
```
ffflistl = map(a->fff(a, valst("o2",3500)), alistlong);
```

[241]:
```
funlistl = map(a->fun(a, valst("o2",3500)), alistlong);
```

[242]:
```
fffnormlistl = ffflistl ./ funlistl
```

[242]:
```
20-element Vector{Float64}:
 0.9770490288487784
 0.9770393448735859
 0.9770350540362935
 0.977032503633424
 0.9770307547566641
 0.9770294699055806
 0.9770284693581641
 0.9770276638423463
 0.9770269934080292
 0.9770264303594661
 0.977025938742948
 0.9770255201240959
 0.9770251424641015
 0.9770248126601413
 0.9770245158390738
 0.9770242418854735
 0.9770239956628581
 0.977023770580724
 0.977023562018466
 0.9770233717491819
```

[243]:
```
mean(fffnormlistl)
```

[243]:
```
0.9770285292374699
```

The mean is close to 1.

[244]:
```
std(fffnormlistl)
```

[244]:
```
6.402892608643194e-6
```

The standard deviation is much smaller than the mean.

[245]:
```
max(abs.(ffflistl ./ funlistl .- mean(ffflistl ./ funlistl))...)
```

[245]:
```
2.049961130856648e-5
```

The maximum deviation from the mean is in the fifth decimal place.

The following are the values for Table 2 from this environment.

[246]:
```
getindex(fffnormlistl, nff)
```

[246]:
```
5-element Vector{Float64}:
 0.9770490288487784
 0.9770307547566641
 0.9770264303594661
 0.9770245158390738
 0.9770233717491819
```

8.5. Definitions

Define the acoustic damping factor as the total damping factor minus the viscous and thermal damping factors.

[247]:
```
βac = β - βvis - βth
```

[247]:

$$\frac{a\omega^2}{2c\left(\frac{a^2\omega^2}{c^2}+1\right)}$$

Define a function for the resonant angular frequency for undamped motion using the previously-defined `fun` function.

[248]:
```
"""
    ωun(a1, v1)
Solve for the angular frequency for undamped motion.
"""
ωun(a1, v1) = 2*π * fun(a1, v1)
```

[248]:
```
ωun
```

8.6. Damping factors vs. normalized frequency

Here we explore the variation of the damping factors with normalized frequency.

Recall the symbolic expression for the viscous damping factor.

[249]:
```
βvis
```

[249]:

$$\frac{2\mu}{a^2\rho}$$

Convert the symbolic expression `βvis` to a Julia function.

[250]:
```
βvisjl = lambdify(βvis,(a, vlist...))
```

[250]: #118 (generic function with 1 method)

Recall the symbolic expression for the acoustic damping factor.

[251]:
```
βac
```

[251]:

$$\frac{a\omega^2}{2c\left(\frac{a^2\omega^2}{c^2}+1\right)}$$

Convert the symbolic expression `βac` to a Julia function.

[252]:
```
βacjl = lambdify(βac(ω=>2*π*f),(a, vlist..., f))
```

[252]: #118 (generic function with 1 method)

Recall the symbolic expression for the thermal damping factor.

Symbolic output from the expression below has been suppressed. To show the output, delete the trailing semicolon before entering the entering the expression.

[253]:
```
βth;
```

Convert the symbolic expression `βth` to a Julia function.

[254]:
```
βthjl = lambdify(βth(ω=>2*π*f), (a, vlist..., f))
```

[254]: #118 (generic function with 1 method)

Recall the symbolic expression for the (total) damping factor.

Symbolic output from the expression below has been suppressed. To show the output, delete the trailing semicolon before entering the entering the expression.

[255]:
```
β;
```

Convert the symbolic expression `β` to a Julia function.

[256]:
```
βjl = lambdify(β(ω=>2*π*f), (a, vlist..., f))
```

[256]: #118 (generic function with 1 method)

Now crate arrays of each damping factor value at different frequencies to plot.

8.6.1. N2 Surface

The parameter β_{vis} is not frequency dependent. Create an array of constant values to plot.

[257]:
```
bvisns = βvisjl(0.1, valst("n2", 0)...) /
    ωun(0.1, valst("n2", 0)) .* ones(length(fnorm));
```

[258]:
```
bacns = map(f1 -> βacjl(0.1, valst("n2", 0)..., f1),
    fun(0.1, valst("n2", 0)) .* fnorm) ./ ωun(0.1, valst("n2", 0));
```

[259]:
```
bthns = map(f1->βthjl(0.1, valst("n2", 0)..., f1),
    fun(0.1, valst("n2", 0)) .* fnorm) ./ ωun(0.1, valst("n2", 0));
```

[260]:
```
bns = map(f1 -> βjl(0.1, valst("n2", 0)..., f1),
    fun(0.1, valst("n2", 0)) .* fnorm) ./ ωun(0.1, valst("n2", 0));
```

[261]:
```
loglog(fnorm, bvisns, linestyle="-", color=get_cmap("plasma")(0.25), label="Viscous")
loglog(fnorm, bacns, linestyle="-", color=get_cmap("plasma")(0.5), label="Acoustic")
loglog(fnorm, bthns, linestyle="-", color=get_cmap("plasma")(0.75), label="Thermal")
loglog(fnorm, bns, ":k", label="Total")
xlabel("Normalized Frequency")
ylabel(L"$\beta'$")
legend();
```

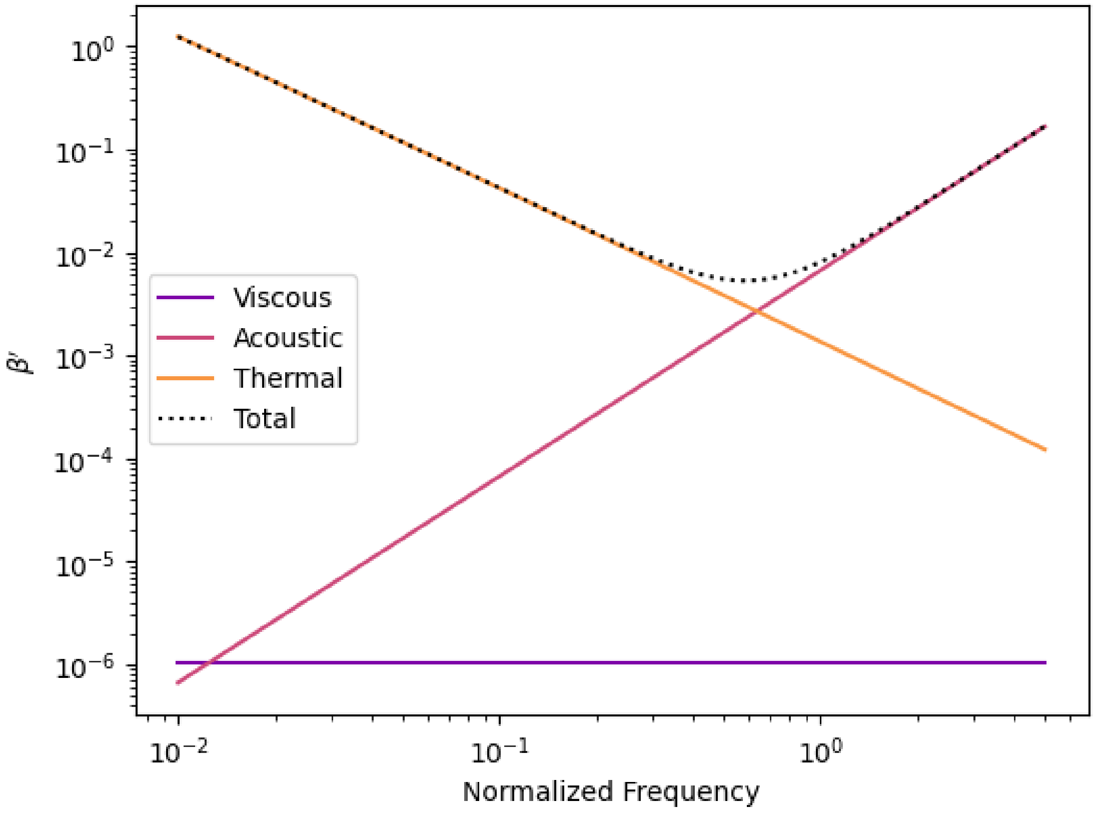

This is Fig. 4A.

8.6.2. O2 Surface

```
[262]: bvisos = βvisjl(0.1, valst("o2", 0)...) /
           ωun(0.1, valst("o2", 0)) .* ones(length(fnorm));
```

```
[263]: bacos = map(f1 -> βacjl(0.1, valst("o2", 0)..., f1),
           fun(0.1, valst("o2", 0)) .* fnorm) ./ ωun(0.1, valst("o2", 0));
```

```
[264]: bthos = map(f1->βthjl(0.1, valst("o2", 0)..., f1),
           fun(0.1, valst("o2", 0)) .* fnorm) ./ ωun(0.1, valst("o2", 0));
```

```
[265]: bos = map(f1 -> βjl(0.1, valst("o2", 0)..., f1),
           fun(0.1, valst("o2", 0)) .* fnorm) ./ ωun(0.1, valst("o2", 0));
```

```
[266]: loglog(fnorm, bvisos, linestyle="-", color=get_cmap("plasma")(0.25), label="Viscous")
       loglog(fnorm, bacos, linestyle="-", color=get_cmap("plasma")(0.5), label="Acoustic")
       loglog(fnorm, bthos, linestyle="-", color=get_cmap("plasma")(0.75), label="Thermal")
       loglog(fnorm, bos, ":k", label="Total")
       xlabel("Normalized Frequency")
       ylabel(L"$\beta'$")
       legend();
```

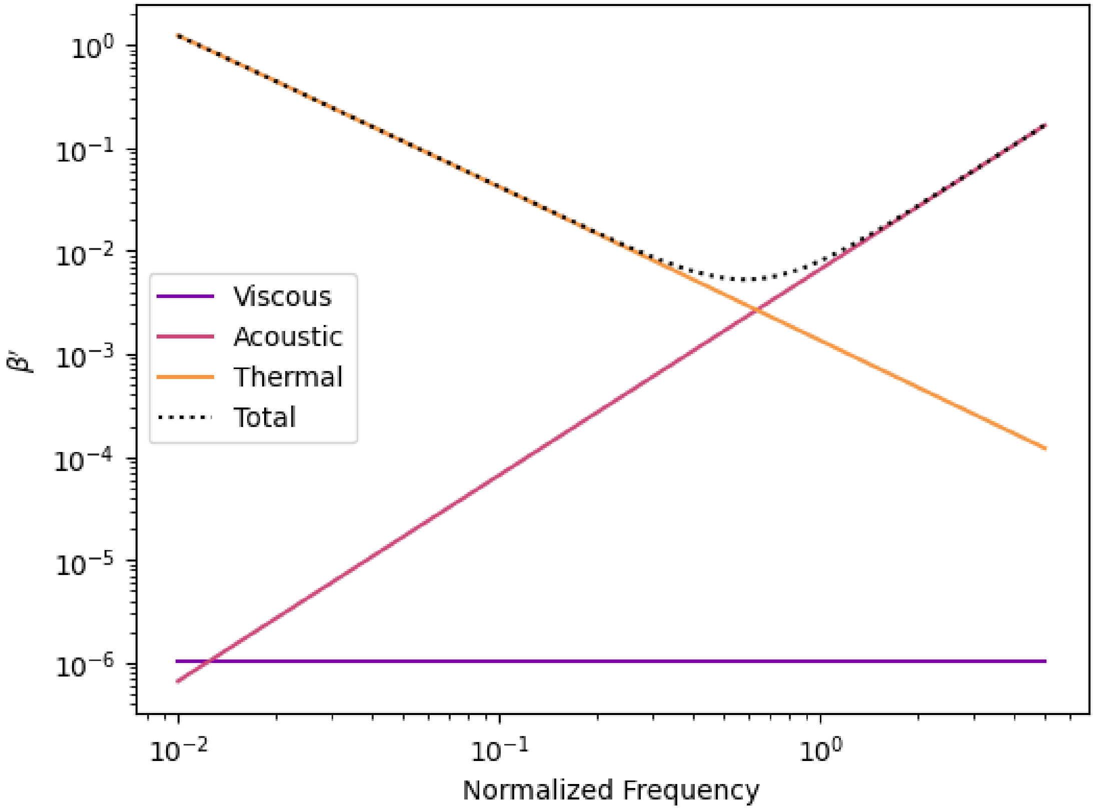

This is Fig. 4B.

8.6.3. N2 Cold Surface

[267]:
```
bvisncs = βvisjl(0.1, valst("n2", 0, temp="c")...) /
    wun(0.1, valst("n2", 0, temp="c")) .* ones(length(fnorm));
```

[268]:
```
bacncs = map(f1 -> βacjl(0.1, valst("n2", 0, temp="c")..., f1),
    fun(0.1, valst("n2", 0, temp="c")) .* fnorm) ./ wun(0.1, valst("n2", 0, temp="c"));
```

[269]:
```
bthncs = map(f1->βthjl(0.1, valst("n2", 0, temp="c")..., f1),
    fun(0.1, valst("n2", 0, temp="c")) .* fnorm) ./ wun(0.1, valst("n2", 0, temp="c"));
```

[270]:
```
bncs = map(f1 -> βjl(0.1, valst("n2", 0, temp="c")..., f1),
    fun(0.1, valst("n2", 0, temp="c")) .* fnorm) ./ wun(0.1, valst("n2", 0, temp="c"));
```

[271]:
```
loglog(fnorm, bvisncs, linestyle="-", color=get_cmap("plasma")(0.25), label="Viscous")
loglog(fnorm, bacncs, linestyle="-", color=get_cmap("plasma")(0.5), label="Acoustic")
loglog(fnorm, bthncs, linestyle="-", color=get_cmap("plasma")(0.75), label="Thermal")
loglog(fnorm, bncs, ":k", label="Total")
xlabel("Normalized Frequency")
ylabel(L"$\beta'$")
legend();
```

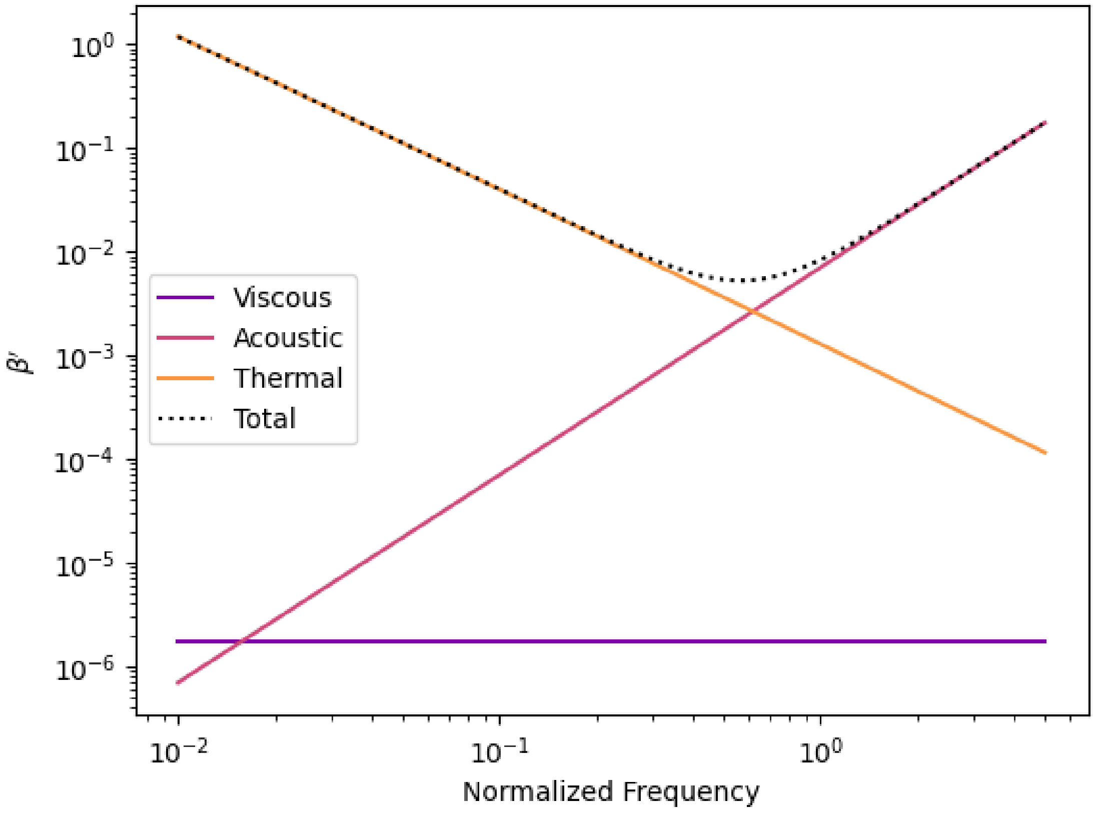

This is Fig. 4C.

8.6.4. O2 Cold Surface

[272]:
```
bvisocs = βvisjl(0.1, valst("o2", 0, temp="c")...) /
    ωun(0.1, valst("o2", 0, temp="c")) .* ones(length(fnorm));
```

[273]:
```
bacocs = map(f1 -> βacjl(0.1, valst("o2", 0, temp="c")..., f1),
    fun(0.1, valst("o2", 0, temp="c")) .* fnorm) ./ ωun(0.1, valst("o2", 0, temp="c"));
```

[274]:
```
bthocs = map(f1->βthjl(0.1, valst("o2", 0, temp="c")..., f1),
    fun(0.1, valst("o2", 0, temp="c")) .* fnorm) ./ ωun(0.1, valst("o2", 0, temp="c"));
```

[275]:
```
bocs = map(f1 -> βjl(0.1, valst("o2", 0, temp="c")..., f1),
    fun(0.1, valst("o2", 0, temp="c")) .* fnorm) ./ ωun(0.1, valst("o2", 0, temp="c"));
```

[276]:
```
loglog(fnorm, bvisocs, linestyle="-", color=get_cmap("plasma")(0.25), label="Viscous")
loglog(fnorm, bacocs, linestyle="-", color=get_cmap("plasma")(0.5), label="Acoustic")
loglog(fnorm, bthocs, linestyle="-", color=get_cmap("plasma")(0.75), label="Thermal")
loglog(fnorm, bocs, ":k", label="Total")
xlabel("Normalized Frequency")
ylabel(L"$\beta'$")
legend();
```

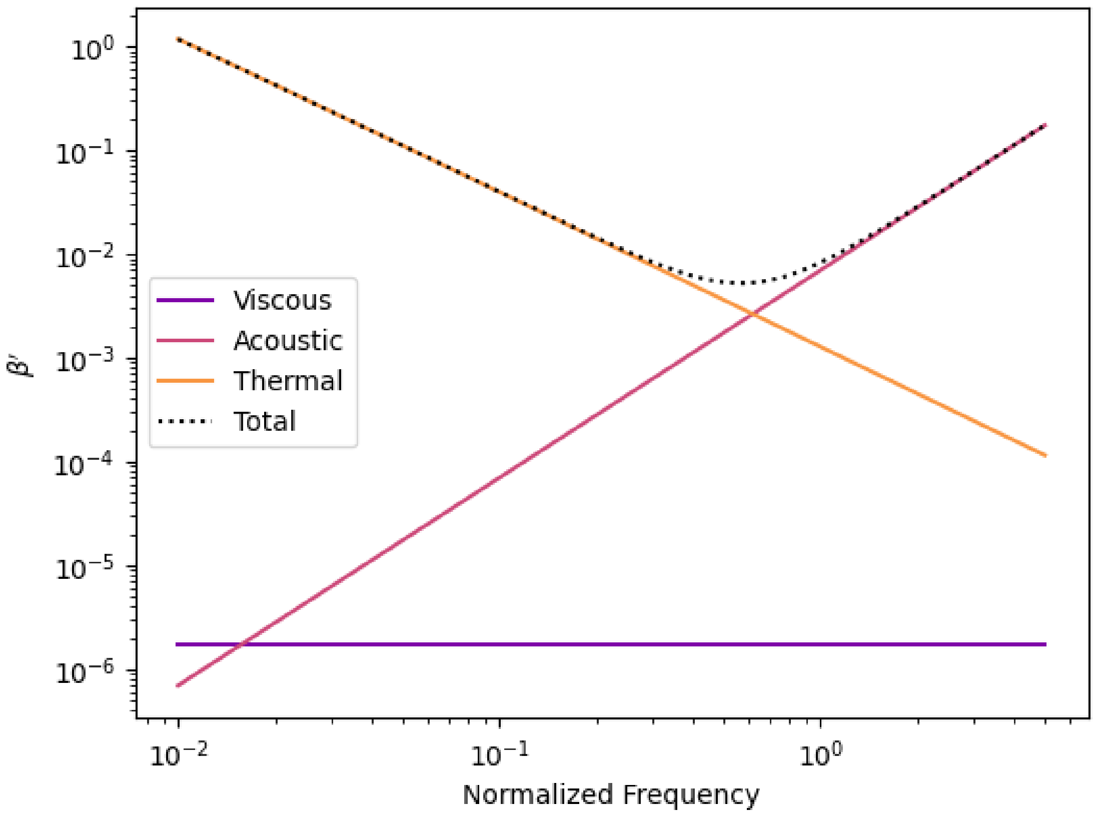

This is Fig. 4D.

8.6.5. N2 Depth 1000 m

[277]:
```
bvisn1k = βvisjl(0.1, valst("n2", 1000)...) /
    ωun(0.1, valst("n2", 1000)) .* ones(length(fnorm));
```

[278]:
```
bacn1k = map(f1 -> βacjl(0.1, valst("n2", 1000)..., f1),
    fun(0.1, valst("n2", 1000)) .* fnorm) ./ ωun(0.1, valst("n2", 1000));
```

[279]:
```
bthn1k = map(f1->βthjl(0.1, valst("n2", 1000)..., f1),
    fun(0.1, valst("n2", 1000)) .* fnorm) ./ ωun(0.1, valst("n2", 1000));
```

[280]:
```
bn1k = map(f1 -> βjl(0.1, valst("n2", 1000)..., f1),
    fun(0.1, valst("n2", 1000)) .* fnorm) ./ ωun(0.1, valst("n2", 1000));
```

[281]:
```
loglog(fnorm, bvisn1k, linestyle="-", color=get_cmap("plasma")(0.25), label="Viscous")
loglog(fnorm, bacn1k, linestyle="-", color=get_cmap("plasma")(0.5), label="Acoustic")
loglog(fnorm, bthn1k, linestyle="-", color=get_cmap("plasma")(0.75), label="Thermal")
loglog(fnorm, bn1k, ":k", label="Total")
xlabel("Normalized Frequency")
```

```
ylabel(L"$\beta'$")
legend();
```

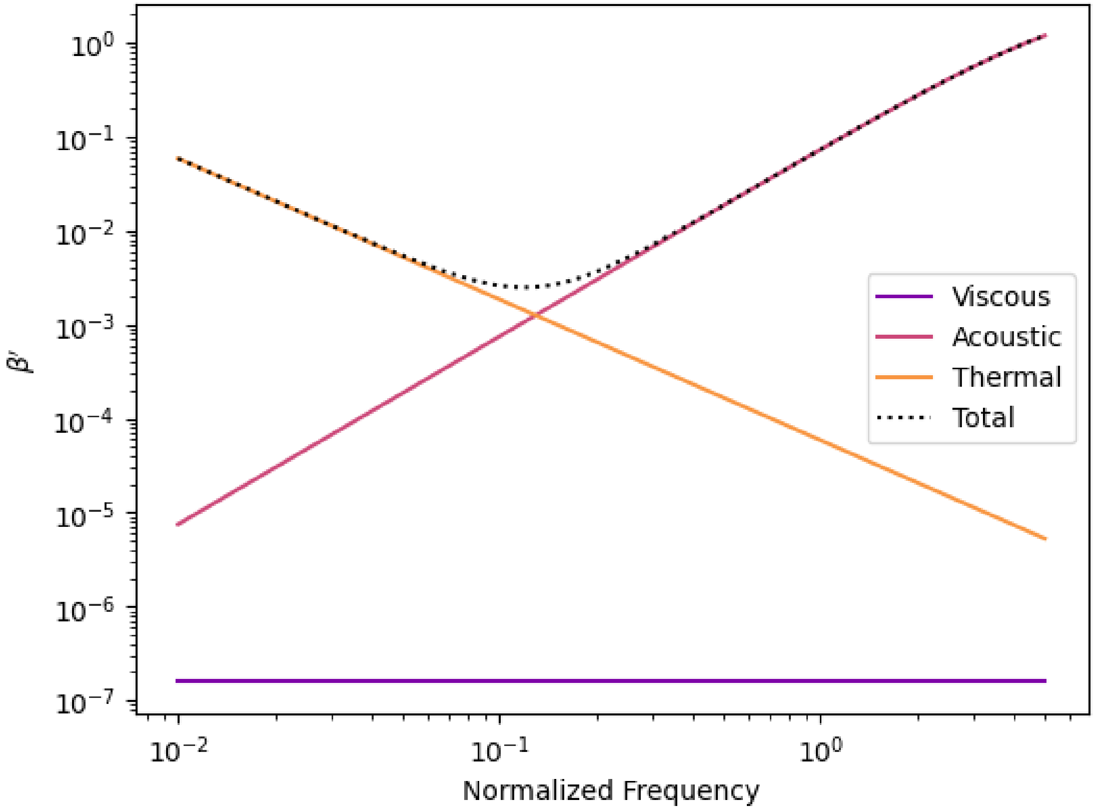

This is Fig. 4E.

8.6.6. O2 Depth 1000 m

[282]:
```
bviso1k = βvisjl(0.1, valst("o2", 1000)...) /
    ωun(0.1, valst("o2", 1000)) .* ones(length(fnorm));
```

[283]:
```
baco1k = map(f1 -> βacjl(0.1, valst("o2", 1000)..., f1),
    fun(0.1, valst("o2", 1000)) .* fnorm) ./ ωun(0.1, valst("o2", 1000));
```

[284]:
```
btho1k = map(f1->βthjl(0.1, valst("o2", 1000)..., f1),
    fun(0.1, valst("o2", 1000)) .* fnorm) ./ ωun(0.1, valst("o2", 1000));
```

[285]:
```
bo1k = map(f1 -> βjl(0.1, valst("o2", 1000)..., f1),
    fun(0.1, valst("o2", 1000)) .* fnorm) ./ ωun(0.1, valst("o2", 1000));
```

[286]:
```
loglog(fnorm, bviso1k, linestyle="-", color=get_cmap("plasma")(0.25), label="Viscous")
loglog(fnorm, baco1k, linestyle="-", color=get_cmap("plasma")(0.5), label="Acoustic")
```

```
loglog(fnorm, btho1k, linestyle="-", color=get_cmap("plasma")(0.75), label="Thermal")
loglog(fnorm, bo1k, ":k", label="Total")
xlabel("Normalized Frequency")
ylabel(L"$\beta'$")
legend();
```

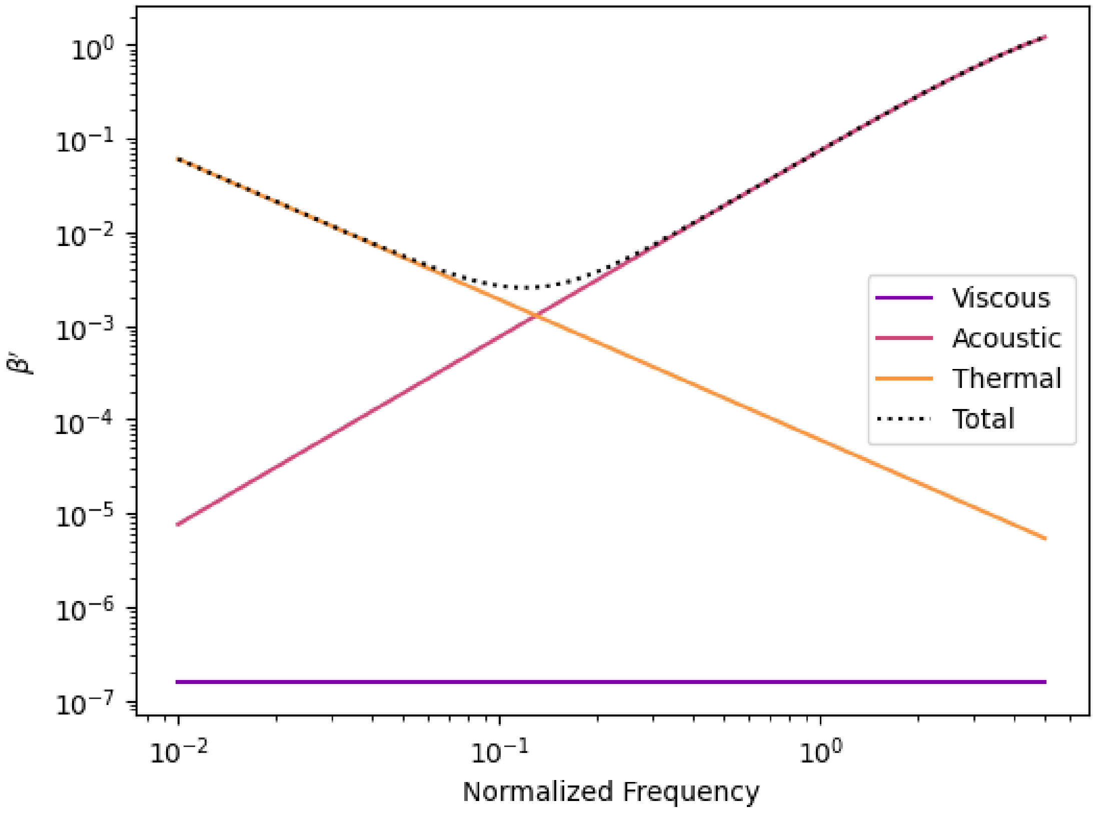

This is Fig. 4F.

8.6.7. N2 Depth 2000 m

[287]:
```
bvisn2k = βvisjl(0.1, valst("n2", 2000)...) /
    ωun(0.1, valst("n2", 2000)) .* ones(length(fnorm));
```

[288]:
```
bacn2k = map(f1 -> βacjl(0.1, valst("n2", 2000)..., f1),
    fun(0.1, valst("n2", 2000)) .* fnorm) ./ ωun(0.1, valst("n2", 2000));
```

[289]:
```
bthn2k = map(f1->βthjl(0.1, valst("n2", 2000)..., f1),
    fun(0.1, valst("n2", 2000)) .* fnorm) ./ ωun(0.1, valst("n2", 2000));
```

[290]:
```
bn2k = map(f1 -> βjl(0.1, valst("n2", 2000)..., f1),
    fun(0.1, valst("n2", 2000)) .* fnorm) ./ ωun(0.1, valst("n2", 2000));
```

[291]:
```
loglog(fnorm, bvisn2k, linestyle="-", color=get_cmap("plasma")(0.25), label="Viscous")
loglog(fnorm, bacn2k, linestyle="-", color=get_cmap("plasma")(0.5), label="Acoustic")
loglog(fnorm, bthn2k, linestyle="-", color=get_cmap("plasma")(0.75), label="Thermal")
loglog(fnorm, bn2k, ":k", label="Total")
xlabel("Normalized Frequency")
ylabel(L"$\beta'$")
legend();
```

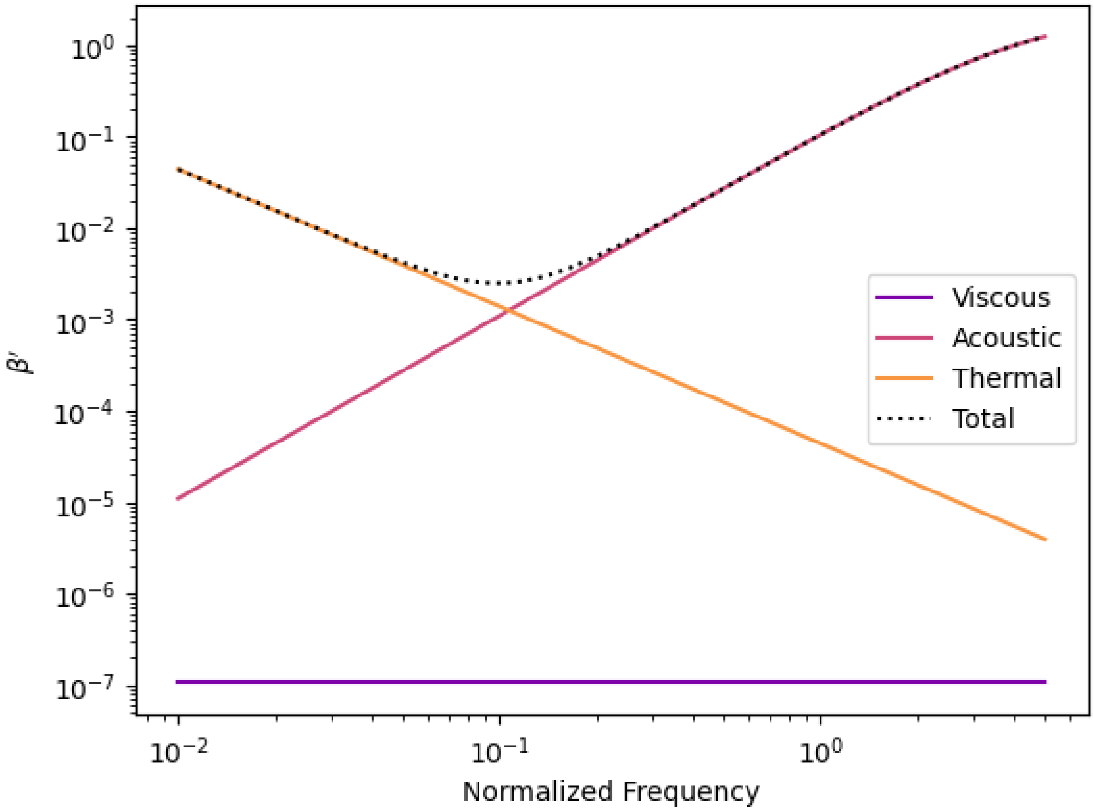

This is Fig. 4E.

8.6.8. O2 Depth 2000 m

[292]:
```
bviso2k = βvisjl(0.1, valst("o2", 2000)...) /
    ωun(0.1, valst("o2", 2000)) .* ones(length(fnorm));
```

[293]:
```
baco2k = map(f1 -> βacjl(0.1, valst("o2", 2000)..., f1),
    fun(0.1, valst("o2", 2000)) .* fnorm) ./ ωun(0.1, valst("o2", 2000));
```

[294]:
```
btho2k = map(f1->βthjl(0.1, valst("o2", 2000)..., f1),
    fun(0.1, valst("o2", 2000)) .* fnorm) ./ ωun(0.1, valst("o2", 2000));
```

```
[295]: bo2k = map(f1 -> βjl(0.1, valst("o2", 2000)..., f1),
           fun(0.1, valst("o2", 2000)) .* fnorm) ./ ωun(0.1, valst("o2", 2000));
```

```
[296]: loglog(fnorm, bviso2k, linestyle="-", color=get_cmap("plasma")(0.25), label="Viscous")
       loglog(fnorm, baco2k, linestyle="-", color=get_cmap("plasma")(0.5), label="Acoustic")
       loglog(fnorm, btho2k, linestyle="-", color=get_cmap("plasma")(0.75), label="Thermal")
       loglog(fnorm, bo2k, ":k", label="Total")
       xlabel("Normalized Frequency")
       ylabel(L"$\beta'$")
       legend();
```

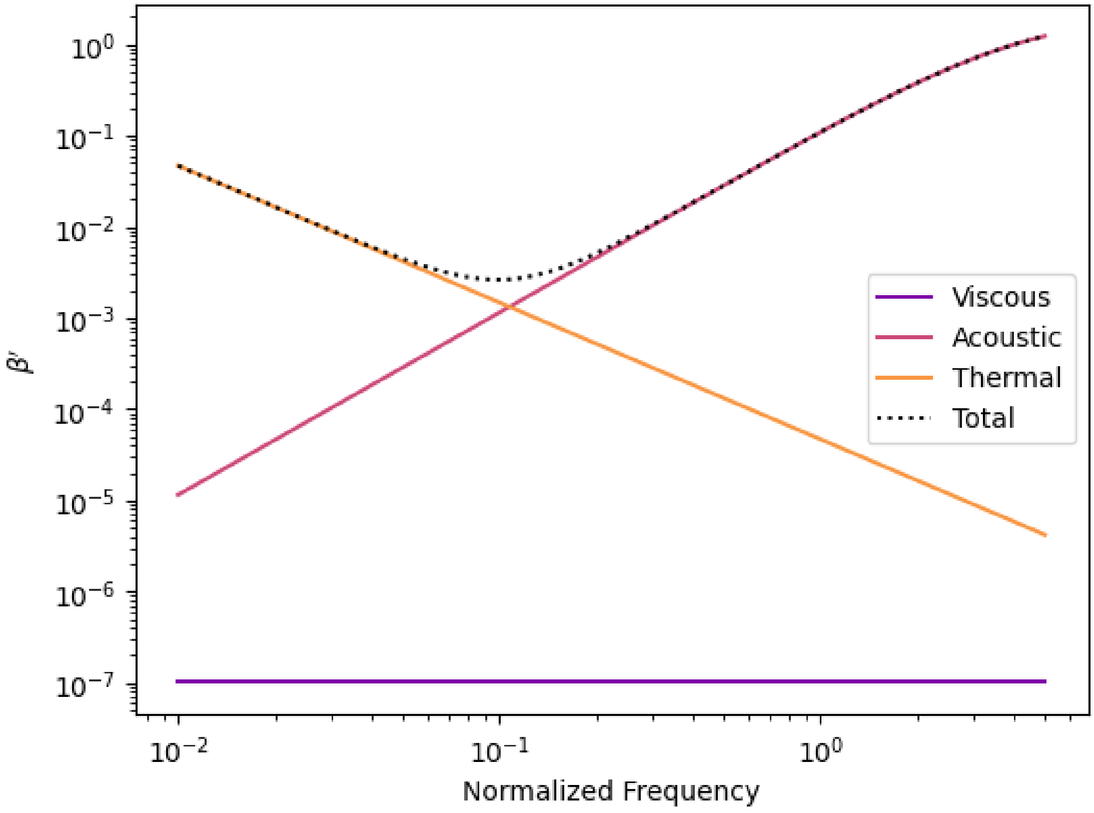

This is Fig. 4F.

8.6.9. N2 Deep

```
[297]: bvisnd = βvisjl(0.1, valst("n2", 3500)...) /
           ωun(0.1, valst("n2", 3500)) .* ones(length(fnorm));
```

```
[298]: bacnd = map(f1 -> βacjl(0.1, valst("n2", 3500)..., f1),
           fun(0.1, valst("n2", 3500)) .* fnorm) ./ ωun(0.1, valst("n2", 3500));
```

```
[299]: bthnd = map(f1->βthjl(0.1, valst("n2", 3500)..., f1),
           fun(0.1, valst("n2", 3500)) .* fnorm) ./ ωun(0.1, valst("n2", 3500));
```

```
[300]: bnd = map(f1 -> βjl(0.1, valst("n2", 3500)..., f1),
           fun(0.1, valst("n2", 3500)) .* fnorm) ./ ωun(0.1, valst("n2", 3500));
```

```
[301]: loglog(fnorm, bvisnd, linestyle="-", color=get_cmap("plasma")(0.25), label="Viscous")
       loglog(fnorm, bacnd, linestyle="-", color=get_cmap("plasma")(0.5), label="Acoustic")
       loglog(fnorm, bthnd, linestyle="-", color=get_cmap("plasma")(0.75), label="Thermal")
       loglog(fnorm, bnd, ":k", label="Total")
       xlabel("Normalized Frequency")
       ylabel(L"$\beta'$")
       legend();
```

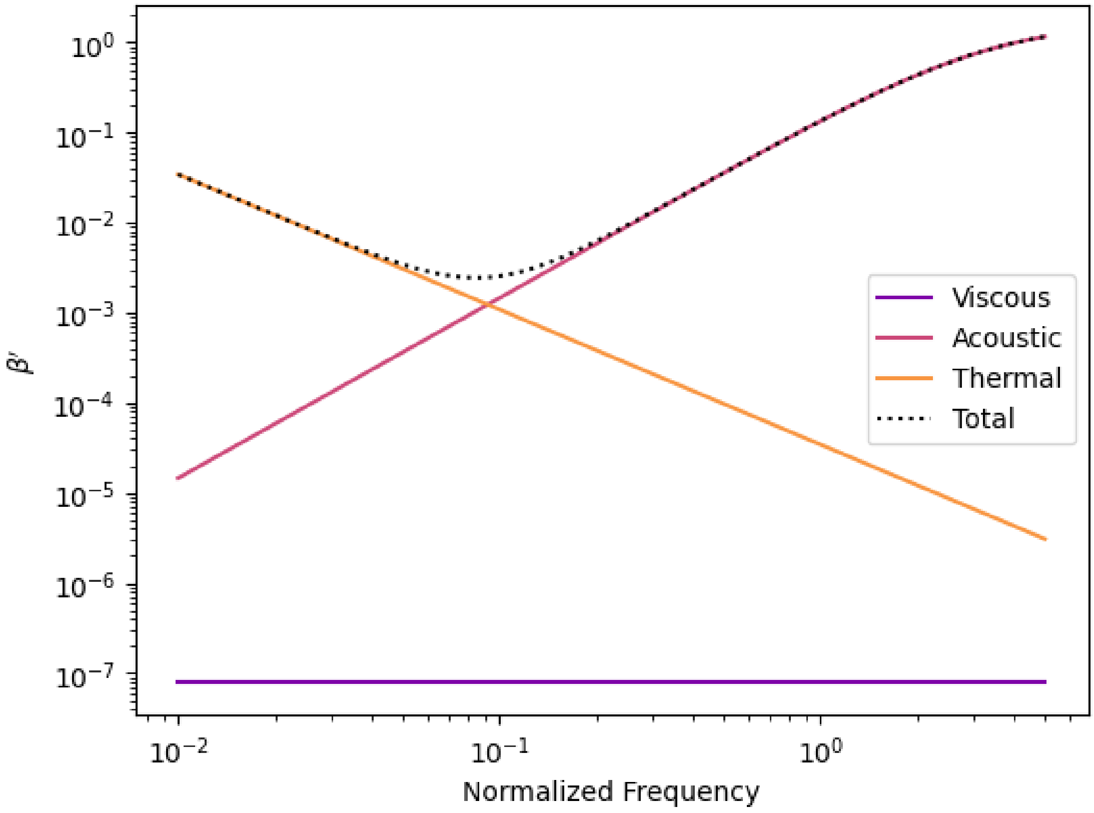

This is Fig. 4E.

8.6.10. O2 Deep

```
[302]: bvisod = βvisjl(0.1, valst("o2", 3500)...) /
           ωun(0.1, valst("o2", 3500)) .* ones(length(fnorm));
```

[303]:
```
bacod = map(f1 -> βacjl(0.1, valst("o2", 3500)..., f1),
    fun(0.1, valst("o2", 3500)) .* fnorm) ./ ωun(0.1, valst("o2", 3500));
```

[304]:
```
bthod = map(f1->βthjl(0.1, valst("o2", 3500)..., f1),
    fun(0.1, valst("o2", 3500)) .* fnorm) ./ ωun(0.1, valst("o2", 3500));
```

[305]:
```
bod = map(f1 -> βjl(0.1, valst("o2", 3500)..., f1),
    fun(0.1, valst("o2", 3500)) .* fnorm) ./ ωun(0.1, valst("o2", 3500));
```

[306]:
```
loglog(fnorm, bvisod, linestyle="-", color=get_cmap("plasma")(0.25), label="Viscous")
loglog(fnorm, bacod, linestyle="-", color=get_cmap("plasma")(0.5), label="Acoustic")
loglog(fnorm, bthod, linestyle="-", color=get_cmap("plasma")(0.75), label="Thermal")
loglog(fnorm, bod, ":k", label="Total")
xlabel("Normalized Frequency")
ylabel(L"$\beta'$")
legend();
```

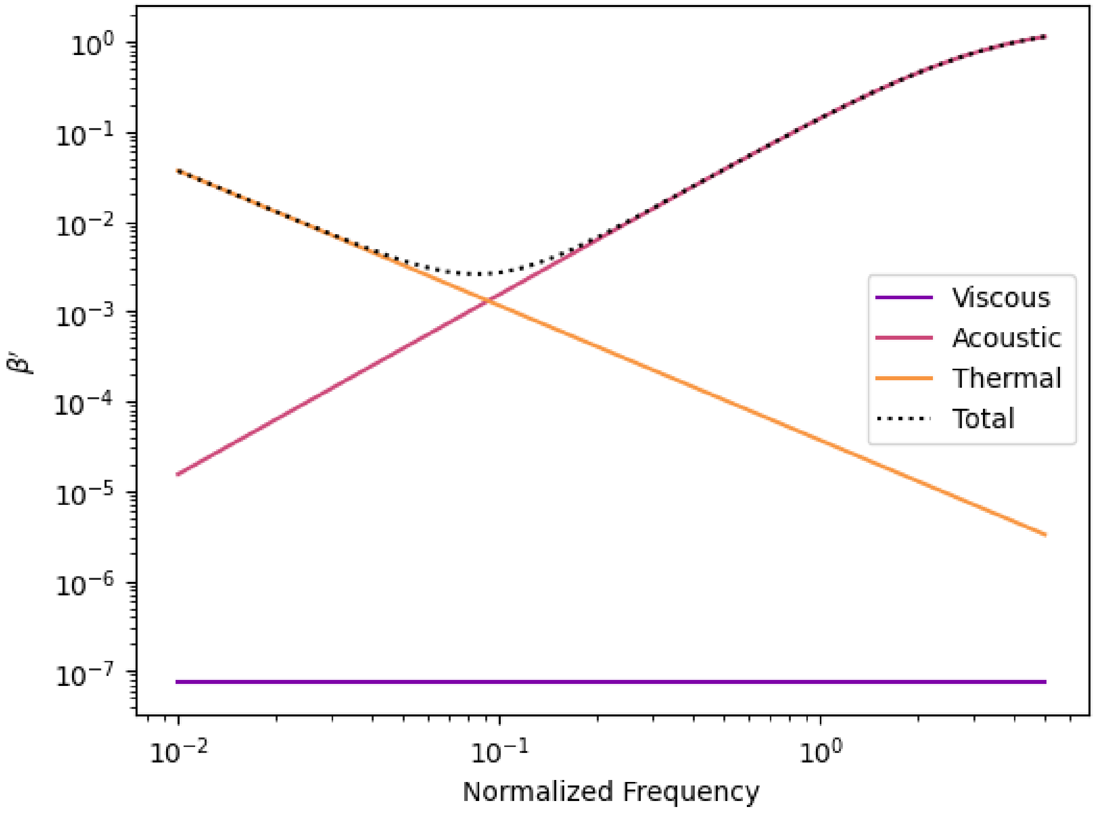

This is Fig. 4F.

8.7. Figure 5

[307]:
```
heights = [2, 2, 2, 2, 2, 1.5]
yt = [1e-6, 1e-3, 1]

fig = figure(constrained_layout=true, dpi=300, figsize=(7.38,5.21))
gs = fig.add_gridspec(6, 4, height_ratios=heights)
ax1 = fig.add_subplot(get(gs, (0, slice(0, 2))))
ax1.loglog(fnorm, bvisns, linestyle="-", color=get_cmap("plasma")(0.25), label="Viscous")
ax1.loglog(fnorm, bacns, linestyle="-", color=get_cmap("plasma")(0.5), label="Acoustic")
ax1.loglog(fnorm, bthns, linestyle="-", color=get_cmap("plasma")(0.75), label="Thermal")
ax1.loglog(fnorm, bns, ":k", label="Total")
ax1.set_yticks(yt)
ax1.set_ylabel(L"$\beta'$")
ax1.set_xticklabels(())

ax2 = fig.add_subplot(get(gs, (0, slice(2, 4))))
ax2.loglog(fnorm, bvisos, linestyle="-", color=get_cmap("plasma")(0.25))
ax2.loglog(fnorm, bacos, linestyle="-", color=get_cmap("plasma")(0.5))
ax2.loglog(fnorm, bthos, linestyle="-", color=get_cmap("plasma")(0.75))
ax2.loglog(fnorm, bos, ":k")
ax2.set_yticks(yt)
ax2.set_xticklabels(())
ax2.set_yticklabels(())

ax3 = fig.add_subplot(get(gs, (1, slice(0, 2))))
ax3.loglog(fnorm, bvisncs, linestyle="-", color=get_cmap("plasma")(0.25))
ax3.loglog(fnorm, bacncs, linestyle="-", color=get_cmap("plasma")(0.5))
ax3.loglog(fnorm, bthncs, linestyle="-", color=get_cmap("plasma")(0.75))
ax3.loglog(fnorm, bncs, ":k")
ax3.set_yticks(yt)
ax3.set_ylabel(L"$\beta'$")
ax3.set_xticklabels(())

ax4 = fig.add_subplot(get(gs, (1, slice(2, 4))))
ax4.loglog(fnorm, bvisocs, linestyle="-", color=get_cmap("plasma")(0.25))
ax4.loglog(fnorm, bacocs, linestyle="-", color=get_cmap("plasma")(0.5))
ax4.loglog(fnorm, bthocs, linestyle="-", color=get_cmap("plasma")(0.75))
ax4.loglog(fnorm, bocs, ":k")
ax4.set_yticks(yt)
ax4.set_xticklabels(())
ax4.set_yticklabels(())

ax5 = fig.add_subplot(get(gs, (2, slice(0, 2))))
ax5.loglog(fnorm, bvisn1k, linestyle="-", color=get_cmap("plasma")(0.25))
ax5.loglog(fnorm, bacn1k, linestyle="-", color=get_cmap("plasma")(0.5))
ax5.loglog(fnorm, bthn1k, linestyle="-", color=get_cmap("plasma")(0.75))
```

```
ax5.loglog(fnorm, bn1k, ":k")
ax5.set_yticks(yt)
ax5.set_ylabel(L"$\beta'$")
ax5.set_xticklabels(())

ax6 = fig.add_subplot(get(gs, (2, slice(2, 4))))
ax6.loglog(fnorm, bviso1k, linestyle="-", color=get_cmap("plasma")(0.25))
ax6.loglog(fnorm, baco1k, linestyle="-", color=get_cmap("plasma")(0.5))
ax6.loglog(fnorm, btho1k, linestyle="-", color=get_cmap("plasma")(0.75))
ax6.loglog(fnorm, bo1k, ":k")
ax6.set_yticks(yt)
ax6.set_xticklabels(())
ax6.set_yticklabels(())

ax7 = fig.add_subplot(get(gs, (3, slice(0, 2))))
ax7.loglog(fnorm, bvisn2k, linestyle="-", color=get_cmap("plasma")(0.25))
ax7.loglog(fnorm, bacn2k, linestyle="-", color=get_cmap("plasma")(0.5))
ax7.loglog(fnorm, bthn2k, linestyle="-", color=get_cmap("plasma")(0.75))
ax7.loglog(fnorm, bn2k, ":k")
ax7.set_yticks(yt)
ax7.set_ylabel(L"$\beta'$")
ax7.set_xticklabels(())

ax8 = fig.add_subplot(get(gs, (3, slice(2, 4))))
ax8.loglog(fnorm, bviso2k, linestyle="-", color=get_cmap("plasma")(0.25))
ax8.loglog(fnorm, baco2k, linestyle="-", color=get_cmap("plasma")(0.5))
ax8.loglog(fnorm, btho2k, linestyle="-", color=get_cmap("plasma")(0.75))
ax8.loglog(fnorm, bo2k, ":k")
ax8.set_yticks(yt)
ax8.set_xticklabels(())
ax8.set_yticklabels(())

ax9 = fig.add_subplot(get(gs, (4, slice(0, 2))))
ax9.loglog(fnorm, bvisnd, linestyle="-", color=get_cmap("plasma")(0.25))
ax9.loglog(fnorm, bacnd, linestyle="-", color=get_cmap("plasma")(0.5))
ax9.loglog(fnorm, bthnd, linestyle="-", color=get_cmap("plasma")(0.75))
ax9.loglog(fnorm, bnd, ":k")
ax9.set_yticks(yt)
ax9.set_xlabel("Normalized Frequency")
ax9.set_ylabel(L"$\beta'$")

ax10 = fig.add_subplot(get(gs, (4, slice(2, 4))))
ax10.loglog(fnorm, bvisod, linestyle="-", color=get_cmap("plasma")(0.25))
ax10.loglog(fnorm, bacod, linestyle="-", color=get_cmap("plasma")(0.5))
ax10.loglog(fnorm, bthod, linestyle="-", color=get_cmap("plasma")(0.75))
ax10.loglog(fnorm, bod, ":k")
ax10.set_yticks(yt)
```

```
ax10.set_xlabel("Normalized Frequency")
ax10.set_yticklabels(())

ax1.get_shared_x_axes().join(ax1, ax3, ax5, ax7, ax9)
ax2.get_shared_x_axes().join(ax2, ax4, ax6, ax8, ax10)
ax1.get_shared_y_axes().join(ax1, ax2)
ax3.get_shared_y_axes().join(ax3, ax4)
ax5.get_shared_y_axes().join(ax5, ax6)
ax7.get_shared_y_axes().join(ax7, ax8)
ax9.get_shared_y_axes().join(ax9, ax10)

fig.text(0.092, 0.965, "A")
fig.text(0.554, 0.965, "B")
fig.text(0.092, 0.8, "C")
fig.text(0.554, 0.8, "D")
fig.text(0.092, 0.635, "E")
fig.text(0.554, 0.635, "F")
fig.text(0.092, 0.47, "G")
fig.text(0.554, 0.47, "H")
fig.text(0.092, 0.305, "I")
fig.text(0.554, 0.305, "J")
fig.legend(title="Damping Type", ncol=4, loc="lower center");
```

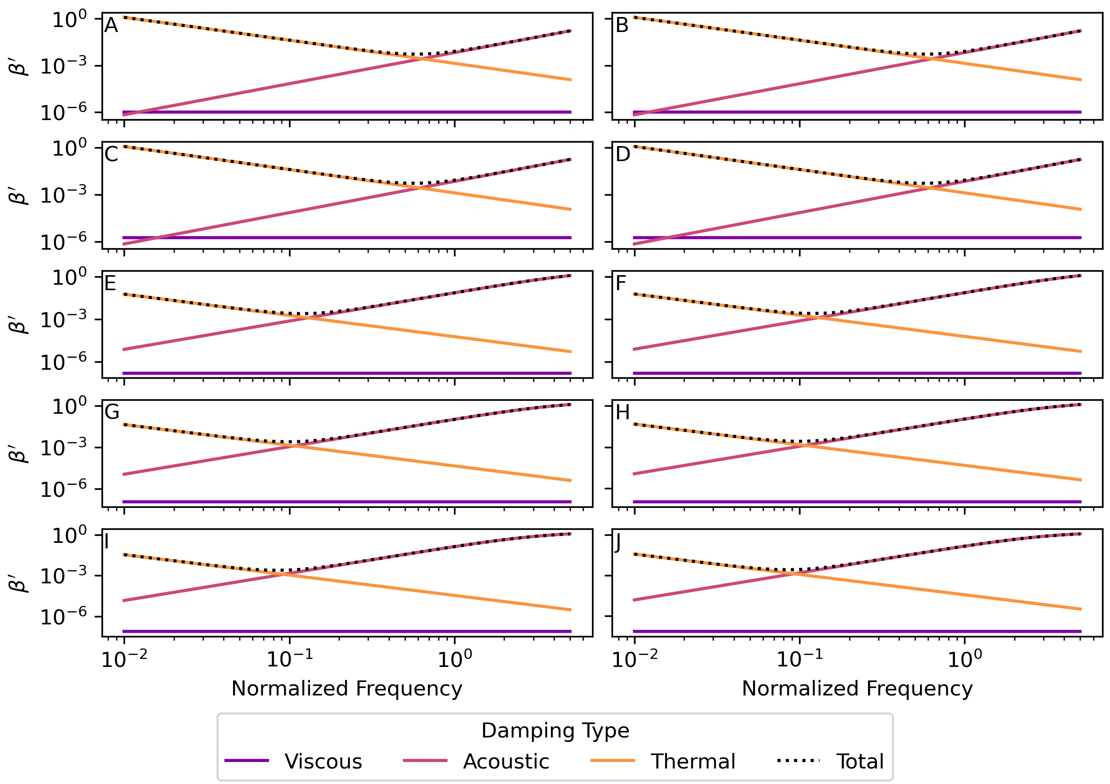

[308]:
```
fig.savefig("Figure5.tiff", bbox_inches="tight")
fig.savefig("Figure5.png", bbox_inches="tight")
```

8.8. Normalized Damping Factors vs. Normalized Frequency

Now we look at the normalized damping factor vs. normalized frequency. We calculate arrays of values for plotting.

8.8.1. N2 Surface

[309]:
```
bnormns1 = map(f1 -> βjl(0.1, valst("n2", 0)..., f1),
    fun(0.1, valst("n2", 0)) .* fnorm) ./ ωun(0.1, valst("n2", 0));
```

[310]:
```
bnormns05 = map(f1 -> βjl(0.05, valst("n2", 0)..., f1),
    fun(0.05, valst("n2", 0)) .* fnorm) ./ ωun(0.05, valst("n2", 0));
```

[311]:
```
bnormns01 = map(f1 -> βjl(0.01, valst("n2", 0)..., f1),
    fun(0.01, valst("n2", 0)) .* fnorm) ./ ωun(0.01, valst("n2", 0));
```

8.8.2. O2 Surface

[312]:
```
bnormos1 = map(f1 -> βjl(0.1, valst("o2", 0)..., f1),
    fun(0.1, valst("o2", 0)) .* fnorm) ./ ωun(0.1, valst("o2", 0));
```

[313]:
```
bnormos05 = map(f1 -> βjl(0.05, valst("o2", 0)..., f1),
    fun(0.05, valst("o2", 0)) .* fnorm) ./ ωun(0.05, valst("o2", 0));
```

[314]:
```
bnormos01 = map(f1 -> βjl(0.01, valst("o2", 0)..., f1),
    fun(0.01, valst("o2", 0)) .* fnorm) ./ ωun(0.01, valst("o2", 0));
```

8.8.3. N2 Cold Surface

[315]:
```
bnormncs1 = map(f1 -> βjl(0.1, valst("n2", 0, temp="c")..., f1),
    fun(0.1, valst("n2", 0, temp="c")) .* fnorm) ./ ωun(0.1, valst("n2", 0, temp="c"));
```

[316]:
```
bnormncs05 = map(f1 -> βjl(0.05, valst("n2", 0, temp="c")..., f1),
    fun(0.05, valst("n2", 0, temp="c")) .* fnorm) ./ ωun(0.05, valst("n2", 0, temp="c"));
```

[317]:
```
bnormncs01 = map(f1 -> βjl(0.01, valst("n2", 0, temp="c")..., f1),
    fun(0.01, valst("n2", 0, temp="c")) .* fnorm) ./ ωun(0.01, valst("n2", 0, temp="c"));
```

8.8.4. O2 Cold Surface

[318]:
```
bnormocs1 = map(f1 -> βjl(0.1, valst("o2", 0, temp="c")..., f1),
    fun(0.1, valst("o2", 0, temp="c")) .* fnorm) ./ ωun(0.1, valst("o2", 0, temp="c"));
```

[319]:
```
bnormocs05 = map(f1 -> βjl(0.05, valst("o2", 0, temp="c")..., f1),
    fun(0.05, valst("o2", 0, temp="c")) .* fnorm) ./ ωun(0.05, valst("o2", 0, temp="c"));
```

[320]:
```
bnormocs01 = map(f1 -> βjl(0.01, valst("o2", 0, temp="c")..., f1),
    fun(0.01, valst("o2", 0, temp="c")) .* fnorm) ./ ωun(0.01, valst("o2", 0, temp="c"));
```

8.8.5. N2 Depth 1000 m

[321]:
```
bnormn1k1 = map(f1 -> βjl(0.1, valst("n2", 1000)..., f1),
    fun(0.1, valst("n2", 1000)) .* fnorm) ./ ωun(0.1, valst("n2", 1000));
```

[322]:
```
bnormn1k05 = map(f1 -> βjl(0.05, valst("n2", 1000)..., f1),
    fun(0.05, valst("n2", 1000)) .* fnorm) ./ ωun(0.05, valst("n2", 1000));
```

[323]:
```
bnormn1k01 = map(f1 -> βjl(0.01, valst("n2", 1000)..., f1),
    fun(0.01, valst("n2", 1000)) .* fnorm) ./ ωun(0.01, valst("n2", 1000));
```

8.8.6. O2 Depth 1000 m

[324]:
```
bnormo1k1 = map(f1 -> βjl(0.1, valst("o2", 1000)..., f1),
    fun(0.1, valst("o2", 1000)) .* fnorm) ./ ωun(0.1, valst("o2", 1000));
```

[325]:
```
bnormo1k05 = map(f1 -> βjl(0.05, valst("o2", 1000)..., f1),
    fun(0.05, valst("o2", 1000)) .* fnorm) ./ ωun(0.05, valst("o2", 1000));
```

[326]:
```
bnormo1k01 = map(f1 -> βjl(0.01, valst("o2", 1000)..., f1),
    fun(0.01, valst("o2", 1000)) .* fnorm) ./ ωun(0.01, valst("o2", 1000));
```

8.8.7. N2 Depth 2000 m

[327]:
```
bnormn2k1 = map(f1 -> βjl(0.1, valst("n2", 2000)..., f1),
    fun(0.1, valst("n2", 2000)) .* fnorm) ./ ωun(0.1, valst("n2", 2000));
```

[328]:
```
bnormn2k05 = map(f1 -> βjl(0.05, valst("n2", 2000)..., f1),
    fun(0.05, valst("n2", 2000)) .* fnorm) ./ ωun(0.05, valst("n2", 2000));
```

[329]:
```
bnormn2k01 = map(f1 -> βjl(0.01, valst("n2", 2000)..., f1),
    fun(0.01, valst("n2", 2000)) .* fnorm) ./ ωun(0.01, valst("n2", 2000));
```

8.8.8. O2 Depth 2000 m

[330]:
```
bnormo2k1 = map(f1 -> βjl(0.1, valst("o2", 2000)..., f1),
    fun(0.1, valst("o2", 2000)) .* fnorm) ./ ωun(0.1, valst("o2", 2000));
```

[331]:
```
bnormo2k05 = map(f1 -> βjl(0.05, valst("o2", 2000)..., f1),
    fun(0.05, valst("o2", 2000)) .* fnorm) ./ ωun(0.05, valst("o2", 2000));
```

[332]:
```
bnormo2k01 = map(f1 -> βjl(0.01, valst("o2", 2000)..., f1),
    fun(0.01, valst("o2", 2000)) .* fnorm) ./ ωun(0.01, valst("o2", 2000));
```

8.8.9. N2 Deep

[333]:
```
bnormnd1 = map(f1 -> βjl(0.1, valst("n2", 3500)..., f1),
    fun(0.1, valst("n2", 3500)) .* fnorm) ./ ωun(0.1, valst("n2", 3500));
```

[334]:
```
bnormnd05 = map(f1 -> βjl(0.05, valst("n2", 3500)..., f1),
    fun(0.05, valst("n2", 3500)) .* fnorm) ./ ωun(0.05, valst("n2", 3500));
```

[335]:
```
bnormnd01 = map(f1 -> βjl(0.01, valst("n2", 3500)..., f1),
    fun(0.01, valst("n2", 3500)) .* fnorm) ./ ωun(0.01, valst("n2", 3500));
```

8.8.10. O2 Deep

[336]:
```
bnormod1 = map(f1 -> βjl(0.1, valst("o2", 3500)..., f1),
    fun(0.1, valst("o2", 3500)) .* fnorm) ./ ωun(0.1, valst("o2", 3500));
```

[337]:
```
bnormod05 = map(f1 -> βjl(0.05, valst("o2", 3500)..., f1),
    fun(0.05, valst("o2", 3500)) .* fnorm) ./ ωun(0.05, valst("o2", 3500));
```

[338]:
```
bnormod01 = map(f1 -> βjl(0.01, valst("o2", 3500)..., f1),
    fun(0.01, valst("o2", 3500)) .* fnorm) ./ ωun(0.01, valst("o2", 3500));
```

8.9. Figure 6

[339]:
```
heights = [2, 2, 2, 2, 2, 1.5]



fig = figure(constrained_layout=true, dpi=300, figsize=(7.38,5.21))
gs = fig.add_gridspec(6, 4, height_ratios=heights)
ax1 = fig.add_subplot(get(gs, (0, slice(0, 2))))
ax1.loglog(fnorm, bnormns01, linestyle="-", color=get_cmap("plasma")(0.7), label="0.01")
ax1.loglog(fnorm, bnormns05, linestyle="-", color=get_cmap("plasma")(0.4), label="0.05")
ax1.loglog(fnorm, bnormns1, linestyle="-", color=get_cmap("plasma")(0), label="0.10")
ax1.set_ylabel(L"$\beta'$")
ax1.set_xticklabels(())

ax2 = fig.add_subplot(get(gs, (0, slice(2, 4))))
ax2.loglog(fnorm, bnormos01, linestyle="-", color=get_cmap("plasma")(0.7))
ax2.loglog(fnorm, bnormos05, linestyle="-", color=get_cmap("plasma")(0.4))
ax2.loglog(fnorm, bnormos1, linestyle="-", color=get_cmap("plasma")(0))
ax2.set_xticklabels(())
ax2.set_yticklabels(())

ax3 = fig.add_subplot(get(gs, (1, slice(0, 2))))
```

```
ax3.loglog(fnorm, bnormncs01, linestyle="-", color=get_cmap("plasma")(0.7))
ax3.loglog(fnorm, bnormncs05, linestyle="-", color=get_cmap("plasma")(0.4))
ax3.loglog(fnorm, bnormncs1, linestyle="-", color=get_cmap("plasma")(0))
ax3.set_ylabel(L"$\beta'$")
ax3.set_xticklabels(())

ax4 = fig.add_subplot(get(gs, (1, slice(2, 4))))
ax4.loglog(fnorm, bnormocs01, linestyle="-", color=get_cmap("plasma")(0.7))
ax4.loglog(fnorm, bnormocs05, linestyle="-", color=get_cmap("plasma")(0.4))
ax4.loglog(fnorm, bnormocs1, linestyle="-", color=get_cmap("plasma")(0))
ax4.set_xticklabels(())
ax4.set_yticklabels(())

ax5 = fig.add_subplot(get(gs, (2, slice(0, 2))))
ax5.loglog(fnorm, bnormn1k01, linestyle="-", color=get_cmap("plasma")(0.7))
ax5.loglog(fnorm, bnormn1k05, linestyle="-", color=get_cmap("plasma")(0.4))
ax5.loglog(fnorm, bnormn1k1, linestyle="-", color=get_cmap("plasma")(0))
ax5.set_ylabel(L"$\beta'$")
ax5.set_xticklabels(())

ax6 = fig.add_subplot(get(gs, (2, slice(2, 4))))
ax6.loglog(fnorm, bnormo1k01, linestyle="-", color=get_cmap("plasma")(0.7))
ax6.loglog(fnorm, bnormo1k05, linestyle="-", color=get_cmap("plasma")(0.4))
ax6.loglog(fnorm, bnormo1k1, linestyle="-", color=get_cmap("plasma")(0))
ax6.set_xticklabels(())
ax6.set_yticklabels(())

ax7 = fig.add_subplot(get(gs, (3, slice(0, 2))))
ax7.loglog(fnorm, bnormn2k01, linestyle="-", color=get_cmap("plasma")(0.7))
ax7.loglog(fnorm, bnormn2k05, linestyle="-", color=get_cmap("plasma")(0.4))
ax7.loglog(fnorm, bnormn2k1, linestyle="-", color=get_cmap("plasma")(0))
ax7.set_ylabel(L"$\beta'$")
ax7.set_xticklabels(())

ax8 = fig.add_subplot(get(gs, (3, slice(2, 4))))
ax8.loglog(fnorm, bnormo2k01, linestyle="-", color=get_cmap("plasma")(0.7))
ax8.loglog(fnorm, bnormo2k05, linestyle="-", color=get_cmap("plasma")(0.4))
ax8.loglog(fnorm, bnormo2k1, linestyle="-", color=get_cmap("plasma")(0))
ax8.set_xticklabels(())
ax8.set_yticklabels(())

ax9 = fig.add_subplot(get(gs, (4, slice(0, 2))))
ax9.loglog(fnorm, bnormnd01, linestyle="-", color=get_cmap("plasma")(0.7))
ax9.loglog(fnorm, bnormnd05, linestyle="-", color=get_cmap("plasma")(0.4))
ax9.loglog(fnorm, bnormnd1, linestyle="-", color=get_cmap("plasma")(0))
ax9.set_xlabel("Normalized Frequency")
ax9.set_ylabel(L"$\beta'$")
```

```
ax10 = fig.add_subplot(get(gs, (4, slice(2, 4))))
ax10.loglog(fnorm, bnormod01, linestyle="-", color=get_cmap("plasma")(0.7))
ax10.loglog(fnorm, bnormod05, linestyle="-", color=get_cmap("plasma")(0.4))
ax10.loglog(fnorm, bnormod1, linestyle="-", color=get_cmap("plasma")(0))
ax10.set_xlabel("Normalized Frequency")
ax10.set_yticklabels(())

ax1.get_shared_x_axes().join(ax1, ax3, ax5, ax7, ax9)
ax2.get_shared_x_axes().join(ax2, ax4, ax6, ax8, ax10)
ax1.get_shared_y_axes().join(ax1, ax2)
ax3.get_shared_y_axes().join(ax3, ax4)
ax5.get_shared_y_axes().join(ax5, ax6)
ax7.get_shared_y_axes().join(ax7, ax8)
ax9.get_shared_y_axes().join(ax9, ax10)

fig.text(0.092, 0.97, "A")
fig.text(0.554, 0.97, "B")
fig.text(0.092, 0.805, "C")
fig.text(0.554, 0.805, "D")
fig.text(0.092, 0.64, "E")
fig.text(0.554, 0.64, "F")
fig.text(0.092, 0.475, "G")
fig.text(0.554, 0.475, "H")
fig.text(0.092, 0.31, "I")
fig.text(0.554, 0.31, "J")
fig.legend(title="Bubble Radius (m)", ncol=4, loc="lower center");
```

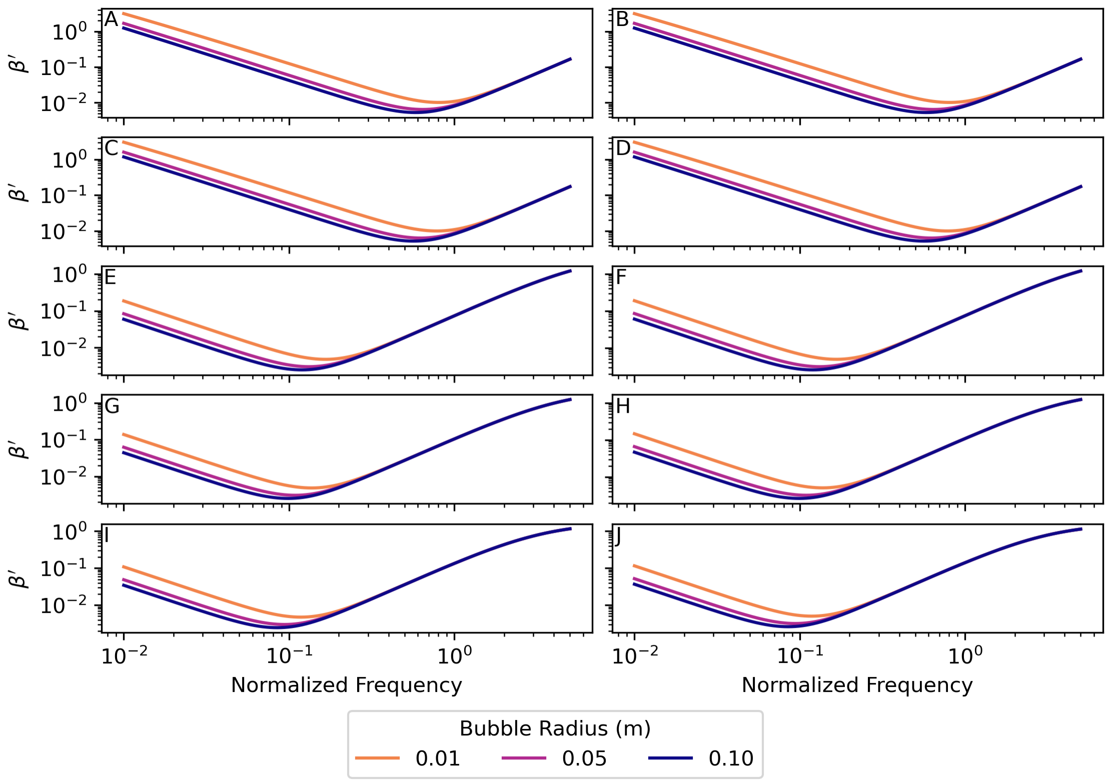


```
[340]: fig.savefig("Figure6.tiff", bbox_inches="tight")
fig.savefig("Figure6.png", bbox_inches="tight")
```

9. Quality Factor

Here we investigate the dependence of the quality factor on bubble radius.

9.1. Definition

Define a function for the quality factor as given in Thornton and Marion [43] using the far-field resonance frequency.

```
[341]: """
    Qf(a1, val)
The quality factor of a bubbles witht he supplied arguments.
"""
Qf(a1, val) = 2 * π * fff(a1, val)/(2 * βjl(a1, val..., fff(a1, val)))
```

```
[341]: Qf
```

Calculate quality factors at the extremes of the bubble radii considered.

[342]:
```python
Qf(0.01, valst("n2", 0))
```

[342]: 45.8833001348617

[343]:
```python
Qf(0.20, valst("n2", 0))
```

[343]: 65.43536388282477

[344]:
```python
Qf(0.01, valst("o2", 0))
```

[344]: 46.00104564779643

[345]:
```python
Qf(0.20, valst("o2", 0))
```

[345]: 65.55273228197672

[346]:
```python
Qf(0.01, valst("n2", 0, temp="c"))
```

[346]: 45.53819056712988

[347]:
```python
Qf(0.20, valst("n2", 0, temp="c"))
```

[347]: 63.319808487786524

[348]:
```python
Qf(0.01, valst("o2", 0, temp="c"))
```

[348]: 45.56558295488697

[349]:
```python
Qf(0.20, valst("o2", 0, temp="c"))
```

[349]: 63.36244884016511

[350]:
```python
Qf(0.01, valst("n2", 1000))
```

[350]: 6.8360329452990385

[351]:
```python
Qf(0.20, valst("n2", 1000))
```

[351]: 6.849065859618785

[352]:
```python
Qf(0.01, valst("o2", 1000))
```

[352]: 6.724019549208696

[353]:
```python
Qf(0.20, valst("o2", 1000))
```

[353]: 6.736932006913363

[354]:
```python
Qf(0.01, valst("n2", 2000))
```

[354]: 4.8151815800987094

[355]:
```
Qf(0.20, valst("n2", 2000))
```

[355]: 4.819975037108813

[356]:
```
Qf(0.01, valst("o2", 2000))
```

[356]: 4.6233302573131985

[357]:
```
Qf(0.20, valst("o2", 2000))
```

[357]: 4.628031634794526

[358]:
```
Qf(0.01, valst("n2", 3500))
```

[358]: 3.78212180286386

[359]:
```
Qf(0.20, valst("n2", 3500))
```

[359]: 3.78445001168391

[360]:
```
Qf(0.01, valst("o2", 3500))
```

[360]: 3.6156980422088743

[361]:
```
Qf(0.20, valst("o2", 3500))
```

[361]: 3.617974516054981

9.2. Figure 7

Now plot the quality factor vs. bubble radius for different environments. Note that here we calculate the arrays of values within the `plot` function.

[362]:
```
fig = figure(dpi=300, figsize=[5.5,3.5])
plot(avals, map(a1 -> Qf(a1, valst("n2", 0)), avals), linestyle="-",␣
  ↪color=get_cmap("plasma")(0), label=L"N$_2$ Warm Surface")
plot(avals, map(a1 -> Qf(a1, valst("o2", 0)), avals), linestyle="--",␣
  ↪color=get_cmap("plasma")(0.1), label=L"O$_2$ Warm Surface")
plot(avals, map(a1 -> Qf(a1, valst("n2", 0, temp="c")), avals), linestyle="-",␣
  ↪color=get_cmap("plasma")(0.2), label=L"N$_2$ Cold Surface")
plot(avals, map(a1 -> Qf(a1, valst("o2", 0, temp="c")), avals), linestyle="--",␣
  ↪color=get_cmap("plasma")(0.3), label=L"O$_2$ Cold Surface")
plot(avals, map(a1 -> Qf(a1, valst("n2", 1000)), avals), linestyle="-",␣
  ↪color=get_cmap("plasma")(0.4), label=L"N$_2$ 1000 m Deep")
plot(avals, map(a1 -> Qf(a1, valst("o2", 1000)), avals), linestyle="--",␣
  ↪color=get_cmap("plasma")(0.5), label=L"O$_2$ 1000 m Deep")
```

```
plot(avals, map(a1 -> Qf(a1, valst("n2", 2000)), avals), linestyle="-",␣
    ↪color=get_cmap("plasma")(0.6), label=L"N$_2$ 2000 m Deep")
plot(avals, map(a1 -> Qf(a1, valst("o2", 2000)), avals), linestyle="--",␣
    ↪color=get_cmap("plasma")(0.7), label=L"O$_2$ 2000 m Deep")
plot(avals, map(a1 -> Qf(a1, valst("n2", 3500)), avals), linestyle="-",␣
    ↪color=get_cmap("plasma")(0.8), label=L"N$_2$ 3500 m Deep")
plot(avals, map(a1 -> Qf(a1, valst("o2", 3500)), avals), linestyle="--",␣
    ↪color=get_cmap("plasma")(0.9), label=L"O$_2$ 3500 m Deep")
xlabel("Bubble Radius (m)")
ylabel(L"Quality Factor $Q_f$")
xlim([-0.005, 0.205])
PyPlot.xticks([0, 0.05, 0.10, 0.15, 0.20])
ylim([-1, 67])
#PyPlot.yticks([0, 10, 20, 30, 40, 50, 60])
legend(bbox_to_anchor=[1.05,1],loc=2,borderaxespad=0);
```

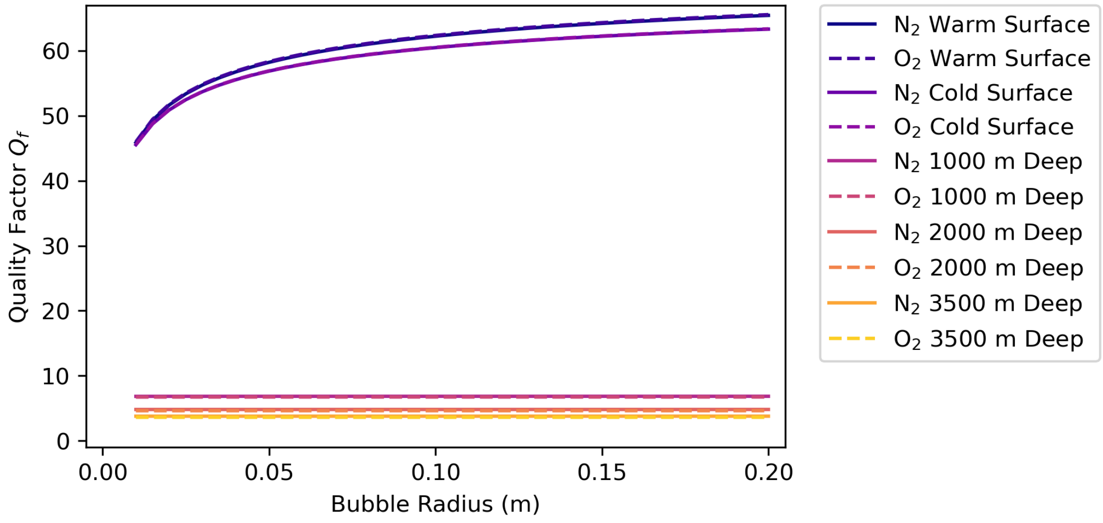

[363]:
```
fig.savefig("Figure7.tiff", bbox_inches="tight")
fig.savefig("Figure7.png", bbox_inches="tight")
```

10. Power Loss

Here we investigate the power loss of a bubble at a given depth compared to the same bubble at the surface. We do these calculations for a water profile with a constant temperature water of 1.5 °C, the temperature of cold surface environment and the deep water environment.

10.1. Definition

The power loss is a ratio of the resonant scattering cross section at depth to the resonant scattering cross section at the surface.

[364]:
```
"""
    pl(a1, v1, v0)
Power loss function for a bubble of radius `a1`. `v1` is the parameter
list at the bubble depth, and `v0` is the parameter list for a bubble at
the water surface.
"""
pl(a1, v1, v0) = σres(a1, v1)/σres(a1, v0)
```

[364]: pl

[365]:
```
10*log10(pl(0.1, valsn2list(1000), valsn2list(0)))
```

[365]: -19.046060802750176

10.2 Figure 8

Now plot the power loss in decibels vs. depth for different bubble radii. Note that here we calculate the arrays of values within the `plot` function.

[366]:
```
fig = figure(dpi=300, figsize=[5.5,3.5])
plot(dvals, map(d1 -> 10*log10(pl(0.01, valsn2list(d1), valsn2list(0))), dvals),
    linestyle="-", color=get_cmap("plasma")(0.7), label=L"0.01, N$_2$")
plot(dvals, map(d1 -> 10*log10(pl(0.01, valso2list(d1), valso2list(0))), dvals),
    linestyle="--", color=get_cmap("plasma")(0.7), label=L"0.01, O$_2$")
plot(dvals, map(d1 -> 10*log10(pl(0.05, valsn2list(d1), valsn2list(0))), dvals),
    linestyle="-", color=get_cmap("plasma")(0.4), label=L"0.05, N$_2$")
plot(dvals, map(d1 -> 10*log10(pl(0.05, valso2list(d1), valso2list(0))), dvals),
    linestyle="--", color=get_cmap("plasma")(0.4), label=L"0.05, O$_2$")
plot(dvals, map(d1 -> 10*log10(pl(0.1, valsn2list(d1), valsn2list(0))), dvals),
    linestyle="-", color=get_cmap("plasma")(0), label=L"0.1, N$_2$")
plot(dvals, map(d1 -> 10*log10(pl(0.1, valso2list(d1), valso2list(0))), dvals),
    linestyle="--", color=get_cmap("plasma")(0), label=L"0.1, O$_2$")
xlabel("Depth (m)")
ylabel("Power Reduction (dB)")
legend(title="Radius (m), Gas");
```

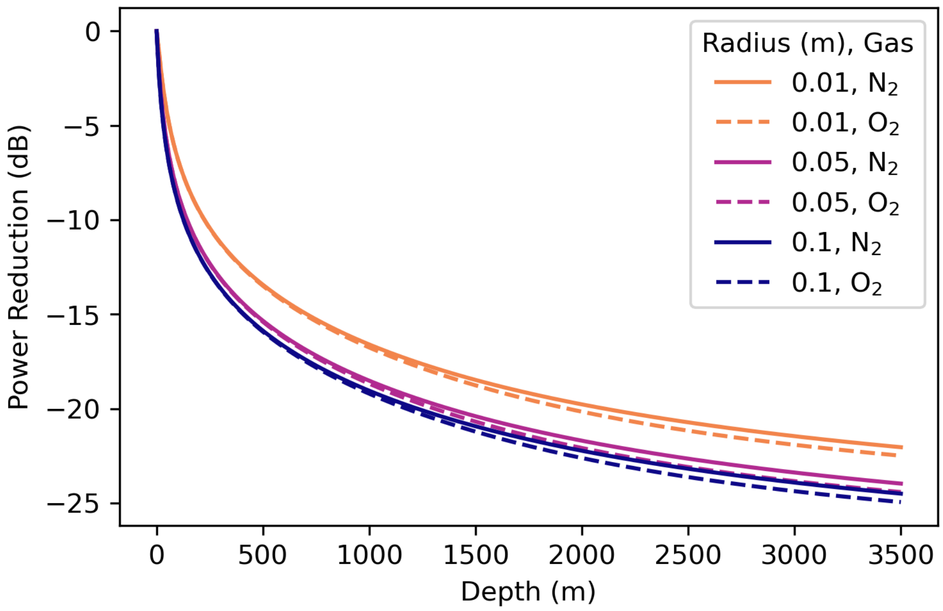

[367]:

```
fig.savefig("Figure8.tiff", bbox_inches="tight")
fig.savefig("Figure8.png", bbox_inches="tight")
```

[ ]:

```
```
