# Supplementary material for: An investigation of bubble resonance and its implications for sound production by deep-water fishes
Source: PLoS One. 2022 Jul 12;17(7):e0267338. doi: 10.1371/journal.pone.0267338 (PMC9275728; doi:10.1371/journal.pone.0267338)
Supplement: Attachment — Submitted filename: Response to Reviewers.pdf [file pone.0267338.s005.pdf]

Mark W. Sprague, Ph.D. | Associate Professor | Department of Physics
Mail Stop 563 | East Carolina University | Greenville, NC 27858-4353 | USA
252-328-1862 office | 252-328-6314 fax | spraguem@ecu.edu | myweb.ecu.edu/spraguem

June 14, 2022

Editor
PLOS ONE
1265 Battery Street, Suite 200
San Francisco, CA 94111
United States
https://journals.plos.org/plosone/

Dear Editor:

Thank you for the reviews of our manuscript "An investigation of bubble resonance and its implications for sound production by deep-water fishes" (PONE-D-22-10057) submitted to PLOS ONE. We have considered all of the reviewers' comments and include our revised manuscript in marked-up form as well as a clean copy. Below we address each of the reviewers' comments.

Reviewer 1

Reviewer 1 recommended publication without changes.

Reviewer 2

Reviewer 2 recommended that we address conditions at various depths. The original manuscript included calculations for a warm (20 °C) surface environment and a cold (1.5 °C) surface environment as well as an environment at depth 3500 m. Additionally, there were calculations and figures with depth varying from the surface to 3500 m. Our revision adds calculations for depths 1000 m and 2000 m to the other environments. We added the conditions at these depths to Table 1 and included the calculations in all figures that compare properties in the different environments. We believe that adding these depth calculations to the paper strengthens it significantly because it provides information and relevancy for those studying soniferous fishes at depths less than 3500 m. Also, the inclusion of the intermediate depths demonstrates the transition of bubble properties between the surface and the extreme depth of 3500 m.

Reviewer 2 also recommended that we expand on how to gather empirical data to support our calculations. One of the reasons that we performed this numerical study is that sound measurements at extreme depths is so difficult. Nonetheless, we added a paragraph to the discussion section about gathering empirical data.

Reviewer 3

Reviewer 3 labeled their comments with letters and numbers, and we address each comment below using the same labels.

- a-1 Reviewer 3 asked us to explain and discuss the “novel” word in the abstract. We do not understand this comment. The word “novel” was not used in the abstract. We believe that although our approach is novel, no changes to the abstract with respect to this comment are necessary.

- a-2 Reviewer 3 asked that we include a table of main and boundary conditions including flow rate, temperature, *etc.* We added a sentence at the end of the first paragraph in Section 1.1 stating that we assume zero flow rate into and around the bubbles. However, since we use well-established theory in our references, we do not believe that a table of boundary conditions used for the development of the theory of bubble radiation would be appropriate in this paper. Readers can refer to our references [particularly Ainslie and Leighton (2011 and 2009)] for a full theoretical development.

- a-3 Reviewer 3 asked us to include an algorithm and schematic form of numerical solution. We have included this in a new Section 2 Computational Methods to satisfy this concern.

- a-4 Reviewer 3 asked us to include modeling of droplets in our literature review and included a link to an excellent paper on numerical modeling of heterogeneous nucleation in wet steam flow. We do not believe that this is necessary in or improves understanding of our paper. Our calculations are for acoustic radiation of gas bubbles under water in otherwise quiescent conditions. In the literature on underwater bubble radiation theory, there are no manuscripts that mention or cite references to heterogeneous liquid droplets. We do not believe that this would improve the understanding of acoustic radiation of gas bubbles and have not added it to our literature review.

- b-1 Reviewer 3 asked us to polish both the language and structure of the manuscript. In addition to addressing the reviewer comments, we have revised the entire paper. We believe that the revised manuscript meets the standards for publication in PLOS ONE.

We thank the reviewers and believe we have addressed their comments successfully. Hopefully the manuscript is now acceptable for publication. Thank you for your attention.

Sincerely,

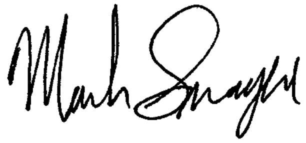

Mark W. Sprague

cc: Michael L. Fine, Timothy M. Cameron

encl: Revised manuscript with changes marked,
Clean copy of revised manuscript
